# Epigenome-wide DNA methylation analysis of small cell lung cancer cell lines suggests potential chemotherapy targets

**DOI:** 10.1186/s13148-020-00876-8

**Published:** 2020-06-25

**Authors:** Julia Krushkal, Thomas Silvers, William C. Reinhold, Dmitriy Sonkin, Suleyman Vural, John Connelly, Sudhir Varma, Paul S. Meltzer, Mark Kunkel, Annamaria Rapisarda, David Evans, Yves Pommier, Beverly A. Teicher

**Affiliations:** 1 Biometric Research Program, Division of Cancer Treatment and Diagnosis, National Cancer Institute, NIH, 9609 Medical Center Dr., Rockville, MD 20850 USA; 2 Molecular Pharmacology Group, Leidos Biomedical Research, Inc., Frederick National Laboratory for Cancer Research, Frederick, MD 21702 USA; 3 Developmental Therapeutics Branch, Center for Cancer Research, National Cancer Institute, NIH, Bethesda, MD 20892 USA; 4 Genetics Branch, Center for Cancer Research, National Cancer Institute, Bethesda, MD 20892 USA; 5 Drug Synthesis and Chemistry Branch, Division of Cancer Treatment and Diagnosis, National Cancer Institute, Bethesda, MD 20892 USA; 6 Molecular Pharmacology Program, Division of Cancer Treatment and Diagnosis, National Cancer Institute, Bethesda, MD 20892 USA

**Keywords:** DNA methylation, Small cell lung cancer, Gene expression, Epigenetic regulation, Chemotherapy

## Abstract

**Background:**

Small cell lung cancer (SCLC) is an aggressive neuroendocrine lung cancer. SCLC progression and treatment resistance involve epigenetic processes. However, links between SCLC DNA methylation and drug response remain unclear. We performed an epigenome-wide study of 66 human SCLC cell lines using the Illumina Infinium MethylationEPIC BeadChip array. Correlations of SCLC DNA methylation and gene expression with in vitro response to 526 antitumor agents were examined.

**Results:**

We found multiple significant correlations between DNA methylation and chemosensitivity. A potentially important association was observed for *TREX1*, which encodes the 3′ exonuclease I that serves as a STING antagonist in the regulation of a cytosolic DNA-sensing pathway. Increased methylation and low expression of *TREX1* were associated with the sensitivity to Aurora kinase inhibitors AZD-1152, SCH-1473759, SNS-314, and TAK-901; the CDK inhibitor R-547; the Vertex ATR inhibitor Cpd 45; and the mitotic spindle disruptor vinorelbine. Compared with cell lines of other cancer types, *TREX1* had low mRNA expression and increased upstream region methylation in SCLC, suggesting a possible relationship with SCLC sensitivity to Aurora kinase inhibitors.

We also identified multiple additional correlations indicative of potential mechanisms of chemosensitivity. Methylation of the 3′UTR of *CEP350* and *MLPH*, involved in centrosome machinery and microtubule tracking, respectively, was associated with response to Aurora kinase inhibitors and other agents. *EPAS1* methylation was associated with response to Aurora kinase inhibitors, a PLK-1 inhibitor and a Bcl-2 inhibitor. *KDM1A* methylation was associated with PLK-1 inhibitors and a KSP inhibitor. Increased promoter methylation of *SLFN11* was correlated with resistance to DNA damaging agents, as a result of low or no *SLFN11* expression. The 5′ UTR of the epigenetic modifier *EZH2* was associated with response to Aurora kinase inhibitors and a FGFR inhibitor. Methylation and expression of *YAP1* were correlated with response to an mTOR inhibitor. Among non-neuroendocrine markers, *EPHA2* was associated with response to Aurora kinase inhibitors and a PLK-1 inhibitor and *CD151* with Bcl-2 inhibitors.

**Conclusions:**

Multiple associations indicate potential epigenetic mechanisms affecting SCLC response to chemotherapy and suggest targets for combination therapies. While many correlations were not specific to SCLC lineages, several lineage markers were associated with specific agents.

## Introduction

Small cell lung cancer (SCLC) is a highly aggressive neuroendocrine tumor prone to early metastasis, short survival, and limited options for effective treatment [1–3]. Despite an unmet need to identify new therapies, progress in SCLC treatment has been hindered by rapidly acquired resistance to therapy resulting in limited and transient response to second and third line chemotherapeutic and immunotherapeutic agents [2]. Recently, the US FDA approved the immunotherapy drugs atezolizumab, pembrolizumab, and nivolumab for the treatment of recurrent SCLC [4].

Genome studies have identified frequent somatic molecular alterations in SCLC cells including functional inactivation of *TP53*, *RB1*, and, less commonly, *PTEN* tumor suppressor genes; copy number amplification of *MYC* family genes *MYC*, *MYCL1*, and *MYCN*; mutations in the *EP300*, *CREBBP*, and *KMT2A* (*MLL*) and *KMT2D (MLL2)* genes encoding histone-modifying proteins; inactivating mutations in *NOTCH* family genes; and common loss of genomic regions containing *FHIT* and *CDKN2A* genes [2, 5–8]. Other genomic alterations found in SCLC specimens include somatic rearrangements of the *TP73* gene and overexpression of *CCND1*, mutations in *SLIT2* and *EPHA7*, and focal amplifications of *FGFR1* [5, 6]. Smoking-associated signatures in SCLC tumors have also been reported [9, 10].

Recent molecular studies have established that SCLC lineages fall into a number of distinct subtypes, currently referred to as SCLC-A, SCLC-N, SCLC-Y, and SCLC-P, based on their differences in gene and protein expression of transcriptional molecular neuroendocrine, non-neuroendocrine, or tuft cell-like lineage regulators ASCL1, NEUROD1, INSM1, YAP1, and POU2F3, and of their downstream molecular targets [2]. Whether these SCLC subtypes respond differently to specific treatments and whether patient tumors may represent a heterogeneous mix of SCLC lineages remains a subject of active investigation [2].

There is a growing understanding that many changes associated with SCLC carcinogenesis may be driven by epigenetic processes. Genes encoding several epigenetic factors including histone acetyltransferases EP300 and CREBBP, and histone methyltransferases KMT2A and KMT2D are frequently mutated in SCLC tumors [2, 5, 6, 11]. High expression of another histone methyltransferase gene, *EZH2*, is a distinct feature of SCLC when compared to normal lung tissue or other cancer categories [11]. The transcriptional master regulator, *POU2F3*, which defines the tuft cell-like SCLC lineage [2, 12, 13], is epigenetically silenced in cervical cancer via hypermethylation of the *POU2F3* promoter [14], suggesting the possibility that DNA methylation-mediated regulatory mechanisms could play a role in SCLC development and progression. A survey of global methylation patterns in primary SCLC tumors and SCLC cell lines identified 73 potential gene targets enriched for binding sites of cell fate-specifying transcriptional factors [15].

Despite growing evidence for the role of epigenetic factors in SCLC cancer, understanding their influence on tumor response to treatment remains limited. Earlier studies of other cancer histologies have identified therapeutically relevant DNA methylation biomarkers in the promoter regions of *MGMT*, increased methylation of which is beneficial for the response to alkylating therapeutic agents and ionizing radiation in glioblastoma and colorectal cancer, and of *SLFN11*, methylation of which has been associated with resistance to DNA damaging agents in a variety of cancer categories [16–22]. Insight into epigenetic modulation of SCLC response to DNA damaging agents was provided by the discovery of epigenetic silencing of *SLFN11* by EZH2 in the course of cisplatin-etoposide therapy, which may lead to treatment resistance or chemo-sensitive relapse [23]. Unfortunately, the role of epigenetic mechanisms in response to other agents and the effect of various epigenetic alterations on drug treatment response in SCLC remain largely unknown.

To provide insight into epigenetic factors which may influence the response of SCLC to treatment, an epigenome-wide DNA methylation analysis was performed. Methylation data were analyzed to determine how epigenomic states of gene regions, individual probes, and genes were associated with SCLC response to FDA-approved oncology drugs and about 400 investigational agents. These epigenome analyses utilized drug response and transcriptional profiling data obtained in our earlier study [24], in which we identified a number of gene transcripts and miRNAs associated with SCLC response to treatment. In the current report, we describe the use of high-density DNA methylation measures to identify epigenomic regions and gene targets that were strongly associated with response to a variety of therapeutic agents.

## Methods

### Drug response measures

We analyzed drug response measures obtained earlier in the in vitro screen of 526 FDA-approved and investigational agents using SCLC cell lines [24]. Sixty-six SCLC cell lines had both drug response information and DNA methylation measures and were included in the analysis of Spearman correlation between probe or gene region methylation levels and median values of the log(IC50) measures of drug response among cell line replicates (Supplementary Table 1). Among 526 agents, 412 drugs showed variability of response among the 66 SCLC cell lines and were included in association analysis.

Measurement of SCLC cell growth and drug response measures and the steps for quality control (QC) were described in detail previously [24]. Briefly, each agent was tested at nine concentrations (10 μM to 1.5 nM, with DMSO concentration of 0.25%), after a 96-h incubation with the cells. The statistical validity of the drug response dataset was evaluated by calculating the *Z*’ factor for each plate in the assay, with *Z*’ > 0.5 considered to be a high-quality assay. Concentration response data were fit with a 4-parameter curve, and median IC50 values for each agent were computed among cell line replicates.

### Methylation data processing

Methylation measurements for all cell lines were generated in a single batch using Illumina Infinium MethylationEPIC BeadChip (Illumina, Inc). The absence of batch effects was confirmed by comparing clustering of SCLC based on methylation data with that based on gene expression data.

Methylation data were normalized, and beta and detection *p* values were calculated using the minfi package [25] using default parameters, resulting in 866,091 methylation probe measurements. Methylation probe beta-values for individual cell lines with detection *p* values ≥ 10^−3^ and the entire 1427 probes with median detection *p* ≥ 10^−6^ were excluded from analysis. Probes overlapping with single nucleotide polymorphisms (SNPs) were filtered out according to the list of probe masking recommendations of Zhou et al. [26, 27]. The final methylation dataset used in correlation analysis with drug response and with gene expression had methylation beta-values for 760,637 probes that passed all filtering.

### Epigenome-wide analysis of association of DNA methylation with chemosensitivity

In order to compute gene region-averaged methylation beta-values for the Infinium MethylationEPIC BeadChip dataset, we developed an R program which followed the algorithm which had previously been developed by other authors for the IMA software package [28] for the analysis of Illumina 450K Infinium methylation array data. Briefly, we used the Infinium MethylationEPIC BeadChip annotation of each probe [29] according to the UCSC genome browser data to compute gene region-averaged methylation values for each of the following gene regions: TSS1500 (200–1500 bases upstream of the transcriptional start site, or TSS), TSS200 (0–200 bases upstream of the TSS), 5′UTR (within the 5′ untranslated region, between the TSS and the ATG start site), first exon, gene body (between the ATG start site and the stop codon), and 3′UTR (within the 3′ untranslated region, between the stop codon and poly A signal). Methylation of different gene regions was considered separately in association analyses and was not combined. The probes annotated as belonging to more than one region and/or more than one gene were included in calculation of average methylation values of each of their respective annotated gene regions. Statistical analysis was performed using the R environment v. 3.5.3. The resulting methylation values were computed for 108,795 regions belonging to 26,239 genes, transcripts, and miRNA listed in the Infinium MethylationEPIC BeadChip manifest annotation [29]. Chromosomal regions (cytoband) of each probe were identified according to the UCSC genome annotation database for the hg19 (GRCh37) assembly of the human genome based on probe coordinates in the Infinium MethylationEPIC BeadChip annotation [30].

Spearman correlation analysis of methylation measures with log(IC50) was performed for methylation beta-values of each individual methylation probe, and also for methylation values averaged among the probes within each of the six gene regions (TSS1500, TSS200, 5′ UTR, first exon, gene body, and 3′ UTR). Sixty-six SCLC cell lines, which had both drug response data and methylation measures, were included in correlation analysis. All analyses described in this report included only SCLC cell lines and did not include NSCLC or mesothelioma cell lines. Significance of correlation of methylation of gene regions with drug response was evaluated using the Benjamini-Hochberg adjustment procedure for false discovery rate (FDR) [17] using all *p* values from correlation tests of all 412 drug agents with variable drug response and 108,795 gene regions. For individual methylation probes, we used a fixed threshold of *p* < 9.42 × 10^−8^ for a single Illumina Infinium MethylationEPIC BeadChip array methylation probe, according to recently published recommendations [31]. In addition, we also compiled a broader list of top genes associated with drug response by combining the probes satisfying a more liberal threshold of *p* < 5 × 10^−7^, in an analogy with previously reported criteria of 10^−6^ for Illumina 450K array which had fewer probes than the Illumina Infinium MethylationEPIC BeadChip array [32]. We refer to the FDR-adjusted *p* values as *p*_FDR_ and the original *p* values prior to FDR adjustment as *p*_O_. Special attention was paid to the significant associations involving the upstream gene regions, which are most likely to contain the promoter regions and regulatory regions affecting gene expression and to individual probes located in the upstream gene regions.

The overlap between top results for different agents and genes was visualized using Venn diagrams which were constructed with the help of the public online version of DisplayR [33].

### Association of methylation of candidate genes with selected antitumor agents

In addition to epigenome-wide analysis of association of SCLC DNA methylation with drug response, we also focused more closely on possible epigenetic mechanisms of response to 44 anticancer agents (Supplementary Table 2). This list included agents that exhibited higher efficacy in subgroups of SCLC cell lines in the in vitro single agent screen, as well as agents with potential promise for activity against SCLC based on in vitro or preclinical results from other studies or based on their inclusion in SCLC clinical trials [24]. We examined association of log(IC50) of these agents with methylation of individual probes and gene regions of 78 genes representing drug-specific targets and 48 additional genes involved in drug target pathways (Supplementary Table 2). In addition, we analyzed association of methylation of log(IC50) of each of the 44 agents with methylation of individual probes and gene regions of 159 protein-coding genes that included genes with relevance to SCLC lineage determination; SCLC lineage markers; genes that carry frequent mutations or genome alterations in SCLC; genes which are commonly inactivated, overexpressed, or epigenetically modified in SCLC tumors or in specific SCLC subtypes; as well as genes previously reported as being involved in pathways leading to SCLC pathogenesis; or those suggested as being relevant to SCLC response to chemotherapy [3, 5, 11, 13, 15, 24, 34–39]. The list of these genes is provided in the legend to Supplementary Table 2. Each candidate gene was represented by multiple probes and up to six gene regions (TSS1500, TSS200, 5′UTR, first exon, gene body, and 3′UTR), which were analyzed independently from each other. The resulting correlation *p* values were FDR adjusted by combining the results for all 44 agents, separately for 10,515 methylation probes in or near the candidate genes and for 1376 gene regions in candidate genes. The adjustment for multiple testing and interpretation of the significance of the results derived from the analysis of candidate genes and regions were done separately and independently from the adjustment for multiple testing and interpretation of significance in the analysis at the epigenome-wide level, described above. The results obtained using these two approaches were presented separately.

### Association of DNA methylation with gene and miRNA expression and correlation of transcripts with drug response

We examined how association of SCLC DNA methylation of individual probes and gene regions with drug response may be related to expression of genes and miRNAs located in the same genome regions. For this purpose, we used gene expression and miRNA measurements generated and processed by an earlier study of our group [24], which generated transcript expression data using Affymetrix GeneChip®Human Exon 1.0 ST Arrays (NCBI GEO accession number GSE73160) and NanoString miRNA expression measurements (NCBI GEO accession GSE73161). Experimental and computational procedures for mRNA and miRNA data collection, processing, QC, data normalization, and adjustment for batch effects were reported previously [24, 38]. We used mRNA expression measures normalized using Robust Multi-Array Average (RMA) and summarized at the whole transcript level using AROMA [40]; we also utilized miRNA data which were normalized and log_2_ + 1 transformed [24]. Expression data for the total of 18,690 transcripts and 800 miRNAs were adjusted separately for batch effects using the ComBat function of the sva package [41]. The validity of adjustment was confirmed by hierarchical sample clustering using the hclust function of R v. 3.3.0. Pearson correlation was used to evaluate association of log_2_-transformed normalized expression values of each transcript and each miRNA with log(IC50) of each drug agent. In addition, we used Spearman correlation (Supplementary Table 1) to examine how the methylation beta-values of each of the top methylation probes and average methylation beta-values of gene regions associated with drug response were correlated with log_2_-transformed normalized expression measures of genes and miRNAs located in the same genome regions, based on Illumina Infinium MethylationEPIC BeadChip microarray annotation according to the UCSC genome browser data. Here and below, *ρ* stands for Spearman correlation coefficient and *r* stands for Pearson correlation coefficient.

### Copy number data

In cases when DNA methylation directly affects gene expression without copy number changes, a negative association between DNA methylation measures, most commonly in the upstream gene region, and gene expression may be expected [42]. Gene copy number gain commonly results in its overexpression, whereas copy number loss could lead to lower expression levels. Copy number events have been reported to result in positive or negative correlations between DNA methylation and gene expression measures depending on the probe location, with positive correlations more common in the gene body [42]. To examine possible causes of positive associations between DNA methylation and gene expression, we verified copy number information from the Cancer Cell Line Encyclopedia (CCLE) resource at the Broad Institute [43, 44] for selected genes in which one or more probes and/or gene regions were significantly associated with drug response in our data, and the same probes and/or gene regions were strongly positively correlated with gene expression values. Thirty-three SCLC cell lines with available methylation, transcript expression, miRNA expression, and drug response measurements in our dataset also had copy number data available from CCLE. Gene level copy number data had been generated by the CCLE Consortium using Affymetrix 6.0 SNP arrays, with segmentation of normalized log_2_ ratios of the copy number estimates performed using the circular binary segmentation algorithm [43, 44].

### Analysis of association of *TREX1* expression and methylation with drug response using data from other resources

Due to the absence of *TREX1* gene expression measurements among the transcript clusters derived from the Affymetrix GeneChip®Human Exon 1.0 ST Array, for this gene we used Affymetrix Human Genome U133 Plus 2.0 microarray measurements (probe 34689_at) available from the CCLE legacy portal [22, 45] for the 36 cell lines that were included both in our dataset and in the CCLE data. These microarray measures had an excellent correlation with *TREX1* RNA-seq expression measurements available from CCLE [43, 44] (Spearman correlation coefficient *ρ* ≥ 0.9135, Pearson *r* ≥ 0.9041, *p* ≤ 3.80 × 10^−20^ for all tests in SCLC cell lines and across cancer categories).

For validation of drug sensitivity associations with *TREX1* methylation and expression, we analyzed correlations of molecular measurements with drug response in 40 SCLC cell lines that had drug sensitivity data available from the Genomics of Drug Sensitivity in Cancer (GDSC) dataset [46, 47] and *TREX1* DNA methylation and gene expression measures available from CCLE. The independent *TREX1* methylation dataset in CCLE was generated using reduced representation bisulfite sequencing (RRBS). These data included the *TREX1* promoter region within 1 kb upstream of the TSS, promoter CpG clusters, and promoter CpG islands, as provided by the CCLE project [43] and described in detail in a recent report [44]. For *TREX1* gene expression measures, we used CCLE Affymetrix Human Genome U133 Plus 2.0 microarray data (probe 34689_at) [22, 45]. Drug sensitivity measurements (GDSC1 and GDSC2 datasets) were obtained from the Genomics of Drug Sensitivity in Cancer (GDSC) resource [46, 47].

### Association of methylation and gene expression with drug response in relation to SCLC lineage classification

To examine whether patterns of DNA methylation and transcript expression that were significantly correlated with drug response were also associated with SCLC lineage subgroups, we analyzed Spearman and Pearson correlation of DNA methylation of individual probes and gene regions with expression of six lineage SCLC markers, *ASCL1*, *ASCL2*, *NEUROD1*, *INSM1*, *YAP1*, and *POU2F3* [2, 48]. We also used hierarchical clustering of SCLC cell lines based on these six lineage markers to examine whether patterns of DNA methylation and gene expression in the genes of interest were different among SCLC clusters. Clustering of SCLC cell lines according to their lineage marker expression was performed using the “average” (UPGMA) option of the hclust command in the R environment based on Euclidian distances, with subsequent annotation of SCLC cell line cluster assignments according to a previous report [2] when such annotation was available.

## Results

Below, we first present the results of the epigenome-wide association analysis of individual probes and gene regions with all agents. We discuss the strongest associations of methylation of individual probes with drug response. We further discuss whether those associations were also in agreement with the correlations of methylation of regions of the same genes with drug response and whether such associations could be explained by the effect of DNA methylation on gene expression. We also highlight some of the top correlations of gene regions with drug response. In a separate section, we report the associations of methylation of the probes and regions in the candidate genes with response to candidate drug agents. We highlight their strongest correlations and also discuss specific genes of particular biological interest. Detailed information about all significant associations is provided in Supplementary Tables 3, 4, 5, 6, 7 and 8. Additional details are provided in Supplementary Data 1, 2, 3 and 4.

### Association of DNA methylation of probes and gene regions with drug response at the epigenome-wide level of significance

Spearman correlation analysis between the beta-values of methylation probes that passed QC and SNP filtering and log(IC50) of drug agents identified 294 strong correlations with *p* < 9.42 × 10^−8^; all of them had Spearman correlation coefficient |*ρ*| > 0.6 (Supplementary Tables 3 and 4)*.* The summary of genes containing the top probe correlations with drug agents (*p* < 10^−8^) is presented in Table 1. Table 2 provides the list of significant (*p*_FDR_ < 0.05) and nearly significant (*p*_FDR_ < 0.1) gene regions associated with drug response.

**Table 1:** Individual genes and genome regions containing multiple genes which contained methylation probes with the strongest Spearman correlations (*p* < 10^−8^) with log(IC50) of antitumor agents

Agent	Inhibitor category	Associated genes	Chromosomal location	Highest |***ρ***| for probe methylation vs log(IC50)	Lowest ***p*** for probe methylation vs log(IC50)	Highest |***r***| for transcript expression vs log(IC50)	Lowest ***p*** for transcript expression vs logIC50	Highest |***ρ***| for transcript expression vs probe methylation	Lowest ***p*** for transcript expression vs probe methylation	Transcript with lowest ***p*** for expression vs log(IC50)
4SC-202	HDAC	***SNED1***	2q37.3	0.6927	1.16 × 10^−10^	0.1492	0.2281	0.2178	0.0789	NM_001080437 (*SNED1*)
BIM-46187	Pan-G-protein	***C8orf74***	8p23.1	− 0.6721	6.47 × 10^−10^					
R-547	CDK	***TREX1***	3p21.31	− 0.6674	9.39 × 10^−10^					
CT-32228	LPAAT-β	***STK39***	2q24.3	0.6620	1.43 × 10^−9^	− 0.0575	0.6438	− 0.1757	0.1581	NM_013233 (*STK39*)
Pyrvinium pamoate		***FBXL18***	7p22.1	0.6612	1.51 × 10^−9^	0.0083	0.9471	− 0.0427	0.7333	NM_024963 (*FBXL18*)
Digoxin		***KLHL26***	19p13.11	0.6606	1.58 × 10^−9^	0.0266	0.8309	0.2699	0.0284	NM_018316 (*KLHL26*)
Astex FGF inhibitor	FGFR	***TRIM2***	4q31.3	0.6580	1.94 × 10^−9^	0.0143	0.9085	− 0.1558	0.2117	NM_015271 (*TRIM2*)
ENMD-2076	Aurora kinase	***NRP1***	10p11.22	0.6566	2.14 × 10^−9^	0.1231	0.3212	0.3968	0.0010	NM_003873 (*NRP1*)
Olaparib	PARP1	***LHX4***	1q25.2	0.6559	2.26 × 10^−9^	0.0803	0.5184	0.4303	0.0003	NM_033343 (*LHX4*)
Depsipeptide	HDAC	***VWF***	12p13.31	− 0.6535	2.70 × 10^−9^	− 0.1586	0.1999	0.3311	0.0066	NM_000552 (*VWF*)
Tamoxifen	SERM	***CABIN1***	22q11.23	0.6510	3.26 × 10^−9^	− 0.0575	0.6437	− 0.2398	0.0525	NM_012295 (*CABIN1*)
SNS-314	Aurora kinase	***TPM3***	1q21.3	0.6507	3.34 × 10^−9^	0.1638	0.1854	0.0760	0.5441	NM_152263 (*TPM3*)
ENMD-2076	Aurora kinase	***FOXN3***	14q31.3	− 0.6500	3.52 × 10^−9^	0.0638	0.6081	− 0.0538	0.6677	NM_001085471 (*FOXN3*)
Depsipeptide	HDAC	***FYN***	6q21	− 0.6490	3.78 × 10^−9^	− 0.1180	0.3415	− 0.0357	0.7762	NM_002037 (*FYN*)
XL-888	HSP-90	***ETV6, RNU6-19P***	12p13.2	− 0.6474	4.25 × 10^−9^	0.2387	0.0518	− 0.2837	0.0210	NM_001987 (*ETV6*)
Depsipeptide	HDAC	***MTERFD3***	12q23.3	− 0.6468	4.43 × 10^−9^	0.1686	0.1725	− 0.3133	0.0104	NM_001033050 (*MTERFD3*)
ARQ-197	*c*-Met	***TRIM2***	4q31.3	0.6498	4.73 × 10^−9^	− 0.0801	0.5228	− 0.1558	0.2117	NM_015271 (*TRIM2*)
TAK-901	Aurora kinase	***TREX1***	3p21.31	− 0.6457	4.81 × 10^−9^					
Flavopiridol	CDK	***ATP13A3***	3q29	− 0.6434	5.68 × 10^−9^	0.1702	0.1685	− 0.4160	0.0005	NM_024524 (*ATP13A3*)
YK-4-279	ERG	***TRIM2***	4q31.3	0.6426	6.00 × 10^−9^	− 0.1094	0.3782	− 0.1558	0.2117	NM_015271 (*TRIM2* )
R-547	CDK	***GIGYF2***	2q37.1	0.6424	6.09 × 10^−9^	− 0.1255	0.3114	− 0.1350	0.2798	NM_001103147 (*GIGYF2*)
R-547	CDK	***SP2***	17q21.32	0.6407	6.88 × 10^−9^	0.2484	0.0427	0.1529	0.2203	NM_003110 (*SP2*)
ZIP-301		***TRIM2***	4q31.3	0.6406	6.93 × 10^−9^	− 0.0669	0.5905	− 0.1558	0.2117	NM_015271 (*TRIM2*)
TAK-960 analog	PLK-1	***FGF13***	Xq26.3	0.6396	7.42 × 10^−9^	− 0.0133	0.9151	0.3407	0.0051	NM_004114 (*FGF13*)
Digoxin		***SLC16A6, ARSG***	17q24.2	0.6389	7.83 × 10^−9^	− 0.1918	0.1199	− 0.3055	0.0126	NM_014960 (*ARSG*)
SC-1	Ras-GAP	***WT1***	11p13	− 0.6377	8.50 × 10^−9^	− 0.3245	0.0074	0.5277	5.28 × 10^-6^	NM_024424 (*WT1*)
Depsipeptide	HDAC	***AHSG***	3q27.3	− 0.6374	8.66 × 10^−9^	− 0.0101	0.9356	0.0429	0.7323	NM_001622 (*AHSG*)
ONX-0912	Proteasome	***HRNBP3***	17q25.3	− 0.6368	9.06 × 10^−9^					
GSK-461364	PLK-1	***NRP1***	10p11.22	0.6367	9.09 × 10^−9^	0.2571	0.0357	0.3968	0.0010	NM_003873 (*NRP1*)
SB-590885	BRAF	***LOH12CR1***	12p13.2	− 0.6364	9.31 × 10^−9^	− 0.1878	0.1280	− 0.0711	0.5705	NM_058169 (*LOH12CR1*)
Flavopiridol	CDK	***CYP19A1***	15q21.2	0.6360	9.54 × 10^−9^	0.1601	0.1955	0.1494	0.2313	NM_031226 (*CYP19A1*)

Shown are the strongest correlations with drug response, for genes or combined gene regions with at least one probe satisfying *p*_O_ < 10^−8^ for Spearman correlation between methylation beta-values and log(IC50). Among multiple transcripts matching the same gene, the best matching transcript was identified by finding all Affymetrix transcripts with matching gene names and by selecting the most significant transcript from Spearman correlation between log_2_ of transcript expression and methylation of each probe within the gene/gene region that had been associated with log(IC50) of that agent with *p*_O_ < 5 × 10^−7^. We also provide the NCBI or Ensembl ID of the transcript with a matching gene name that had the most significant Pearson correlation between log2 of transcript expression with log(IC50) of the agent. For those gene regions where probes with *p*_O_ < 5 × 10^−7^ were assigned to multiple genes in the Infinium MethylationEPIC BeadChip manifest annotation [29] according to the UCSC genome annotation, the output summary for multiple genes was combined, as shown in the Gene Regions column for the regions combining *ETV6* and *RNU6-19P* output and *SLC16A6* and *ARSG* output. An expanded list of genes and combined genome regions containing multiple genes, each with at least one methylation probe satisfying *p*_O_ < 9.42 × 10^−8^, is provided in Supplementary Table 4. Correlation results of log_2_ of expression of the most strongly correlated transcript with probe methylation and with log(IC50) are provided for those genes that had matching transcript data in the Affymetrix GeneChip®Human Exon 1.0 ST Array.

*Cluster ID* transcript cluster ID for the Affymetrix GeneChip®Human Exon 1.0 ST Array, *ρ* Spearman correlation coefficient, *r* Pearson correlation coefficient, *CDK* cyclin-dependent kinase, *HDAC* histone deacetylase, *LPAAT-β* lysophosphatidic acid acyltransferase-β, *PLK-1* polo-like kinase 1, *SERM* selective estrogen receptor modulator

**Table 2:** Gene regions with FDR-adjusted *p* value for Spearman correlations of average gene region methylation with log(IC50) of antitumor agents < 0.1

Agent	Inhibitor category	Associated gene	Gene region	Cytoband	Number of probes in gene region	***ρ*** for average region methylation vs log(IC50)	***p***_**FDR**_ for average region methylation vs log(IC50)	Highest |***r|*** for expression vs log(IC50)	Lowest ***p*** for expression vs log(IC50)	Highest |***ρ***| for expression vs region methylation	Lowest ***p*** for expression vs average region methylation	Transcript with lowest ***p*** for expression vs log(IC50)
ABT-348	Aurora kinase	***CEP350***	UTR3	1q25.2	1	0.6096	0.0953	0.0270	0.8281	− 0.1887	0.1292	NM_014810 (*CEP350*)
ABT-737	Bcl-2	***TCERG1L***	UTR3	10q26.3	6	− 0.6251	0.0787	− 0.5740	3.81 × 10^−7^	0.6026	8.64 × 10^−8^	NM_174937 (*TCERG1*L)
AZD-1152	Aurora kinase	***MLPH***	UTR3	2q37.3	1	0.6148	0.0905	0.3014	0.0132	0.5396	2.94 × 10^-6^	NM_024101 *(MLPH)*
BAL-101553	Tubulin fragmenter	***STARD3***	UTR3	17q12	5	0.6154	0.0905	0.0754	0.5440	− 0.0173	0.8900	NM_006804 (*STARD3*)
BI-2536	PLK-1	***MLPH***	UTR3	2q37.3	1	0.6172	0.0905	0.2486	0.0425	0.5396	2.94 × 10^-6^	NM_024101 *(MLPH)*
BIA	GSK-3	***TTC15***	TSS200	2p25.3	4	0.6138	0.0905	− 0.1191	0.3372	− 0.1668	0.1807	NM_016030 (*TTC15*)
BIM-46187	Pan-G-protein	***C8orf74***	UTR5	8p23.1	1	− 0.6721	0.0145*					
BIM-46187	Pan-G-protein	***C8orf74***	Exon 1	8p23.1	1	− 0.6721	0.0145*					
BIM-46187	Pan-G-protein	***C8orf74***	TSS200	8p23.1	3	− 0.6459	0.0530					
Depsipeptide	HDAC	***MTERFD3***	Gene body	12q23.3	1	− 0.6468	0.0530	0.1686	0.1725	− 0.3133	0.0104	NM_001033050 (*MTERFD3*)
Flavopiridol	CDK	***SEC11C***	TSS1500	18q21.32	4	0.6180	0.0905	0.0731	0.5566	0.5476	1.95 × 10^-6^	NM_033280 (*SEC11C*)
GSK-461364	PLK-1	***LOC100128568***	Gene body	19p13.3	20	0.6267	0.0787					
Sapanisertib	mTOR	***SPG20***	UTR5	13q13.3	28	0.6252	0.0787	− 0.3122	0.0101	− 0.5423	2.55 × 10^−6^	NM_001142295 (*SPG20*)
Ivacaftor		***MIF***	TSS200	22q11.23	6	0.6144	0.0905	− 0.2685	0.0280	− 0.4318	0.0003	NM_002415 (*MIF*)
Ivacaftor		***RPUSD2***	Exon 1	15q15.1	4	0.6127	0.0913	− 0.0729	0.5575	− 0.0908	0.4683	NM_152260 (*RPUSD2*)
ONX-0912	Proteasome	***CYP11B2***	Gene body	8q24.3	1	− 0.6078	0.0992	− 0.1414	0.2536	0.1705	0.1711	NM_000498 (*CYP11B2*)
R-547	CDK	***GIGYF2***	TSS1500	2q37.1	4	0.6354	0.0639	− 0.1255	0.3114	− 0.1525	0.2215	NM_001103147 (*GIGYF2*)
R-547	CDK	***KCNH6***	UTR5	17q23.3	4	0.6409	0.0607	0.0677	0.5861	0.1664	0.1818	NM_030779 (*KCNH6*)
R-547	CDK	***TREX1***	Exon 1	3p21.31	9	− 0.6119	0.0913					
R-547	CDK	***TREX1***	UTR5	3p21.31	6	− 0.6109	0.0913					
SC-1	Ras-GAP	***WT1***	UTR3	11p13	1	− 0.6372	0.0639	− 0.3245	0.0074	0.4004	0.0009	NM_024424 (*WT1*)
SCH-1473759	Aurora kinase	***ITPA***	UTR3	20p13	1	− 0.6110	0.0913	0.0138	0.9115	− 0.0562	0.6537	NM_033453 (*ITPA*)
SNS-314	Aurora kinase	***CEP350***	UTR3	1q25.2	1	0.6280	0.0787	− 0.0704	0.5713	− 0.1887	0.1292	NM_014810 (*CEP350*)
TAK-901	Aurora kinase	***TREX1***	Exon 1	3p21.31	9	− 0.6231	0.0796					
TAK-901	Aurora kinase	***TREX1***	UTR5	3p21.31	6	− 0.6088	0.0969					
TAK-960 analog	PLK-1	***GLYATL1***	TSS1500	11q12.1	2	0.6164	0.0905	0.1228	0.3223	0.4304	0.0003	NM_080661 (*GLYATL1*)
Vertex ATR inhibitor Cpd 45	ATR	***TREX1***	Exon 1	3p21.31	9	− 0.6244	0.0787					
Vertex ATR inhibitor Cpd 45	ATR	***TREX1***	UTR5	3p21.31	6	− 0.6182	0.0905					

Correlation results of log_2_ of expression of the most strongly correlated transcript with gene region methylation and with log(IC50) are provided for those genes that had matching transcript data in the Affymetrix GeneChip®Human Exon 1.0 ST Array. Number of probes in gene region provides the number of probes annotated by the Illumina Infinium MethylationEPIC BeadChip manifest annotation [18] according to the UCSC genome browser as belonging to a particular gene region; methylation beta-values of all such probes were combined to compute the average region methylation value. *p*FDR *p* value adjusted for false discovery rate, accounting for all 412 drug agents with variable drug response and 108,795 gene regions

*p_FDR_ < 0.05

*ρ* Spearman correlation coefficient, *r* Pearson correlation coefficient, *ATR* ataxia telangiectasia and Rad3-related protein, *CDK* cyclin-dependent kinase, *GSK-3* glycogen synthase kinase 3, *PLK-1* polo-like kinase 1

The strongest probe correlation satisfied the Bonferroni-adjusted threshold for multiple testing of EPIC array probes with 412 agents (*p*_O_ < 2.29 × 10^−10^). It involved the probe cg13178916 in the body of *SNED1* and resistance to the histone deacetylase (HDAC) inhibitor 4SC-202 (*ρ* = 0.6927, *p*_O_ = 1.16 × 10^−10^; Table 1). Due to similar mechanisms of action of various agents, their associations with DNA methylation are likely not independent, and the Bonferroni threshold is likely to be excessively stringent. When using *p*_O_ < 9.42 × 10^−8^, the probe cg13178916, which had the range of beta-values from 0.096 to 0.843, was also associated with microtubule-disruptive agents BAL-101553 and vinblastine (Supplementary Tables 3 and 4; Supplementary Data 1). Even though other *SNED1* probes did not satisfy the significance threshold for multiple testing, 52 probes were associated with resistance to 4SC-202 with *p*_O_*<* 0.05, including 44 probes with *p*_O_*<* 0.01 (*ρ* ≥ 0.3197; Supplementary Data 2A). The probes cg10717312 and cg07644939, located immediately adjacent to cg13178916, were among the 7 probes most strongly associated with 4SC-202 (*p*_O_*<* 5 × 10^−5^, *ρ* ≥ 0.4935; Supplementary Data 1 and 2A). SNED1, a Sushi, Nidogen, and EGF-like Domain 1 extracellular matrix protein, is associated with progression and metastasis of mammary carcinomas and with poor outcomes in ER^−^/PR^−^ breast cancer [49]. Deletion of the chromosomal region 2q37.3 containing *SNED1* is a recurring event in cancer, and in ovarian cancer cell lines, it was associated with resistance to the HDAC inhibitor vorinostat [47]. Similar to 4SC-202, multiple *SNED1* probes were weakly associated with resistance to vorinostat (Supplementary Data 2B). Methylation of cg13178916 was weakly positively associated with transcript expression (Spearman *ρ* = 0.2178; Table 1; Supplementary Table 4). Expression of the *SNED1* transcript NM_001080437 (Affymetrix cluster ID 2536071) was weakly but significantly associated with multiple HDAC inhibitors, although its association with resistance 4SC-202 and vorinostat was weak and did not reach statistical significance (Supplementary Data 2C). The number of cell lines with *SNED1* deletion in our data was insufficient to derive any conclusions about its association with drug response or DNA methylation (Supplementary Data 2D).

Correlation of the probe cg00870242 in *C8orf74*, which encodes an uncharacterized protein, with BIM-46187, an inhibitor of heterotrimeric G-protein signaling, was the second strongest among probes (*p*_O_ = 6.47 × 10^−10^, *ρ* = − 0.6721; Table 1; Supplementary Tables 2, 3 and 4). In total, 4 probes in *C8orf74* were associated with response to BIM-46187 with *p*_O_ < 9.42 × 10^−8^ (Supplementary Table 3; Supplementary Data 2D). *C8orf74* regions were also significantly (*p*_FDR_ = 0.0145 for the 5′ UTR and first exon) or nearly significantly (*p*_FDR_ = 0.053 for TSS200) associated with response to BIM-46187 (*ρ* < − 0.645, Table 2).

**Fig. 1:**
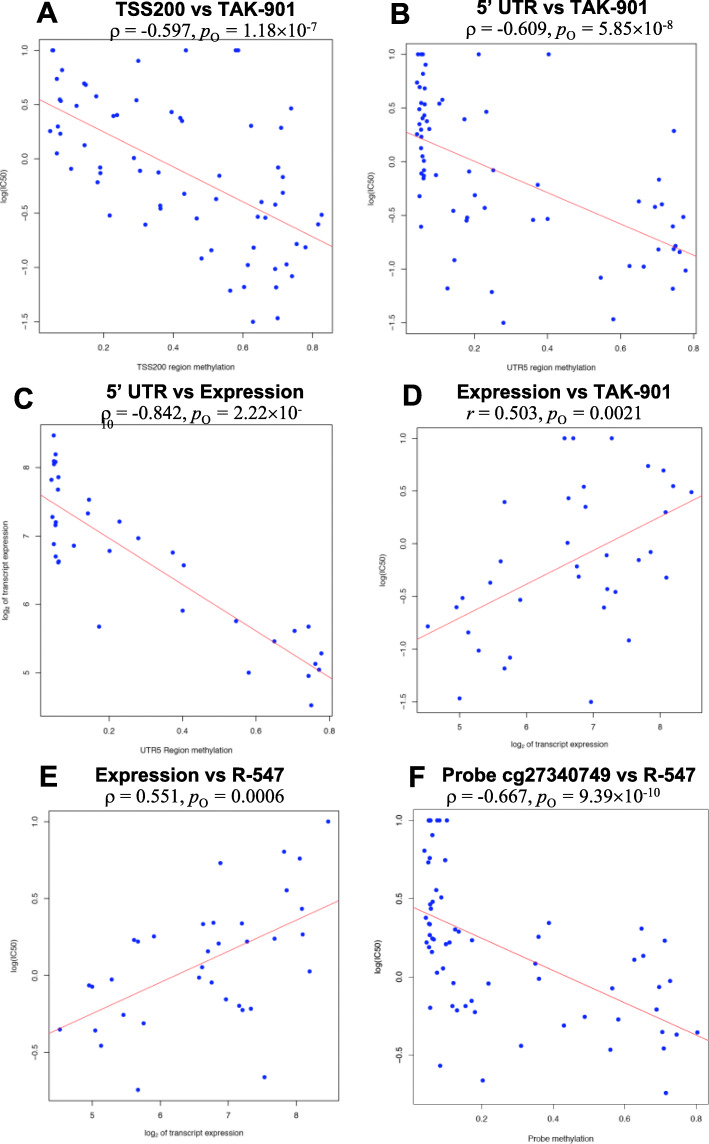
Scatterplots of *TREX1* DNA methylation, transcript expression, and measures of SCLC drug sensitivity. **a** Methylation of the TSS200 region vs log(IC50) of TAK-901. Horizontal scale represents the average methylation beta-value among the probes in the *TREX1* TSS200 region, whereas the vertical scale represents the log(IC50) values of TAK-901. **b** Methylation of the 5′UTR region vs log(IC50) of TAK-901. Horizontal scale represents the average methylation of the *TREX1* 5′UTR region, and the vertical scale represents the log(IC50) values of TAK-901. **c** Methylation of the 5′UTR region of *TREX1* vs *TREX1* expression. Horizontal scale represents the average methylation of the *TREX1* 5′UTR region, whereas the vertical scale represents the log_2_-transformed *TREX1* expression measures. **d***TREX1* expression vs log(IC50) of TAK-901. Horizontal scale represents the log_2_-transformed *TREX1* expression values, and the vertical scale represents the log(IC50) values of TAK-901. **e***TREX1* expression vs log(IC50) of R-547. Horizontal scale represents the log_2_-transformed *TREX1* expression values, and the vertical scale represents the log(IC50) values of R-547. **f** Methylation of the probe cg27340749 vs log(IC50) of R-547. Horizontal scale represents the average methylation of the cg27340749, which is jointly annotated as being the 5′UTR and first exon of *TREX1.* The vertical scale represents the log(IC50) values of R-547. *ρ*, Spearman correlation coefficient. *r*, Pearson correlation coefficient. The original *p* values (*p*_O_) are provided for respective Spearman and Pearson correlation analyses

**Fig. 2:**
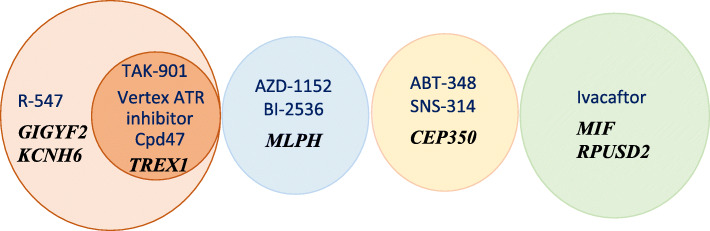
Venn diagram presenting agents from Table 2 associated with gene regions of multiple genes and/or genes from Table 2, regions of which were associated with multiple agents with *p*_FDR_ < 0.1. Drug names are provided in blue font, whereas the names of associated genes are provided in italic black font. Circle sizes are proportionate to the number of associated genes. Overlapping regions show genes associated with multiple agents or agents associated with multiple genes. The full list of gene region-agent associations with *p*_FDR_ < 0.1 including additional single gene-single agent associations is provided in Table 2

Correlation of *TREX1* methylation with sensitivity to R-547 was the third strongest association among probes (*p*_O_ = 9.39 × 10^−10^, *ρ* < − 0.6674; Table 1; Fig. 1; Supplementary Tables 3 and 4; Supplementary Data 3 and 4). *TREX1*, which encodes the 3′ exonuclease I (DNase III), is upregulated after treatment of malignant cells with several categories of DNA damaging agents or after UV light exposure [50–52]. TREX1 has been associated with cancer cell sensitivity to DNA-damaging agents and with DNA repair or DNA degradation in apoptotic cells after drug exposure [50–52]. Using *p*_O_ < 9.42 × 10^−8^, multiple *TREX1* probes were associated with the CDK inhibitor R-547; the Aurora kinase inhibitors AZD-1152, SCH-1473759, SNS-314, and TAK-901; the Vertex ATR inhibitor Cpd 45, which affects the DNA damage response pathway; and vinorelbine which disrupts the mitotic spindle (Table 1; Supplementary Tables 3 and 4). Increased methylation of *TREX1* regions was negatively associated with sensitivity to R-547, TAK-901, and the Vertex ATR inhibitor Cpd 45 (*p*_FDR_ < 0.1; Table 2; Figs. 1 and 2). Methylation of the first exon of *TREX1* was associated with response to digoxin, the kinesin spindle protein (KSP) inhibitor ARRY-520 (isomer B), and the KSP/Eg5 inhibitor ARQ-621 when using a less stringent threshold of *p*_FDR_ < 0.15 (− 0.5999≤ *ρ* ≤ − 0.5811, 1.02 × 10^−7^ ≤ *p*_O_ ≤ 3.12 × 10^−7^, 0.1112 ≤ *p*_FDR_ ≤ 0.1396; Supplementary Table 5). Methylation of the upstream regions and gene body was strongly negatively associated with *TREX1* expression (Spearman *ρ* = − 0.350, *p*_O_ = 0.0394 for TSS1500; *ρ* = − 0.692, *p*_O_ = 4.14 × 10^−6^ for TSS200; *ρ* = − 0.842, *p*_O_ = 2.23 × 10^−10^ for the 5′UTR; *ρ* = − 0.825, *p*_O_ = 1.07 × 10^−9^ for exon 1; and *ρ* = − 0.779, *p*_O_ = 3.54 × 10^−8^ for the gene body). Methylation of the 3′UTR was not associated with expression (*ρ* = − 0.037, *p*_O_ = 0.8348). As a corollary to the strong negative correlation between methylation of most of the *TREX1* regions and expression, increased *TREX1* expression was strongly associated with resistance to many agents, e.g., digoxin, ARQ-621, SNS-314, R-547, AZD-1152, vinorelbine, SCH-1473759, TAK-901, Vertex ATR inhibitor Cpd 45, and ARRY-520 isomer B (0.440 ≤ *r* ≤ 0.582, 0.0002 ≤ *p*_O_ ≤ 0.0107; several correlations are presented in Fig. 1**)**.

Associations of *TREX1* promoter methylation and expression with log(IC50) of vinorelbine were validated in SCLC cell lines from the CCLE and GDSC datasets (− 0.6506 ≤ Spearman *ρ* ≤ − 0.4275, 0.0006 ≤ *p*_O_ ≤ 0.0207 for significant correlations of various measures of *TREX1* promoter methylation and log(IC50) of vinorelbine from both GDSC1 and GDSC2; Pearson *r* = 0.6245, *p*_O_ = 0.0006 for correlation between *TREX1* expression and vinorelbine response from GDSC2; data not shown). Consistent with association with vinorelbine, *TREX1* promoter methylation and expression were correlated with other antimitotic agents including vinblastine, vincristine, and paclitaxel in SCLC lines from both GDSC1 and GDSC2 datasets (for multiple promoter methylation measures, − 0.7236 ≤ *ρ* ≤ − 0.4196, 0.0001 ≤ *p*_O_ ≤ 0.0262; for expression, 0.3553 ≤ *r* ≤ 0.5186, 0.0061 ≤ *p*_O_ ≤ 0.0362; data not shown).

GDSC included data for the Aurora kinase inhibitors ZM447439, tozasertib, and alisertib (MLN-8237). Alisertib/MLN-8237 was also included in our dataset, and its activity was associated with increased methylation of all *TREX1* regions other than the 3′UTR (*p*_O_ ≤ 0.0231), although they did not reach statistical significance after adjustment for multiple testing at the epigenome-wide level and accounting for all agents. The strongest associations with alisertib sensitivity in our data were observed for TSS200, 5′UTR, first exon, and gene body (− 0.5676 ≤ *ρ* ≤ − 0.4995, 6.70 × 10^−7^ ≤ *p*_O_ ≤ 1.96 × 10^− 5^; data not shown). Methylation of 11 *TREX1* probes (out of 19 total; Supplementary Data 3) in our data was also associated with sensitivity to MLN-8237 but did not reach significance after adjustment for multiple testing (− 0.5837 ≤ *ρ* ≤ − 0.4235, 2.70 × 10^−7^ ≤ *p*_O_ ≤ 0.0004; data not shown). Consistent with our data, several *TREX1* promoter methylation measures and *TREX1* expression were associated with alisertib sensitivity in GDSC2 (e.g., − 0.4996 ≤ *ρ* ≤ − 0.4292, 0.0178 ≤ *p*_O_ ≤ 0.0590 for promoter CpG island methylation; *r* = 0.4365, *p*_O_ = 0.0258 for expression). In GDSC1, *TREX1* promoter methylation was associated with sensitivity to tozasertib and ZM447439 (− 0.5739 ≤ *ρ* ≤ − 0.4468, 0.0007 ≤ *p*_O_ ≤ 0.0326 for the methylation measures most strongly associated with both agents), and expression was associated with sensitivity to tozasertib (*r* = 0.5805, *p*_O_ = 0.0023). These correlations in multiple datasets suggest that *TREX1* methylation and expression are associated with SCLC response to Aurora kinase inhibitors, antimitotic agents, and a number of additional drug categories.

Supplementary Table 4 provides a gene level summary for 182 gene-drug correlations for those genes in our dataset that had one or more probes with *p*_O_ < 9.42 × 10^−8^. It also summarizes matching transcript correlation with methylation probes in or near these genes that satisfied a less stringent criterion of *p*_O_ < 5 × 10^−7^. Spearman correlations of 1180 methylation probes satisfied *p*_O_ < 5 × 10^−7^, with |*ρ*| > 0.57, and 519 gene-drug correlations included genes with probes with *p*_O_ between 9.42 × 10^−8^ and 5 × 10^−7^ (data not shown). Some of these less significant results may be of biological interest. For example, methylation of the probe cg17159843 in the TSS200 of *ERBB2* was associated with resistance to the BET bromodomain inhibitor, (+)-JQ1 (*ρ* = 0.5757, *p*_O_ = 4.25 × 10^−7^; data not shown). Methylation of cg17159843 was negatively correlated with expression of the *ERBB2* transcript NM_001005862 (*ρ* = − 0.4963, *p* = 2.25 × 10^−5^), and increased *ERBB2* expression was associated with sensitivity to JQ1 (Pearson *r* = −0.3112, *p* = 0.0104). TSS200 and the first exon of *ERBB2* were also strongly associated with log(IC50) of JQ1 (*ρ* = 0.5195 and 0.5345, *p*_O_ = 3.79 × 10^−6^ and 7.84 × 10^−6^, respectively), however, not statistically significant after adjustment for multiple testing of all 108,795 gene regions and 412 agents (*p*_FDR_ ≥ 0.23). Upregulated expression of *ERBB2* was previously associated with acquired multidrug resistance in SCLC [53, 54]. HER2-positive (HER2^+^) breast cancer cells were sensitive to JQ1, and BET bromodomain inhibitors may alleviate acquired resistance of HER2^+^ breast cancer cells to lapatinib [55]. Combinations of BET inhibitors with other agents in SCLC with increased *ERBB2* expression may be a potential way to overcome drug resistance. Indeed, addition of the BET inhibitor MK-8628 increased the killing of SCLC lines in combination with etoposide or topotecan in a study of triple drug combinations [38].

**Fig. 3:**
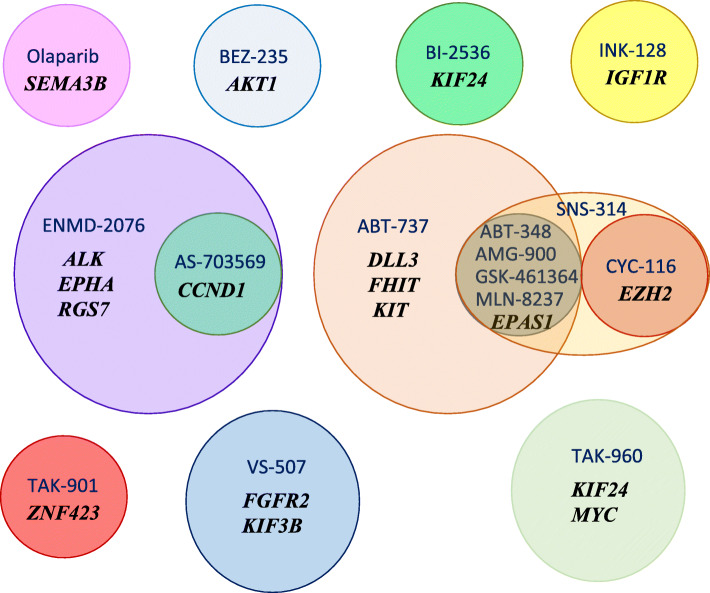
Venn diagram presenting candidate gene-drug associations from Table 3 based on individual probes, with *p*_FDR_ < 0.1. Drug names are provided in blue font, whereas the names of associated genes are provided in italic black font. The size of the circles is proportionate to the number of associated genes. Overlapping regions show genes associated with multiple agents or agents associated with multiple genes

Table 2 provides the list of gene regions associated with log(IC50) with *p*_FDR_ < 0.1 at the epigenome-wide level (with Spearman |*ρ*| > 0.607). Figure 2 provides a Venn diagram of the subset of these associations with *p*_FDR_ < 0.1 that involved multiple agents per gene or multiple genes per agent. An expanded list of regions that satisfied a less stringent criterion of *p*_FDR_ < 0.15 at the epigenome-wide level (with |*ρ*| ≥ 0.579) is provided in Supplementary Table 5. As discussed above, *TREX1* regions were associated with multiple agents. The 3′UTR of *MLPH* and *CEP350* was also associated with multiple agents with *p*_FDR_ < 0.1 (Fig. 3; Supplementary Figure 1). The 3′UTR of *MLPH* was associated with the Aurora kinase inhibitor AZD-1152 and the polo-like kinase (PLK) inhibitor BI-2536. The 3′UTR of *CEP350* was associated with ABT-348, which inhibits multiple kinases including Aurora kinases [56], and the Aurora kinase inhibitor SNS-314 (*ρ* ≥ 0.610). At *p*_FDR_ < 0.15, the 3′UTR of both *MLPH* and *CEP350* was associated with the PLK-1 inhibitor TAK-960, *MLPH* was associated with the Aurora kinase inhibitor SCH-1473759, and CEP350 with a TAK-960 analog (*ρ* ≥ 0.587; Supplementary Table 5; Supplementary Figure 1). Association of *TREX1*, *MLPH*, and *CEP350* methylation with multiple Aurora kinase inhibitors is notable, as Aurora kinase inhibitors were highly effective against SCLC cell lines and induced a partial response in SCLC patients in a clinical study [24, 57]. Associations with *MLPH* and *CEP350* may be based on functional interactions, as regulatory roles of Aurora kinases include mitotic regulation of microtubule interactions, mitotic spindle assembly, and centrosome maturation [58, 59].

MLPH, melanophilin, has a role as a microtubule plus-end tracking protein and interacts with microtubule plus end-binding protein EB1, which is involved in spindle symmetry in mitosis [60–62]. CEP350, centrosomal protein 350, is a putative substrate of Aurora kinases as a part of centrosomal maturation machinery [59]. While the methylation of the 3′UTR regions of *MLPH* and *CEP350* was strongly associated with drug response, its biological consequences require further investigation, as each region was represented by a single probe. Methylation of the 3′UTR of *MLPH* was strongly positively associated with *MLPH* expression (*ρ* = 0.5396, *p*_O_ = 2.94 × 10^−6^). The 3′UTR of *CEP350* was very weakly negatively associated with its expression (*ρ* = − 0.1887, *p*_O_ = 0.1227; Supplementary Table 5).

### Association of methylation of probes and regions in candidate genes with drug response

Analysis of the association of methylation probes and regions in candidate genes with 44 antitumor agents listed in Supplementary Table 2 revealed 29 probe-drug associations involving 18 genes and 16 agents with *p*_FDR_*<* 0.1 (Spearman |*ρ*| > 0.516|). The summary of these associations at the gene level, representing 24 gene-drug associations, is provided in Table 3 and Fig. 3. Supplementary Tables 6 and 7 provide expanded lists of gene-drug and probe-drug associations involving probes with *p*_FDR_*<* 0.15 (|*ρ*| ≥ 0.481).

**Table 3:** Candidate genes with methylation probes that had correlations with log(IC50) of candidate antitumor agents satisfying *p*_FDR_ < 0.1

Agent	Inhibitor category	Associated gene	Location	Highest |***ρ***| for probe methylation vs log(IC50)	Lowest ***p***_**FDR**_ for probe methylation vs log(IC50)	Highest |***r***| for transcript expression vs log(IC50)	Lowest ***p*** for transcript expression vs logIC50	Highest |***ρ***| for transcript expression vs probe methylation	Lowest ***p*** for transcript expression vs probe methylation	Transcript with lowest ***p*** for expression vs log(IC50)
ABT-348	Aurora kinase	*EPAS1*	2p21	0.5548	0.0515	0.3638	0.0025	0.6800	3.39 × 10^−10^	NM_001430 (*EPAS1*)
ABT-737	Bcl-2	*DLL3*	19q13.2	0.5506	0.0515	− 0.4139	0.0005	− 0.4426	0.0002	NM_016941 (*DLL3*)
ABT-737	Bcl-2	*FHIT*	3p14.2	− 0.5502	0.0515	0.1830	0.1382	− 0.3164	0.0097	ENST00000468189 (*FHIT*)
ABT-737	Bcl-2	*KIT*	4q12	− 0.5391	0.0580	− 0.5372	2.77 × 10^−6^	0.5426	2.52 × 10^−6^	NM_000222 (*KIT*)
ABT-737	Bcl-2	*EPAS1*	2p21	− 0.5204	0.0937	− 0.0501	0.6873	0.2638	0.0324	NM_001430 (*EPAS1*)
AMG-900	Aurora kinase	*EPAS1*	2p21	0.6278	0.0052*	0.3966	0.0009	0.6800	3.39 × 10^−10^	NM_001430 (*EPAS1*)
AS-703569	Aurora kinase	*CCND1*	11q13.3	− 0.5568	0.0515	− 0.3614	0.0027	0.5972	1.20 × 10^−7^	NM_053056 (*CCND1*)
BEZ-235	mTOR	*AKT1*	14q32.33	− 0.5370	0.0604	0.2562	0.0363	− 0.3499	0.0040	NM_001014432 (*AKT1*)
BI-2536	PLK-1	*KIF24*	9p13.3	0.5403	0.0580	0.1740	0.1591	0.0689	0.5828	NM_194313 (*KIF24*)
CYC-116	Aurora kinase	*EZH2*	7q36.1	0.5456	0.0515	− 0.0162	0.8966	− 0.1156	0.3554	NM_004456 (*EZH2*)
ENMD-2076	Aurora kinase	*CCND1*	11q13.3	− 0.5777	0.0391*	− 0.4201	0.0004	0.5972	1.20 × 10^−7^	NM_053056 (*CCND1*)
ENMD-2076	Aurora kinase	*ALK*	2p23.2	− 0.5551	0.0515	0.0036	0.9768	0.2331	0.0597	NM_004304 (*ALK*)
ENMD-2076	Aurora kinase	*EPHA2*	1p36.13	0.5233	0.0876	− 0.2090	0.0897	− 0.3787	0.0017	NM_004431 (*EPHA2*)
ENMD-2076	Aurora kinase	*RGS7*	1q43	0.5191	0.0948	0.4887	2.72 × 10^−5^	0.7412	1.11 × 10^−12^	NM_002924 (*RGS7*)
GSK-461364	PLK-1	*EPAS1*	2p21	0.5165	0.0994	0.3554	0.0032	0.6800	3.39 × 10^−10^	NM_001430 (*EPAS1*)
Sapanisertib	mTOR	*IGF1R*	15q26.3	0.5262	0.0798	− 0.0889	0.4744	− 0.1369	0.2730	NM_000875 (*IGF1R*)
MLN-8237	Aurora kinase	*EPAS1*	2p21	0.5462	0.0515	0.3245	0.0074	0.6800	3.39 × 10^−10^	NM_001430 (*EPAS1*)
Olaparib	PARP1	*SEMA3B*	3p21.31	0.5264	0.0798	− 0.0815	0.5120	0.0907	0.4687	NM_004636 (*SEMA3B*)
SNS-314	Aurora kinase	*EPAS1*	2p21	0.5880	0.0322*	0.3434	0.0044	0.6800	3.39 × 10^−10^	NM_001430 (*EPAS1*)
SNS-314	Aurora kinase	*EZH2*	7q36.1	0.5464	0.0515	− 0.0444	0.7215	− 0.1156	0.3554	NM_004456 (*EZH2*)
TAK-901	Aurora kinase	*ZNF423*	16q12.1	− 0.5360	0.0604	− 0.1859	0.1319	0.5773	3.88 × 10^−7^	NM_015069 (*ZNF423*)
TAK-960	PLK-1	*KIF24*	9p13.3	0.5571	0.0515	0.2285	0.0630	0.0689	0.5828	NM_194313 (*KIF24*)
TAK-960	PLK-1	*MYC*	8q24.21	0.5432	0.0540	− 0.1537	0.2143	0.2652	0.0314	NM_002467 (*MYC*)
Salinomycin	Apoptotic	*FGFR2*	10q26.13	− 0.5334	0.0650	− 0.3237	0.0075	0.6785	3.84 × 10^−10^	NM_000141 (*FGFR2*)
Salinomycin	Apoptotic	*KIF3B*	20q11.21	− 0.5202	0.0937	0.1938	0.1162	− 0.4576	0.0001	NM_004798 (*KIF3B*)

Listed are the gene-drug correlations for candidate genes in which one or more probes had *p*_FDR_ < 0.1 for Spearman correlation with agents listed in Supplementary Table 2. Supplementary Table 6 provides the list of candidate gene-drug pairs satisfying a less stringent criterion of *p*_FDR_ < 0.15. The list of individual probes and their individual *p* values are provided in Supplementary Table 7

*p*_FDR_, FDR-adjusted *p* value, accounting for multiple testing of 10,515 methylation probes in or near candidate genes and 44 agents listed in Supplementary Table 2.

*p_FDR_ < 0.05

*ρ* Spearman correlation coefficient, *r* Pearson correlation coefficient

Associations of the probe cg08937075 in *EPAS1*, which encodes the hypoxia-inducible factor 2α (HIF2α), with the Aurora kinase inhibitors AMG-900 and SNS-314 and of cg09637363 in *CCND1* with the Aurora A kinase/tyrosine kinase inhibitor ENMD-2076 had *p*_FDR_ < 0.05 (Table 3; Supplementary Table 7). Multiple other probes in both genes had strong correlations with these agents (Supplementary Table 7). The probe cg08937075 in the *EPAS1* body was significantly associated with response to AMG-900 not only when testing among candidate genes, but also at the epigenome-wide level among all Illumina EPIC array probes (*p*_O_ = 1.68 *×* 10^−8^, *ρ* = 0.6278; Supplementary Table 3; Supplementary Figure 2). In total, 13 probes in the body of *EPAS1*, upstream of that gene, or in the 3′UTR had modest or strong correlations with resistance to AMG-900 (Supplementary Data 2D). Among candidate drug-gene associations, *EPAS1* probes had FDR < 0.1 in associations with six agents (Table 3, Fig. 3), including resistance to Aurora kinase inhibitors AMG-900, SNS-314, and MLN-8237; multiple kinase inhibitor including Aurora kinase ABT-348, and the PLK-1 inhibitor GSK-461364 (0.5165 ≤ *ρ* ≤ 0.6278), and sensitivity to the Bcl-2 inhibitor ABT-737 (*ρ* = − 0.5204). *EPAS1* gene regions were not significantly associated with drug response (the strongest correlation, with etoposide, had *p*_FDR_ = 0.509, *ρ* = 0.4122; data not shown). Methylation of *EPAS1* probes which were associated with drug response was significantly positively correlated with *EPAS1* expression (*ρ* = 0.6800, *p*_O_ = 3.39 *×* 10^−10^ for cg08937075), and both methylation of *EPAS1* probes and *EPAS1* expression had the same direction of associations with drug response (Table 3; Supplementary Figure 2), which suggests that *EPAS1* expression may be regulated by mechanisms other than DNA methylation. While *EPAS1* expression is associated with response to the KSP/Eg5 inhibitor ARQ-621 [24], however, the correlation of *EPAS1* methylation with response to that agent was weak (*ρ* ≤ 0.2147 for different regions), was in same direction as that for gene expression, and did not reach statistical significance. We did not find any association of *EPAS1* copy number with probe methylation, transcript expression, or response to the Aurora kinase inhibitors AMG-900 and SNS-314 (Spearman *ρ* and Pearson *r* < 0.2; data not shown), and the molecular basis for the association between methylation of *EPAS1* probes and drug response remains unclear.

*CCND1*, encoding cyclin D1, is overexpressed in some SCLC tumors [5]. The probes in its upstream region and gene body had the opposite directions of association with drug response and with *CCND1* expression (Supplementary Table 7). The probes cg09637363 and cg19964454 in its body were associated with sensitivity to the Aurora kinase inhibitors ENMD-2076, AS-703569, SCH-1473759, CYC-116, and MLN-8237 (− 0.5777 ≤ *ρ* ≤ − 0.4855, 0.0391 ≤ *p*_FDR_ ≤ 0.1366; Supplementary Table 7). Methylation of both probes cg09637363 and cg19964454 was strongly positively associated (*ρ* = 0.5972 and 0.5617, *p*_O_ = 1.20 × 10^−7^ and 9.24 × 10^−7^, respectively; data not shown) with expression of one of the two *CCND1* transcripts with available data, NM_053056 (Affymetrix cluster ID 3338192). The significance of the positive correlation of the probes in the gene body with expression is unclear, although it is consistent with a report of multiple positive correlations of some gene body probes with expression in the Cancer Genome Atlas (TCGA) data [42, 63]. Expression of the *CCND1* transcript 3338192 was modestly associated with SCLC sensitivity to the same agents which were associated with the probes cg09637363 and cg19964454 (Pearson correlation with transcript expression − 0.4201 ≤ *r* ≤ − 0.3015, 0.0004 ≤ *p* ≤ 0.0131 for ENMD-2076, AS-703569, SCH-1473759, CYC-116, and MLN-8237; data not shown). In contrast to the probes in the body of *CCND1*, the probes cg01406280, cg11190277, and cg19209049 in the TSS1500 were associated with resistance to the Aurora kinase inhibitor ENMD-2076, a dual PI3K/mTOR inhibitor BEZ-235, and the PLK inhibitor BI-2536 (0.4811 ≤ *ρ* ≤ 0.5484, 0.0515 ≤ *p*_FDR_ ≤ 0.1484). Methylation of the TSS1500 probes was negatively associated with expression of the *CCND1* transcript 3338192 (e.g., *ρ* = − 0.3569, *p* = 0.0032 for cg01406280; data not shown). Expression of that transcript was associated with sensitivity to all three agents (− 0.4291 ≤ *r* ≤ − 0.2298, 0.0004 ≤ *p* ≤ 0.0614). Negative association between TSS1500 probes and expression of the transcript 3338192 suggests a possible regulatory role of methylation of the upstream region of *CCND1*.

In contrast, the second *CCND1* transcript (Affymetrix cluster ID 3380065) was not associated with any of the above agents or with methylation of the probes correlated with these agents (Pearson |*r*| and Spearman |*ρ*| ≤ 0.2, *p* ≥ 0.1). Therefore, possible associations between *CCND1* methylation and drug response could be mediated by the transcript 3338192. Analysis of the possible effect of *CCND1* copy number on these associations did not provide conclusive results as only one cell line with available copy number data, DMS 114, had a high-level amplification of *CCND1.*

Analysis of association of regions of candidate genes with 44 agents identified five gene-drug pairs involving four genes which had *p*_FDR_*<* 0.1, with Spearman *ρ* ≥ 0.51 (Table 4). All associations indicated increased drug resistance for higher methylation of the respective gene regions. Two associations, of the TSS1500 of *PTGFRN* with the dual PI3K/mTOR inhibitor BEZ-235 (dactolisib) and of the *KDM1A* body with the PLK-1 inhibitor TAK-960, had *p*_FDR_ < 0.05. Methylation of the regions of three out of four genes (*PTGFRN*, *KDM1A*, and *MDM2*) listed in Table 4 was significantly negatively associated with expression of their transcripts (− 0.4786 ≤ Spearman *ρ* ≤ − 0.2436, 4.81 × 10^−5^ ≤ *p*_O_ ≤ 0.0487). This may indicate a negative regulatory effect of DNA methylation on transcript expression. Accordingly, increased expression of these three genes was associated with sensitivity to the agents listed in Table 4 (− 0.4017 ≤ Pearson *r* ≤ − 0.2325, 0.0008 ≤ *p*_O_ ≤ 0.0752). Supplementary Table 8 provides an expanded list of associations between gene regions and drug response satisfying *p*_FDR_*<* 0.15.

**Table 4:** Correlations in candidate gene regions with log(IC50) of candidate antitumor agents that satisfied *p*_FDR_ < 0.1

Agent	Inhibitor category	Gene	Region	Cytoband	Number of probes in gene region	***ρ*** for average region methylation vs log(IC50)	***p***_**FDR**_ for average region methylation vs log(IC50)	Highest |***r|*** for expression vs log(IC50)	Lowest ***p*** for expression vs log(IC50)	Highest |***ρ***| for expression vs region methylation	Lowest ***p*** for expression vs average region methylation	Transcript with lowest ***p*** for expression vs log(IC50)
ABT-263	Bcl-2	***MDM2***	TSS200	12q15	7	0.5234	0.0617	− 0.4017	0.0008	− 0.4269	0.0004	NM_002392 (*MDM2*)
ABT-737	Bcl-2	***MDM2***	TSS200	12q15	7	0.5287	0.0617	− 0.3867	0.0012	− 0.4269	0.0004	NM_002392 (*MDM2*)
BEZ-235	mTOR	***PTGFRN***	TSS1500	1p13.1	5	0.5565	0.0352*	− 0.2189	0.0752	− 0.4786	4.81 × 10^−5^	NM_020440 (*PTGFRN*)
Sapanisertib	mTOR	***IGFBP5***	TSS1500	2q35	5	0.5107	0.0896	0.2969	0.0147	0.2035	0.1013	NM_000599 (*IGFBP5*)
TAK-960	PLK-1	***KDM1A***	Gene body	1p36.12	11	0.5486	0.0352*	− 0.2325	0.0583	− 0.2436	0.0487	NM_001009999 (*KDM1A*)

Number of probes in gene region provides the number of probes annotated by the Illumina Infinium MethylationEPIC BeadChip manifest annotation [18] according to the UCSC genome browser as belonging to a particular gene region; methylation beta-values of all such probes were combined to compute the average region methylation value. Correlation results of log_2_ of expression of the most strongly correlated transcript with gene region methylation and with log(IC50) are provided for those genes. *p*_FDR_, *p* value adjusted for false discovery rate, accounting for multiple testing of 1376 candidate gene regions and 44 agents listed in Supplementary Table 2.

*p_FDR_ < 0.05

*ρ* Spearman correlation coefficient, *r* Pearson correlation coefficient

Methylation of the body of *KDM1A*, which encodes lysine demethylase 1A (LSD1), an epigenetic histone modifier, was significantly associated with resistance to the PLK-1 inhibitor TAK-960 (Spearman *ρ* = 0.5486, *p*_FDR_ = 0.0352; Table 4). Using a less stringent threshold (*p*_FDR_ < 0.15), it was also associated with resistance to another PLK-1 inhibitor GSK-461364, and to the KSP inhibitor SB-743921 (*ρ* = 0.4619 and 0.4722, respectively; *p*_FDR_ = 0.1347 for both agents, Supplementary Table 8). Methylation of the *KDM1A* body was significantly negatively correlated with expression of its transcript with Affymetrix cluster ID 2325002 (NCBI locus ID NM_001009999; Spearman *ρ* = − 0.2436, *p* = 0.0487; Table 4), and increased *KDM1A* transcript expression was weakly correlated with sensitivity to TAK-960 (Pearson *r* = − 0.2325, *p* = 0.0583; Table 4). LSD1 is overexpressed in SCLC and is the target of selective LSD1 inhibitors which are currently being pursued in the clinical setting [2, 3]. LSD1 directly regulates the transcription of *PLK-1* [64], which could suggest a potential molecular mechanism of association between increased methylation and reduced expression of *KDM1A* and resistance to PLK-1 inhibitors.

All other gene region associations with *p*_FDR_ < 0.1 involved upstream regions. Methylation of the TSS1500 of *PTGFRN* was significantly associated with resistance to the dual PI3K/mTOR inhibitor BEZ-235 (*ρ* = 0.5565, *p*_FDR_ = 0.0352; Table 4). *PTGFRN*, a frequently mutated gene in SCLC, encodes the prostaglandin receptor F2 inhibitor that inhibits the binding of prostaglandin F2α to its receptor [5]. In agreement with the correlation of the TSS1500 region, the probe cg08361238 in that region was also associated with BEZ-235 (*ρ* = 0.5040, *p*_FDR_ = 0.1184; Supplementary Table 7). Methylation of the TSS1500 of *PTGFRN* was significantly negatively correlated with expression of the *PTGFRN* transcript NM_020440 (cluster ID 2353717; *ρ* = − 0.4786, *p* = 4.81 × 10^−5^). Accordingly, increased expression of that transcript was associated with sensitivity to BEZ-235 (Table 4).

Methylation of the TSS200 of *MDM2* was associated with resistance to Bcl-2 inhibitors ABT-263 (Navitoclax) and ABT-737, with *ρ* = 0.52 and *p*_FDR_ = 0.0617 for both agents (Supplementary Table 8). The probe cg04667586 in the TSS200 and the gene body was also associated with log(IC_50_) of ABT-263 (*ρ* = 0.5070, *p*_FDR_ = 0.1184; Supplementary Table 7). TSS200 was significantly negatively associated with expression of the *MDM2* transcript NM_002392 (cluster ID 3421300, *r =* − 0.4269, *p =* 0.0004), which was correlated with sensitivity to both Bcl-2 inhibitors (*ρ* = − 0.4017, *p* = 0.0008 for ABT-263 and *r* = − 0.3867, *p =* 0.0012 for ABT-737). While MDM2 inhibitors alone were not effective in the in vitro screens of SCLC cell lines [24, 38] and TP53 is nearly universally inactivated in SCLC [3, 5], surprisingly, the combination of the MDM2 inhibitor JNJ-27291199 with etoposide and carboplatin resulted in enhanced cytotoxicity against SCLC cell lines [38].

Methylation of the TSS1500 of *IGFBP5*, which encodes an endogenous IGF1R inhibitor and is expressed at low levels in the POU2F3-regulated tuft cell-like SCLC subtype and at high levels in the subtype with high ASCL1 expression [12, 13], was associated with resistance to the mTOR inhibitor INK-128 (sapanisertib; *ρ* = 0.5107, *p*_FDR_ = 0.0896; Table 4). This association may not be mediated by *IGFBP5* transcription, as methylation of the TSS1500 region of *IGFBP5* was weakly positively associated with expression of the *IGFBP5* transcript NM_000599 (cluster ID 2598828), which was not significant (*ρ* = 0.2035, *p* = 0.1013; Table 4). Using less stringent criterion of *p*_FDR_ < 0.15, methylation of *IGFBP5* was also associated with resistance to another mTOR inhibitor, BEZ-235, and with Aurora kinase inhibitors ENMD-2076, SNS-314, and MLN-8237 (0.4549 ≤ *ρ* ≤ 0.4881, Supplementary Table 8). Neither individual probes nor gene regions of either *IGF1R* or *POU2F3*, the products of which are involved in regulatory cascades downstream or upstream of IGFBP5 [12, 13], were strongly associated with any agents.

The probes cg25627144 and cg18124721 in the TSS200 of the *DLL3* (delta-like ligand 3) gene and the entire TSS200 region were associated with the Bcl-2 inhibitor ABT-737 (*ρ* = 0.5506 and 0.4850, *p*_FDR_ = 0.0515 and 0.1366 for probes; *ρ* = 0.4762, *p*_FDR_ = 0.1260 for TSS200; Table 3, Supplementary Tables 6, 7 and 8), and cg25627144 was also associated with another Bcl-2 inhibitor, ABT-263 (*ρ* = 0.4926, *p*_FDR_ = 0.1282; Supplementary Table 7). *DLL3*, encoding a Notch pathway regulator and a promising clinical target in SCLC treatment, is upregulated in ASCL1-high SCLC tumors; in contrast, *DLL3* expression is diminished in RB wild-type tumors and in SCLC tumors not expressing neuroendocrine markers [2, 3, 39]. Methylation of all *DLL3* probes was significantly negatively associated with expression of the *DLL3* transcript, NM_016941 (cluster ID 3833122; *ρ* = − 0.4426, *p* = 0.0002 for the strongest association). Expression of that transcript was associated with sensitivity to ABT-737 (*r* = 0.4139, *p* = 0.0005). Association of lower *DLL3* methylation and increased expression with sensitivity to Bcl-2 inhibitors suggest that the use of the Bcl-2 inhibitors in the ASCL1-high SCLC lineage may be considered. While methylation of individual probes and of the upstream region (TSS200 and TSS1500) of *ASCL1* was modestly positively correlated with ABT-737, this association was not significant after adjustment for multiple testing (*ρ* ≤ 0.3687, *p*_O_ ≥ 0.0023, *p*_FDR_ ≥ 0.4024 for probes; *ρ* = 0.2862, *p*_O_ = 0.0198, *p*_FDR_ ≥ 0.4024 for the TSS200). Response to ABT-737 was associated with multiple genes (Tables 3 and 4; Supplementary Tables 6, 7 and 8), indicating that it may involve a complex interplay of factors and may not be limited to specific SCLC lineages.

Despite this and other modest non-significant correlations, methylation of master SCLC lineage regulators *ASCL1*, *NEUROD1*, and *POU2F3* [2] was not significantly associated with any agents after adjustment for multiple testing. Further examination of *SCLC* lineage drivers [2, 3] showed that methylation of the probe cg20782778 in the body of *YAP1* (encoding yes-associated protein 1 which regulates the Hippo pathway [2, 65]) was associated with resistance to the mTOR inhibitor rapamycin; *ρ* = 0.4904, *p*_O_*=* 2.91 × 10^−5^, *p*_FDR_ = 0.1282; Supplementary Tables 6 and 7. Methylation of that probe was negatively correlated with expression of the *YAP1* transcript NM_001130145 (cluster ID 3346453; *ρ* = − 0.4549, *p =* 0.0001; Supplementary Table 6), which was weakly negatively associated with log(IC50) of rapamycin (*r* = − 0.2218, *p* = 0.0712). We observed additional associations in the same direction of the probe cg20782778 in the gene body and of the upstream regions of *YAP1* with resistance to other mTOR inhibitors; however, they did not reach statistical significance after adjustment for multiple testing (*ρ* = 0.4345, *p*_O_ = 0.0003, *p*_FDR_ = 0.2215 for cg20782778 and log(IC50) of MK-8669; *ρ* = 0.4128, *p*_O_ = 0.0006, *p*_FDR_ = 0.2253 between TSS1500 and log(IC50) of sapanisertib; data not shown). Our results suggest an association between decreased *YAP1* methylation, increased *YAP1* transcription, and increased sensitivity to mTOR inhibitors. They are in agreement with a report that the YAP1-high SCLC subtype may be sensitive to mTOR inhibitors [65]. That study also found an association between increased expression of another Hippo pathway regulator, *TAZ*, and rapamycin sensitivity in SCLC [65]. In our data, associations of *TAZ* methylation and expression with rapamycin sensitivity were in the same direction as those for *YAP1*, but weaker*.* Methylation of TSS200 of *TAZ* was weakly associated with resistance to rapamycin (*ρ* = 0.2890, *p*_O_ = 0.0186), in the same direction as that of *YAP1*. *TAZ* expression was weakly associated with log(IC50) of rapamycin in the same direction as that of *YAP1* (*r* = − 0.2813, *p* = 0.0211).

Amplification and overexpression of *MYC*, which encodes *c-*Myc, has been associated with SCLC sensitivity to Aurora kinase inhibitors and with a loss of neuroendocrine markers and the ASCL1-negative and NEUROD1-positive lineage of SCLC [2, 5, 8, 10], including an inverse correlation between *MYC* and *ASCL1* expression in this dataset [24]. In contrast to *MYC*, expression of *MYCL1* is elevated in ASCL1-high SCLC tumors [2]. In our dataset, multiple probes in the bodies of *MYC* and *MYCL1* and in the upstream region and the body of *MYCN* showed modest associations (0.4060 ≤ |*ρ*| ≤ 0.5432) with multiple agents. While some of these correlations were not significant after adjustment for multiple testing (8.68 × 10^−5^ ≤ *p*_O_ ≤ 0.0006, *p*_FDR_ ≥ 0.1657; data not shown), *MYC* probes cg08526705 and cg00163372 were associated with resistance to the PLK-1 inhibitor TAK-960 (statistically significant for cg08526705 in the body of *MYC* and TAK-960, *ρ* = 0.5432, *p*_O_*=* 2.45 × 10^−5^, *p*_FDR_ = 0.0540; Table 3, Supplementary Tables 6 and 7) and to Aurora kinase inhibitors SCH-1473759 and GSK-461364, consistent with earlier studies of SCLC and with interaction of Aurora kinase A with *c-*Myc [8, 10, 58]. In *MYCL1*, cg00295382 was associated with TAK-960, and in *MYCN*, cg06520300 and cg17360299 were associated with resistance to the Wnt inhibitor salinomycin (VS-507), and cg04609952 was associated with sensitivity to the mTOR inhibitor MK-8669. In agreement with individual probes, methylation of the 5′UTR region of *MYCN* was correlated with resistance to VS-507 (salinomycin; *ρ* = 0.4552, *p*_O_*=* 0.0001, *p*_FDR_ = 0.1406; Supplementary Table 8). *MYC*, *MYCL1*, and *MYCN* are recurrently amplified in SCLC [8, 66], which results in their overexpression and could potentially increase their methylation measures. Consistent with the effect of *MYC* amplification, all *MYC* probes associated with drug response were positively correlated with expression of the *MYC* transcript NM_002467, including some statistically significant associations (*ρ* = 0.2652, *p* = 0.0314 for cg00163372; Table 3). Intriguingly, the probe cg00295382 of *MYCL1* and the 5′UTR of *MYCN* were significantly negatively associated with gene expression (*ρ* = − 0.6744, *p* = 5.37 × 10^−10^ for cg00295382 of *MYCL1* and *ρ* = − 0.4603, *p* = 0.0001 for the 5′UTR of *MYCN*). Accordingly, expression of the *MYCN* transcript NM_005378 (Affymetrix cluster ID 3349293) was associated with sensitivity to salinomycin (*ρ* = − 0.2376, *p* = 0.0529; Supplementary Table 8), in the opposite direction from the 5′UTR methylation. The 5′UTR of *MYCN* contains important regulatory elements for promoter activity in neuroblastoma [67]. This suggests a possibility that while *MYCN* amplification may have the main effect on *MYCN* overexpression, in some cases methylation of its 5′UTR may affect its expression in SCLC and influence the response to salinomycin.

Methylation of probes in the non-neuroendocrine lineage marker *EPHA2* [5], predominantly in the upstream regions, was associated with resistance to Aurora kinase inhibitors ENMD-2076, SCH-1473759, and TAK-901, and the PLK-1 inhibitor GSK-461364 (0.4869 ≤ *ρ* ≤ 0.5233, 0.0876 ≤ *p*_FDR_ ≤ 0.1343; Table 3, Supplementary Tables 6 and 7). Consistent with association of individual *EPHA2* probes, methylation of its TSS200 was associated with resistance to ENMD-2076, SCH-1473759, and TAK-901 (0.4549 ≤ *ρ* ≤ 0.4893, 0.1163 ≤ *p*_FDR_ ≤ 0.1406; Supplementary Table 8). Methylation of all *EPHA2* probes and of the TSS200 region were negatively correlated with *EPHA2* transcript expression (NM_004431, cluster ID 2397948; *ρ* = − 0.3832, *p*_O_ = 0.0015 for TSS200; Supplementary Table 8). Association of *EPHA2* expression with log(IC50) of each Aurora kinase inhibitor was very weak but negative for all agents ENMD-2076, SCH-1473759, and TAK-901 (*r* ≥ − 0.2090, *p* ≥ 0.0897; Supplementary Table 8), suggesting that cell lines with higher *EPHA2* expression and a tendency for lower *EPHA2* methylation were slightly more sensitive to these agents.

The 3′UTR of the non-neuroendocrine lineage marker *CD151* [5] was associated with sensitivity to Bcl-2 inhibitors ABT-263 and ABT-737 (*ρ* = − 0.4651 and − 0.4809, *p*_FDR_ = 0.1347 and 0.1260, respectively; Supplementary Table 8). Consistent with this association, the probe cg24508095 in the 3′UTR was associated with sensitivity to ABT-737 (*ρ* = − 0.4809, *p*_O_ = 4.37 × 10^−5^, *p*_FDR_ = 0.1484; Supplementary Table 7). While the role of the 3′UTR is unclear, its methylation was weakly negatively associated with expression of the *CD151* transcript NM_004357 (cluster ID 3316344; *ρ* = − 0.2970, *p* = 0.0154), and increased *CD151* expression was associated with resistance to both agents (*r* = 0.3781, *p* = 0.0016 for ABT-263; *r =* 0.4266, *p* = 0.0003 for ABT-737; Supplementary Table 8).

In recent studies, gene and protein expression of *SLFN11* have emerged as significant predictors of *SCLC* response to DNA-damaging agents including topoisomerase I and II inhibitors, PARP inhibitors, and platinum compounds [3, 23, 24, 38, 68–71]. The SLFN11 protein is epigenetically silenced by EZH2 via H3K27me3 histone methylation, leading to resistance to a combined therapy of etoposide and a platinum compound [23]. While association of *SLFN11* expression with sensitivity to DNA-damaging agents in this dataset was highly significant [24, 38], correlations of methylation of *SLFN11* probes and regions with drug response had *p*_FDR_ > 0.1. The strongest associations of *SLFN11* probes and regions, with *p*_FDR_ < 0.15, are listed in Supplementary Tables 6, 7 and 8. These include correlations of methylation of the gene body with sensitivity to BMN-673 (talazoparib), teniposide, and topotecan (− 0.4871 ≤ *ρ* ≤ − 0.4617, 0.1163 ≤ *p*_FDR_ ≤ 0.1347; Supplementary Table 8). In addition, methylation of the upstream regions and of the body of *SLFN11* was associated with a variety of topoisomerase inhibitors and PARP inhibitors with *p*_FDR_ > 0.15, including correlations of topotecan, teniposide, and talazoparib with the 3′UTR, and correlations of the 3′UTR and gene body with etoposide, MK-4827, olaparib, and PF-01367338 or rucaparib (− 0.4436 ≤ *ρ* ≤ − 0.4110, *p*_FDR_ ≥ 0.1731).

Interestingly, methylation of the probes in the upstream regions was associated with drug resistance, whereas probes in the body and the 3′UTR of *SLFN11* were associated with sensitivity to DNA damaging agents. For example, cg18108623 in the TSS1500 had *ρ* = 0.4989 and 0.4811 for teniposide and topotecan, respectively, whereas cg18124488 in the gene body had *ρ* = − 0.4871 with topotecan (0.1251 ≤ *p*_FDR_ ≤ 0.1484; Supplementary Table 7). We also observed many modest non-significant associations of methylation of probes in the TSS1500 and TSS200 upstream regions with resistance, and of probes in the gene body and 3′UTR with sensitivity to teniposide, talazoparib, etoposide, topotecan, olaparib, MK-4827, and PF-01367338 (− 0.4638 ≤ *ρ* ≤ − 0.4002 for probes in the gene body and the 3′UTR, 0.4079 ≤ *ρ* ≤ 0.4679 for probes in the 5′UTR; *p*_FDR_ ≥ 0.1657; data not shown). Despite multiple associations of probes in the body and in the 3′UTR with sensitivity to DNA-damaging agents, methylation of the gene body was very strongly positively correlated with expression of the *SLFN11* transcript NM_001104587 (cluster ID 3753500, *ρ* = 0.7475, *p* = 5.61 × 10^−13^). In contrast, methylation of the probes cg03668718, cg18108623, and cg18608369 in the TSS1500 and TSS200 was not only associated with resistance to DNA-damaging agents, but it was also strongly negatively associated with transcript expression (− 0.7134 ≤ *ρ* ≤ − 0.6129, 1.80 × 10^−11^ ≤ *p*_O_ ≤ 4.50 × 10^−8^ for probes; *ρ* = − 0.6403, *p*_O_ = 7.07 × 10^−9^ for TSS1500; *ρ* = − 0.6935, *p*_O_ = 1.09 × 10^−10^ for TSS200; selected examples are shown in Supplementary Figure 3 A-F). The SCLC-A lineage [2] had an increased number of cell lines with higher methylation of the *SLFN11* TSS200 region (Supplementary Figure 3G). Our results suggest that methylation of the *SLFN11* upstream regions but not of its gene body or the 3′UTR may downregulate its expression, increasing drug resistance. They are consistent with our earlier analysis of NCI-60 cell lines, which did not include SCLC, that showed the effect of methylation of these and additional *SLFN11* probes in the CpG island in the upstream promoter region on resistance to platinum drugs [20].

**Fig. 4:**
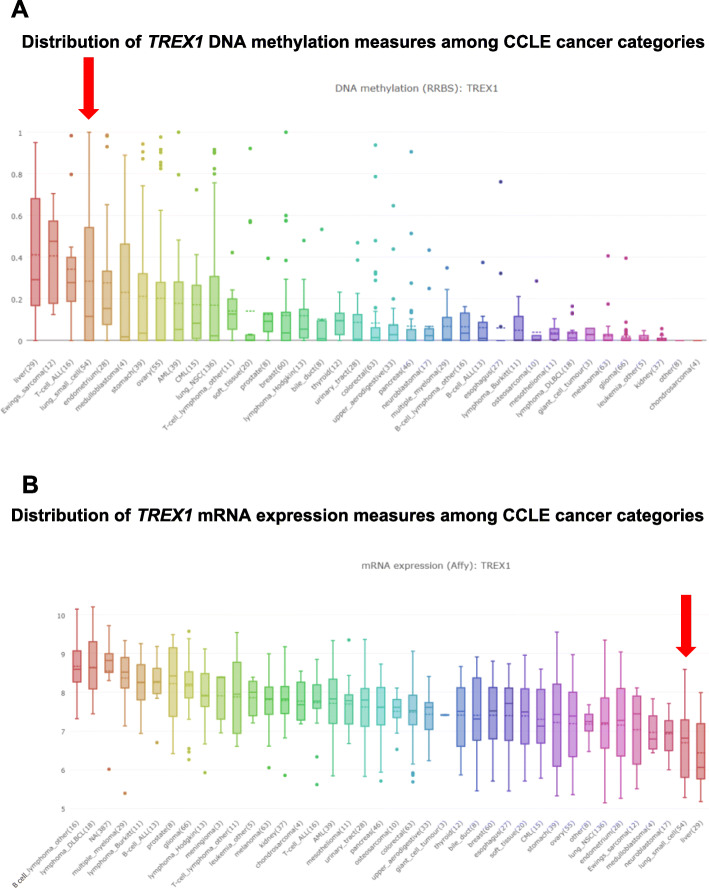
Distribution of **a** DNA methylation and **b** mRNA expression levels of *TREX1* among different cancer categories in the CCLE dataset. Boxplots of *TREX1* DNA methylation measures and mRNA expression measures among different cancer categories of the CCLE cell lines were generated and downloaded from the CCLE online resource [43, 44]. Red vertical arrows highlight the *TREX1* DNA methylation and gene expression values for the SCLC cell lines

*EZH2*, commonly overexpressed in SCLC, is involved in upregulation of DNA methyltransferases, increased methylation, and epigenetic silencing of *SLFN11* [3, 23]. The 5′UTR of *EZH2* and several probes in that region were associated with resistance to the Aurora kinase inhibitors AMG-900, CYC-116, and SNS-314, and to the FGFR inhibitor BGJ-398 (0.4634 ≤ *ρ* ≤ 0.4988, 2.64 × 10^−5^ ≤ *p*_O_ ≤ 2.08 × 10^−6^, 0.1094 ≤ *p*_FDR_ ≤ 0.1247 for the 5′UTR region; Fig. 4; Supplementary Table 8; information about individual probes is provided in Supplementary Data 2D). At the epigenome-wide level, the 5′UTR was associated with resistance to the lysophosphatidic acid acyltransferase-β inhibitor CT-32228 (*ρ* = 0.5813, *p*_O_ = 3.08 × 10^−7^, *p*_FDR_ = 0.1396; Supplementary Table 5; Supplementary Figure 4). No other *EZH2* regions were associated with drug response. The role of methylation of the 5′UTR is unclear because it was positively associated with transcript expression (NM_004456, cluster ID 3078348), and *EZH2* expression was not associated with response to any of these agents (|*r*| < 0.15; Supplementary Tables 5 and 8).

*BCL2* is one of the most highly differentially methylated genes in SCLC, with variable methylation and expression among SCLC subtypes; by contrast, it is epigenetically silenced in normal lung tissue [11]. Within its gene body, several probes showed different directions of association with response to Bcl inhibitors GX15-070 and ABT-737 and the Aurora A kinase/tyrosine kinase inhibitor ENMD-2076 in the analysis of candidate genes and preselected agents, and one probe showed a trend for association with the CDK inhibitor R-547 at the epigenome-wide level (Supplementary Data 2D and Supplementary Tables 6 and 7).

## Discussion

Our study did not pre-select any DNA methylation probes. We analyzed probes with high-quality measures based on the detection *p* values and filtered out the probes overlapping with SNPs [26, 27]. After filtering, all the remaining probes were included in computation of gene region-averaged methylation values. We reported all top probes and all gene regions that were strongly associated with drug response and satisfied the significance thresholds. Our analyses were performed at the epigenome level and also for candidate genes and drug agents relevant to SCLC pathogenesis and potential treatment. Alternative approaches previously employed in several analyses of other cancer cell line methylome datasets included prioritization and selection of probes based on their proximity to the location of genes, promoter regions, and CpG islands, and/or association with transcript expression, and the focus on gene-averaged or promoter region-averaged methylation measures [19, 44]. The application of different analytical approaches to large epigenome datasets may help find novel targets through different prioritization and ranking of the significant results, and it may help provide additional ways for validation of the findings. Our analysis of *TREX1* showed that while our novel initial findings of association of gene expression and drug response with DNA methylation measures were based on all probes and gene regions without prior pre-selection, these associations were validated by the correlations of the CCLE and GDSC data which were based on promoter-enriched methylation measures.

Our study focused on significant associations of DNA methylation-based measures in probes and gene regions with drug response. We further explored whether such associations could be explained by the strength and the direction of the correlations of DNA methylation with gene expression. The genes most likely to have the direct functional effect of DNA methylation on treatment response were those genes that had methylation data strongly associated with chemosensitivity and also had negative correlations between methylation and transcript expression, accompanied by significant correlations between expression and drug response in the opposite direction from methylation. For those genes that had a strong positive correlation between DNA methylation and gene expression, further investigations are needed, including the detailed analysis of their possible copy number gain or loss, which would similarly affect both DNA methylation and expression measures, and/or the transcriptional regulation of gene expression via mechanisms other than DNA methylation. Previous complementary approaches to the studies of drug response have prioritized associations based on the strength of correlation between gene expression with drug response [38] or used the correlation between methylation and gene expression as selection criteria for methylation measures [19]. The availability of both expression and methylation data, along with drug response measures for this rich dataset, provides opportunities for further integrative analyses. In a parallel effort, we have been developing a public online resource, SCLC-CellMiner [72] which integrates various molecular measurements for SCLC cell lines including whole exome sequencing, gene expression, miRNA expression, methylation, and copy number data, along with drug response information presented as drug activity. This resource will allow users to query and visualize the correlations for genes of interest.

Our association analysis of DNA methylation with cell line response to chemotherapy agents elucidated very strong associations of *TREX1* methylation and expression with SCLC sensitivity to Aurora kinase inhibitors, antimitotic agents, a CDK inhibitor, and an ATR inhibitor. DNase III, which is encoded by *TREX1*, generates single-stranded DNA (ssDNA) during resolution of chromatin bridges under telomere crisis in malignant cells, and this TREX1 activity has been proposed to play a role in chromothripsis [73, 74]. TREX1 is also involved in the maintenance of immune tolerance to self-DNA of the cytosolic DNA-sensing pathway, acting in an opposing manner to the STING regulator [75]. The absence of *TREX1* or inactivating mutations in TREX1 result in the upregulation of the cGAS-STING-mediated type I interferon response and systemic inflammation [76]. These roles of TREX1 may provide an explanation for the association of *TREX1* methylation and expression with response to Aurora kinase inhibitors, agents targeting microtubule assembly, and with a CDK inhibitor, as these agents interfere with different aspects of cell cycle, mitosis, and cell proliferation. Association of *TREX1* methylation with SCLC response to the ATR inhibitor Cpd45 may be consistent with the previously reported TREX1 upregulation in cancer cells in response to DNA damaging agents and to UV light, and roles in sensitivity and response to DNA-damaging agents, DNA repair, and/or DNA degradation after drug treatment [50–52], including degradation of ssDNA fragments by TREX1 and TREX1 interaction with PARP1 (poly(ADP-ribose) polymerase-1) in response to DNA damage [77, 78].

SCLC cell lines had among the highest *TREX1* methylation and among the lowest *TREX1* expression in the CCLE (Fig. 4), suggesting the possibility that low TREX1 in SCLC may lead to vulnerability, increasing SCLC susceptibility to treatment by Aurora kinase inhibitors. Therefore, TREX1 may represent a novel molecular marker or target in SCLC.

Our analysis of DNA methylation measures and drug response utilized SCLC cell line data. Previous studies have shown that SCLC cell lines have higher overall DNA methylation than do primary tumors [11]. The absence of primary tumor samples is a limitation of our study. Despite reported methylation differences between the cell lines and primary tumor data and the lack of patient specimens in our analysis, associations identified in our study using cell line data, including those for *TREX1*, suggest novel therapeutic targets for potential clinical use. A broader intriguing question is whether TREX1 would be a useful molecular target for treatment of other cancers. A combined analysis of breast cancer patients in the METABRIC (Molecular Taxonomy of Breast Cancer International Consortium) cohort suggested a lower survival probability in patients with diminished *TREX1* expression [77]. However, the differences in patient survival in that combined dataset could also be attributed to other molecular differences among various breast cancer subtypes. By contrast, analysis of a *BRCA1*-deficient, p53-deficient genetically engineered mouse model of triple negative breast cancer (TNBC) and analysis of TNBC cell lines showed that activation of the cGAS/STING pathway within the tumor increased the efficacy of PARP inhibitors through tumor cell response and increased immune activation in the tumor microenvironment, with more profound benefits in homologous recombination repair-deficient tumor cells [79]. Similarly, the STING pathway is activated in SCLC tumor cells in response to PARP inhibition and CHK1 inhibition [80]. These observations provide an additional argument that TREX1, an antagonist of the STING-activated molecular pathway, may present an attractive target in SCLC treatment and possibly in other cancer categories.

Lineage-specific differentiation of SCLC tumors based on expression of several master regulators [2] has previously been associated with distinct methylation patterns [11, 15]. While many associations of SCLC methylation patterns with drug response were not specific to particular SCLC lineages in our dataset, several lineage-specific associations, e.g., that of *YAP1* methylation and expression with rapamycin confirming the results from an earlier study [65], and methylation and expression of *DLL3*, which is upregulated in ASCL1-high tumors, with Bcl-2 inhibitors were identified. Among non-neuroendocrine lineage markers, methylation of *EPHA2* was associated with Aurora kinase inhibitors and a PLK-1 inhibitor, whereas that of *CD151* was associated with Bcl-2 inhibitors.

We did not observe any strong association pattern between methylation and expression of *TREX1* regions with SCLC lineage categories. Methylation of *TREX1* TSS1500 and TSS200 regions was weakly associated with *ASCL1* expression (Pearson *r* = − 0.309, *p*_O_ = 0.0117 for TSS200), whereas *TREX1* expression was weakly associated with *NEUROD1* expression (*r* = − 0.368, *p*_O_ = 0.02953). Interestingly, whereas *c*-Myc directly upregulates expression of Aurora kinase genes and promotes a neuroendocrine-low subtype of SCLC which is sensitive to Aurora kinase inhibitors [10, 57], the expression of *TREX1* was not associated with transcriptional levels of either *MYC*, *MYCL*, or *MYCN* (|*r|* < 0.13), even though methylation of the 5′UTR and the first exon of *TREX1* was weakly associated with *MYC* expression (*r* = 0.328 and 0.326, *p*_O_ = 0.0076 and 0.0072, respectively; data not shown).

DNA methylation in SCLC has been reported to be strongly associated with *EZH2* expression [11]. Decreased methylation of the 5′UTR of *EZH2* and lower *EZH2* expression were more common in SCLC cell lines from subtypes other than SCLC-A (Supplementary Figure 4C and D). Considering that no other *EZH2* regions were associated with drug response or with SCLC lineage differentiation, and that methylation of the 5′UTR and *EZH2* expression were positively correlated, interpretation of the associations of the 5′UTR requires further biological investigation. We observed correlations between *EZH2* expression and expression of *YAP1* (transcript cluster ID 3346453; *r* = − 0.397, *p*_O_ = 0.0010), *MYCN* (2470838; *r* = 0.328, *p*_O_ = 0.0071) and, most strongly, *MYC* (3115504; *r* = − 0.512, *p*_O_ = 1.098 × 10^−5^), which suggest a potential mechanism linking expression of SCLC master lineage regulators and epigenetic pathways of lineage differentiation; however, these correlations do not provide evidence that this mechanism could involve the methylation of the *EZH2* promoter region.

Consistent with earlier analyses of the NCI-60 cell lines [19, 20] and with growing evidence for the role of epigenetic mechanisms in *SLFN11* inactivation [21, 69, 81], we found that increased methylation of the *SLFN11* promoter was negatively correlated with *SLFN11* expression and increased resistance of SCLC lines to DNA-damaging agents (Supplementary Figure 3**)**. Association of *SCLC* promoter methylation with gene expression and with drug sensitivity are in agreement with the findings of He et al. [82], who showed that increased methylation of the *SLFN11* promoter in colorectal cancer cell lines and primary tumors led to downregulation of *SLFN11* expression, increased tumor resistance to cisplatin, and decreased the 5-year overall patient survival and 5-year progression-free survival.

Our study utilized a large comprehensive dataset of methylation measures derived from the Illumina Infinium MethylationEPIC BeadChip array, which substantially increased the density of available methylation measures for SCLC compared with data from earlier SCLC studies which utilized lower density measures [11, 15]. We analyzed a large set of drug screening measures and genome-wide transcript and miRNA expression measures. We conducted a comprehensive analysis of association of pre-treatment SCLC methylation and expression with response to multiple approved and investigational chemotherapy agents. We focused on the gene targets identified by strong associations of methylation signals with drug response as the primary biological leads.

Our findings of methylation signals correlated with drug response confirmed multiple previously identified SCLC associations with drug response from gene expression studies, e.g., those for *SLFN11* and *YAP1.* Several genes, e.g., *EPAS1* (*HIF2A*), *EZH2*, and *BCL2*, that were previously suggested to be involved in SCLC drug response based on their expression, protein activity, drug action, and/or distinct methylation patterns in SCLC [11, 23, 24, 83, 84] showed complex patterns of associations between methylation and expression (*EPAS1* and *EZH2*) or between methylation and drug response (*BCL2).* This suggests that the expression and activity of their products may be affected by other factors in addition to DNA methylation*.* We observed a number of correlations which involved candidate therapy agents that had been previously identified in earlier studies which used in vitro data, animal models, and clinical data. We report multiple associations between such agents and genetic methylation markers, which could potentially be utilized to stratify SCLC patients for therapy response. We also report multiple novel associations of gene methylation with drug responses. These results suggest new therapeutic targets and drug combinations for SCLC treatment which may be beneficial for specific epigenetic tumor landscapes. Some of the newly discovered associations, e.g., those for *TREX1*, may suggest potential novel direct drug targets in tumors with residual *TREX1* expression, and also add to the accumulating body of evidence about the importance of upregulation of the cGAS/STING pathway in treatment of cancer patients [79, 80, 85]. Similarly, other associations reported in our study may suggest novel therapy targets, indicate potential broader molecular pathways of interest, and provide biomarkers for patient stratification. Due to the dynamic nature of DNA methylation and expression, and rapid acquisition of chemoresistance by SCLC cells in response to treatment, it would be important to continue future investigations of drug response by comparing pre- and post-treatment DNA methylation and expression levels and to further analyze the dynamic nature of DNA methylation, expression, alternative splicing, and post-transcriptional modifications that may drive the chemoresistance of tumor cells.

## Conclusions

We completed a comprehensive analysis of a large SCLC DNA methylation dataset and examined associations of de novo DNA methylation with response to 526 chemotherapeutic agents. Our analysis of methylation data confirmed previously known association results between gene expression and drug response for several known genes, e.g., between *SLFN11* and DNA damaging agents and between *YAP1* and rapamycin. We discovered multiple novel associations which suggest potential targets for single agent treatment and drug combination therapies. Methylation and expression of *TREX1* were associated with response to multiple drug categories, which may suggest a possible mechanism of vulnerability to Aurora kinase inhibitors for SCLC, which has low *TREX1* expression compared to other cancer categories. These associations also suggest that targeting TREX1 may provide important therapeutic benefits in those SCLC tumors which have residual *TREX1* expression.

## Supplementary information

**Additional file 1: Supplementary Table 1.** SCLC cell lines used in correlation analysis of methylation, transcript and miRNA expression, and drug response data.

**Additional file 2: Supplementary Table 2.** List of selected single agents, their target genes and additional genes potentially involved in their response, which were examined in detailed DNA methylation analysis.

**Additional file 3: Supplementary Table 3.** List of methylation probes with the strongest correlations with log(IC50) measure of drug response, which satisfied the Spearman correlation *p*_O_ < 9.42 × 10^-8^ in the epigenome-wide analysis of all antitumor agents.

**Additional file 4: Supplementary Table 4.** Gene level summary for methylation probe-drug correlations involving at least one methylation probe with *p*_O_ < 9.42 x 10^-8^ in the epigenome-wide analysis of all antitumor agents.

**Additional file 5: Supplementary Table 5.** Gene regions with FDR adjusted *p*-value *p*_FDR_ < 0.15 for Spearman correlations of average gene region methylation with log(IC50) of antitumor agents in the epigenome-wide analysis of all antitumor agents.

**Additional file 6: Supplementary Table 6.** Candidate genes with methylation probes that had correlations with log(IC50) of candidate antitumor agents satisfying *p*_FDR_ < 0.15.

**Additional file 7: Supplementary Table 7.** List of methylation probes in candidate genes which satisfied the FDR adjusted *p*-value *p*_FDR_ < 0.15 for Spearman correlations with log(IC50) measure of response to the candidate agents listed in Supplementary Table 2.

**Additional file 8: Supplementary Table 8.** Correlations of candidate gene regions with log(IC50) of candidate antitumor agents that satisfied *p*_FDR_ < 0.15.

**Additional file 9: Supplementary Data 1.** Methylation beta-values and Illumina EPIC array annotation of probes which passed QC and filtering and are located within or adjacent to the *SNED1* gene.

**Additional file 10: Supplementary Data 2.** A. Associations of methylation beta-values of individual probes in the *SNED1* gene with log(IC50) of the HDAC inhibitor 4SC-202 (NSC 759905). B. Associations of methylation beta-values of individual probes in *SNED1* with log(IC50) of the HDAC inhibitor vorinostat (NSC 701852). C. Associations of *SNED1* transcript expression with response to HDAC inhibitors. D. Additional information about associations of probes in selected genes.

**Additional file 11: Supplementary Data 3.** Methylation beta-values and Illumina EPIC array annotation of probes which passed QC and filtering and are located within or adjacent to the *TREX1* gene.

**Additional file 12: Supplementary Data 4.** Average methylation of *TREX1* gene regions.

**Additional file 13: Supplementary Figure 1.** Scatterplots of *CEP350* DNA methylation and measures of SCLC drug sensitivity. A. Methylation of the 3’UTR region of *CEP350* vs log(IC50) of ABT-348. Horizontal scale represents average methylation beta-values of the 3’UTR of *CEP350*, whereas the vertical scale represents the log(IC50) values. B. Methylation of the 3’UTR region of *CEP350* vs log(IC50) of TAK-901. Horizontal scale represents the average methylation beta-values of the *CEP350* 3’UTR region, whereas the vertical scale represents the log(IC50) values of TAK-901.

**Additional file 14: Supplementary Figure 2.** Scatterplots of *EPAS1* probe DNA methylation, transcript expression, and log(IC50) of AMG-900. A. Methylation of the *EPAS1* probe cg08937075 vs log(IC50) of AMG-900. Horizontal scale represents methylation beta-values, whereas the vertical scale represents the log(IC50) values. B. Methylation of the *EPAS1* probe cg08937075 vs expression of the *EPAS1* transcript 2480383. Horizontal scale represents the methylation beta-values, whereas the vertical scale shows the log_2_-transformed gene expression.

**Additional file 15: Supplementary Figure 3.** A-F. Scatterplots of *SLFN11* probe and region DNA methylation, transcript expression, and drug response. Plotted are methylation beta-values, log_2_-transformed expression of the *SLFN11* transcript 3753500, and log(IC50) measures of drug response. G. Methylation of the TSS200 of *SLFN11* (vertical sidebar) plotted against the heatmap of SCLC cell line clustering based on expression of the SCLC lineage marker genes. In the heatmap, rows represent SCLC cell lines, whereas columns represent log_2_-transformed expression of *NEUROD1, ASCL1, ASLC2, INSM1, YAP1,* and *POU2F3.* For those cell lines that had previously reported SCLC lineage subtype classification [2], their lineage subtype assignments are listed with their cell line names in the vertical right column of row labels.

**Additional file 16: Supplementary Figure 4.** A. Scatterplot of DNA methylation (horizontal scale) of the 5’UTR of *EZH2* vs log(IC50) of ABT-348 (vertical scale). B. Scatterplot of DNA methylation of the 5’UTR of *EZH2* (horizontal scale) vs log(IC50) of ABT-348 CT-32228 (vertical scale). C. Methylation of the 5’UTR of *EZH2* (vertical sidebar) plotted against the heatmap of SCLC cell line classification based on lineage marker expression. D. *EZH2* expression (vertical sidebar) plotted against the heatmap of SCLC cell line clustering based on expression of the SCLC lineage marker genes. In the heatmap, rows represent SCLC cell lines, whereas columns represent log_2_-transformed expression of *NEUROD1, ASCL1, ASLC2, INSM1, YAP1,* and *POU2F3.* For those cell lines that had previously reported SCLC lineage subtype classification [2], their lineage subtype assignments are listed with their cell line names in the vertical right column of row labels.

## Data Availability

Methylation data for the 760,637 filtered probes which passed the QC and did not overlap with single nucleotide variants, average methylation values for all gene regions, the drug and compound response data, and transcript and miRNA expression measures adjusted for batch effects are available from the NCI Small Cell Lung Cancer Project site [86]. DNA methylation and transcript and miRNA expression data are also available from NCBI GEO (accession numbers GSE145156, GSE73160, and GSE73161, respectively).

## Abbreviations

AML: Acute myeloid leukemia
ATR: Ataxia telangiectasia and Rad3-related protein
ASCL1: Achaete-scute homolog 1
CDK: Cyclin-dependent kinase
CEP350: Centrosomal protein 350
DLL3: Delta-like ligand 3
DMSO: Dimethyl sulfoxide
FDR: False discovery rate
GEO: Gene Expression Omnibus
GSK-3: Glycogen synthase kinase 3
HDAC: Histone deacetylase
HIF2α: Hypoxia-inducible factor 2α
KSP: Kinesin spindle protein
LSD1: Histone-modifying protein lysine demethylase 1A
LPAAT-β: Lysophosphatidic acid acyltransferase-β
METABRIC: Molecular Taxonomy of Breast Cancer International Consortium
MLPH: Melanophilin
NCI: National Cancer Institute
NEUROD1: Neurogenic differentiation factor 1
QC: Quality control
PARP1: Poly(ADP-ribose) polymerase-1
*p*_FDR_: FDR-adjusted *p* value
PLK: Polo-like kinase
*p*_O_: *p* value prior to FDR adjustment
SCLC: Small cell lung cancer
SNP: Single nucleotide polymorphism
TSS: Transcriptional start site
SNED1: Sushi, nidogen and EGF like domains 1
UCSC: University of California, Santa Cruz
UTR: Untranslated region
YAP1: Yes-associated protein 1
