# Supplementary material for: Epigenome-wide DNA methylation analysis of small cell lung cancer cell lines suggests potential chemotherapy targets
Source: Clin Epigenetics. 2020 Jun 25;12:93. doi: 10.1186/s13148-020-00876-8 (PMC7318526; doi:10.1186/s13148-020-00876-8)
Supplement: Supplementary file 1 — Additional file 1: Supplementary Table 1. SCLC cell lines used in correlation analysis of methylation, transcript and miRNA expression, and drug response data. [file 13148_2020_876_MOESM1_ESM.pdf]

Supplementary Table 1. SCLC lines used in correlation analysis of methylation, transcript and miRNA expression, and drug response data

COLO-668	NCI-H209
COR-L32	NCI-H2107
COR-L47	NCI-H211
DMS-114	NCI-H2141
DMS-187	NCI-H2171
DMS 273	NCI-H2198
DMS-53	NCI-H220
DMS-79	NCI-H2330
LXFS-605L	NCI-H250
LXFS-650L	NCI-H345
NCI-H1048	NCI-H378
NCI-H1092	NCI-H446
NCI-H1105	NCI-H510
NCI-H128	NCI-H524
NCI-H1341	NCI-H526
NCI-H1417	NCI-H660
NCI-H1436	NCI-H69
NCI-H146	NCI-H69-CPR
NCI-H1522	NCI-H69-LX10
NCI-H1618	NCI-H69-VCR
NCI-H1672	NCI-H711
NCI-H1688	NCI-H719
NCI-H1694	NCI-H720
NCI-H1836	NCI-H735
NCI-H187	NCI-H748
NCI-H1876	NCI-H774
NCI-H1882	NCI-H82
NCI-H1930	NCI-H841
NCI-H196	NCI-H847
NCI-H1963	NCI-H865
NCI-H2029	NCI-H889
NCI-H2066	SHP-77
NCI-H2081	SW-1271

The 66 SCLC cell lines listed in the table were included in Spearman pairwise correlations of methylation measures vs log(IC50) and of methylation data vs $\log_2$ of transcript and miRNA expression. The Pearson correlation analysis of $\log_2$ of transcript and miRNA expression included the total of 67 SCLC cell lines, including all cell lines listed in the table and an additional cell line, COR L88, which had expression and drug response measurements but no methylation measurements.
