# Supplementary material for: Epigenome-wide DNA methylation analysis of small cell lung cancer cell lines suggests potential chemotherapy targets
Source: Clin Epigenetics. 2020 Jun 25;12:93. doi: 10.1186/s13148-020-00876-8 (PMC7318526; doi:10.1186/s13148-020-00876-8)
Supplement: Supplementary file 2 — Additional file 2: Supplementary Table 2. List of selected single agents, their target genes and additional genes potentially involved in their response, which were examined in detailed DNA methylation analysis. [file 13148_2020_876_MOESM2_ESM.pdf]

Supplementary Table 2. List of selected single agents, their target genes and additional genes potentially involved in their response, which were examined in detailed DNA methylation analysis

**Agent**	**NSC**	**Other agent names**	**Gene targets**	**Additional genes that may contribute to resistance or sensitivity**	**References**
Etoposide	141540		*TOP2A, TOP2B*	*MDM2, LIG4, XRCC6* (encoding KU70), *RAD54L* (*RAD54*), *TDP2, ATM, HDAC1, HDAC2, KAT8, SIRT1, MAGEA1, NDN, MAGED1, E2F4, BIN1, NLK, SLFN11*	[1-4]
Topotecan	609699		*TOP1, TOP1MT*	*ABCB1, SLFN11*	[1, 2, 4-7]
Teniposide	758255		*TOP2A, TOP2B*	*ABCB1, SLFN11*	[1, 2, 7, 8]
Gemcitabine	613327		*RRM1, TYMS, CMPK1*	*SLFN11, SLC29A1, SLC29A2, SLC28A1, SLC28A2, SLC28A3, DCK, NT5C2, NT5C3, CDA*	[1, 2, 6, 9]
VS-507	757437	Salinomycin	*SKP2, STAT3*		[10]
Talazoparib	767125	BMN-673, Talzenna	*PARP1, PARP2*	*BRCA1, BRCA2, PALB2, RAD51, RAD51C, RAD51D, CHEK2, ATM, MRE11A, ATR, TP53BP1, RIF1, MAD2L2 (REV7), PAXIP1 (PTIP), CHD4, XRCC6, JMJD1C, SLFN11*	[2, 11-15]
Olaparib	753686	AZD-2281	*PARP1, PARP2, PARP3, TNKS*	*BRCA1, BRCA2, PALB2, RAD51, RAD51C, RAD51D, CHEK2, ATM, MRE11A, ATR, TP53BP1, RIF1, MAD2L2, PAXIP1, CHD4, XRCC6, JMJD1C, SLFN11*	[1, 2, 12-15]
Niraparib	754355		*PARP1, PARP2*	*BRCA1, BRCA2, PALB2, RAD51, RAD51C, RAD51D, CHEK2, ATM, MRE11A, ATR, TP53BP1, RIF1, MAD2L2, PAXIP1, CHD4, XRCC6, JMJD1C, SLFN11*	[2, 12-15]
Rucaparib	756644		*PARP1, PARP2, PARP3*	*BRCA1, BRCA2, PALB2, RAD51, RAD51C, RAD51D, CHEK2, ATM, MRE11A, ATR, TP53BP1, RIF1, MAD2L2, PAXIP1, CHD4, XRCC6, JMJD1C, SLFN11*	[2, 11-15]
AZD-2461	773230		*PARP1, PARP2*	*BRCA1, BRCA2, PALB2, RAD51, RAD51C, RAD51D, CHEK2, ATM, MRE11A, ATR, TP53BP1, RIF1, MAD2L2, PAXIP1, CHD4, XRCC6, JMJD1C, SLFN11*	[12, 13, 16, 17]
ABT-263	759659	Navitoclax	*BCL2, BCL2L2 (BCLW), BCL2L1 (BCLXL), BAD*	*CTNNB1, MCL1*	[1, 2, 11]
ABT-737	758873	BRD-K56301217	*BCL2, BCL2L2, BCL2L1*		[1]
ABT-199	766270	Venetoclax, BRD-K62391742	*BCL2*		[1, 2]
AT-101	727859	BL-193	*BCL2, SMO*	*GLI1, SHH*	[18]
GX15-070	756663	Obatoclax	*BCL2, BCL2L1, BCL2L2, MCL1*		[11]

Everolimus	733504		*MTOR, FKBP1A (FKBP12)*		[2, 19]
BEZ-235	751249	Dactolisib, NVP-BEZ235, BRD-K12184916	*MTOR, PIK3CA, PIK3CB, PIK3CD, PIK3CG*		[1, 20]
MK-8669	757440	Ridaforolimus, Deforolimus	*MTOR*		[2, 16]
Sirolimus	226080	Rapamycin	*MTOR, FKBP1A, FKBP5, FKBP10, FGF2, FKBP1A*		[1, 2, 19]
RG-7603	771532	GDC-0349	*MTOR, PIK3CA*		[16, 21]
AZD-2014	767189	Vistusertib	*MTOR, AKT1, RPS6*		[16]
INK-128	764658	Sapanisertib, MLN0128	*MTOR, PIK3CA, PIK3CD, PIK3CG*		[16]
Linsitinib	756652	OSI-906, BRD-K86118762, ASP-7487	*IGF1R, INSR, INSRR*		[1, 2]
SB-743921	773261	BRD-K62358710	*KIF11 (KSP)*		[1, 16]
ARQ-621	771644		*KIF11*		[16]
Ispinesib	755386	CK-0238273, SB-715992	*KIF11*		[11, 16]
EMD-534085	763564		*KIF11*		[22, 23]
ARRY-520A	772254	Filanesib	*KIF11*		[2, 24]
TAK-960	768072		*PLK1, PLK2, PLK3, PTK2 (FAK), MYLK (MLCK), FES*		[25]
BI-2536	755983	BRD-K64890080	*PLK1, PLK2, PLK3, BRD4*	*ABCB1, ABCG2*	[1, 11, 16]
GSK-461364	754354	GSK461364	*PLK1*		[16]
MLN-8237	759677	Alisertib	*AURKA, AURKB, NEDD9*		[1, 16]
AMG-900	761069		*AURKA, AURKB, AURKC, MAPK14, TYK2, MAPK9 (JNK2), MET, TEK*		[16]
AS-703569	763930	Cenisertib	*AURKA, AURKB, AURKC, ABL1, FLT1, FLT3*		[2, 16, 26, 27]
ABT-348	765889		*AURKA, AURKB, AURKC, FLT1, KDR, FLT3, FLT4, CSF1R, KIT, PDGFRA, PDGFRB, SRC*		[28]
AZD-1152	757444	Barasertib	*AURKB*		[1, 16]

SCH-1473759	761691		*AURKA, AURKB, SRC, CHEK1, KDR, IRAK4*		[29]
SNS-314	758250		*AURKA, AURKB, AURKC, NTRK1 (TRK1, TRKA), NTRK2 (TRK2, TRKB), FLT4, CSF1R, AXL, RAF1, DDR2*		[2, 16]
TAK-901	762151		*AURKA, AURKB, JAK3, SRC, CLK2, FGR, YES1, FGFR2*		[16]
CYC-116	759498		*AURKA, AURKB, KDR, FLT3, CDK2, CCNE1*		[2, 16]
ENMD-2076	758245		*FLT3, RET, AURKA, FLT4, CSF1R, FGFR3*		[16, 30]
AZD-4547	764239	BRD-K28392481	*FGFR1, FGFR2, FGFR3, FGFR4, IGF1R, KDR*		[1, 16]
PD-173074	766908		*FGFR1, FGFR1, KDR*		[11, 16]
BGJ-398	764487	NVP-BGJ398	*FGFR1, FGFR2, FGFR3, FGFR4, KDR*		[16]

Listed in the table are selected agents, their target genes, additional genes reported to contribute to sensitivity of each agent. For each agent listed in the table, we examined association of its response with the probe-specific and gene-specific methylation levels of the genes involved in the response mechanism and additional genes involved specificity or resistance of that agent. In addition, we examined association of each of the 44 agents listed in the table with methylation of individual probes and gene regions of 159 protein-coding genes that included genes involved in SCLC pathogenesis or lineage determination, SCLC lineage markers, genes that are frequently mutated, amplified, inactivated, or epigenetically modified in SCLC or in specific SCLC lineages, and genes involved in SCLC response to chemotherapy. These 159 genes included *RB1*, *TP53, PTEN, ASCL1*, *NEUROD1, YAP1, POU2F3, MYC, MYCN, MYCL1, BCL2*, *EZH2, SLFN11*, *PARP1*, *PARP2, ALK, IGF1*, *IGF2*, *IGF1R*, *IGFBP5, MTOR*, *EPAS1 (HIF2A)*, *TMEM127*, *KIF1B*, *KIF1C*, *KIF2A*, *KIF2B*, *KIF2C*, *KIF3A*, *KIF3B*, *KIF3C*, *KIF4A*, *KIF4B*, *KIF5A*, *KIF5B*, *KIF5C*, *KIF6*, *KIF7*, *KIF9*, *CENPE* (*KIF10*), *KIF11*, *KIF12*, *KIF13A*, *KIF13B*, *KIF14*, *KIF15*, *STARD9* (*KIF16A*), *KIF16B*, *KIF17*, *KIF18A*, *KIF18B*, *KIF19* (*KIF19A*), *KIF20A*, *KIF20B*, *KIF21A*, *KIF21B*, *KIF22*, *KIF23*, *KIF24*, *KIF25*, *KIF26A*, *KIF26B*, *KIFC1*, *KIFC2*, *KIFC3*, *PLK1*, *PLK2*, *PLK3*, *AURKA*, *AURKB*, *AURKC*, *FGFR1*, *FGFR2*, *FGFR3*, *FGFR4, AKT1, NEUROG2, OLIG2, NKX2-1, NKX2-2, NKX2-5, NKX6-1, HAND1, ZNF423, REST, HOXD1, HOXD3, HOXD4, HOXD8, HOXD9, HOXD10, HOXD11, HOXD12, HOXD13, FHIT, RASSF1* (*RASSF1A*), *TUSC2, SEMA3B, SEMA3F, CAV1, CREBBP, EP300, MLL, MLL2, TCF21, CCND1, TP73, KIAA1211, COL22A1, RGS7, FPR1, ASPM, ALMS1, PDE4DIP, XRN1, PTGFRN, RBL1, RBL2, FMN2, NOTCH1, NOTCH2, NOTCH3, NOTCH4, KIT, PIK3CA, CDKN2A, IRS2, CDK4, CDK6, MDM2, DLK1, HES1, HEY1, CHGA, GRP, SYP, NCAM1, CHD7, ASCL2, SOX9, DLL1, DLL3, KMT2D, CALCA, INSM1, TRPM5, SOX2, HES6, NFIB, SFTPC, KDM1A* (encoding LSD1), *GFI1B, CHEK1, WEE1, CD24, CADM1, ALCAM, CD151,* and *EPHA2*. For any gene which is included in this list of 159 genes, we analyzed associations of its methylation with response to each of the 44 antitumor agents listed in the table, even if that gene is listed in the table as being involved in response to specific drug agents.

References for Supplementary Table 2

1. Cancer Therapeutics Response Portal v. 2.0 Available from: https://portals.broadinstitute.org/ctrp/. Accessed: 5 July 2018
2. DrugBank v. 5.1.1. Available from: https://www.drugbank.ca. Accessed: 5 July 2018
3. Montecucco A, Zanetta F, Biamonti G. Molecular mechanisms of etoposide. EXCLI J. 2015;14:95-108.
4. Zoppoli G, Regairaz M, Leo E, Reinhold WC, Varma S, Ballestrero A et al. Putative DNA/RNA helicase Schlafen-11 (SLFN11) sensitizes cancer cells to DNA-damaging agents. Proc Natl Acad Sci U S A. 2012;109:15030-5.
5. Teicher BA. Next generation topoisomerase I inhibitors: Rationale and biomarker strategies. Biochem Pharmacol. 2008;75:1262-71.
6. Rees MG, Seashore-Ludlow B, Cheah JH, Adams DJ, Price EV, Gill S et al. Correlating chemical sensitivity and basal gene expression reveals mechanism of action. Nat Chem Biol. 2016;12:109-16.
7. Hodges LM, Markova SM, Chinn LW, Gow JM, Kroetz DL, Klein TE et al. Very important pharmacogene summary: ABCB1 (MDR1, P-glycoprotein). Pharmacogenet Genomics. 2011;21:152-61.
8. Pommier Y. Drugging topoisomerases: lessons and challenges. ACS Chem Biol. 2013;8:82-95.
9. Elnaggar M, Giovannetti E, Peters GJ. Molecular targets of gemcitabine action: rationale for development of novel drugs and drug combinations. Curr Pharm Des. 2012;18:2811-29.
10. Dewangan J, Srivastava S, Rath SK. Salinomycin: A new paradigm in cancer therapy. Tumour Biol. 2017;39:1010428317695035.
11. Genomics of Drug Sensitivity in Cancer. Available from: http://www.cancerrxgene.org/. Accessed: 5 July 2018
12. Yazinski SA, Comaills V, Buisson R, Genois MM, Nguyen HD, Ho CK et al. ATR inhibition disrupts rewired homologous recombination and fork protection pathways in PARP inhibitor-resistant BRCA-deficient cancer cells. Genes Dev. 2017;31:318-32.
13. Dziadkowiec KN, Gasiorowska E, Nowak-Markwitz E, Jankowska A. PARP inhibitors: review of mechanisms of action and BRCA1/2 mutation targeting. Prz Menopauzalny. 2016;15:215-9.
14. Underhill C, Toulmonde M, Bonnefoi H. A review of PARP inhibitors: from bench to bedside. Ann Oncol. 2011;22:268-79.
15. del Rivero J, Kohn EC. PARP Inhibitors: The Cornerstone of DNA Repair-Targeted Therapies. Oncology (Williston Park). 2017;31:265-73.
16. Selleck Chemicals online resource. Available from: http://www.selleckchem.com. Accessed: 5 July 2018
17. Oplustil O'Connor L, Rulten SL, Cranston AN, Odedra R, Brown H, Jaspers JE et al. The PARP Inhibitor AZD2461 Provides Insights into the Role of PARP3 Inhibition for Both Synthetic Lethality and Tolerability with Chemotherapy in Preclinical Models. Cancer Res. 2016;76:6084-94.
18. Wang J, Peng Y, Liu Y, Yang J, Huang M, Tan W. AT-101 inhibits hedgehog pathway activity and cancer growth. Cancer Chemother Pharmacol. 2015;76:461-9.
19. Houghton PJ. Everolimus. Clin Cancer Res. 2010;16:1368-72.
20. Carlo MI, Molina AM, Lakhman Y, Patil S, Woo K, DeLuca J et al. A Phase Ib Study of BEZ235, a Dual Inhibitor of Phosphatidylinositol 3-Kinase (PI3K) and Mammalian Target of Rapamycin (mTOR), in Patients With Advanced Renal Cell Carcinoma. Oncologist. 2016;21:787-8.
21. AbMole online drug information. Available from: http://www.abmole.com. Accessed: 10 September 2018
22. AdisInsight. Springer Nature Switzerland AG. Available from: https://adisinsight.springer.com. Accessed: 10 September 2018
23. Hollebecque A, Deutsch E, Massard C, Gomez-Roca C, Bahleda R, Ribrag V et al. A phase I, dose-escalation study of the Eg5-inhibitor EMD 534085 in patients with advanced solid tumors or lymphoma. Invest New Drugs. 2013;31:1530-8.
24. PubChem. Available from: https://pubchem.ncbi.nlm.nih.gov. Accessed: 20 August 2019
25. Hikichi Y, Honda K, Hikami K, Miyashita H, Kaieda I, Murai S et al. TAK-960, a novel, orally available, selective inhibitor of polo-like kinase 1, shows broad-spectrum preclinical antitumor activity in multiple dosing regimens. Mol Cancer Ther. 2012;11:700-9.
26. McLaughlin J, Markovtsov V, Li H, Wong S, Gelman M, Zhu Y et al. Preclinical characterization of Aurora kinase inhibitor R763/AS703569 identified through an image-based phenotypic screen. J Cancer Res Clin Oncol. 2010;136:99-113.
27. Sarno S, Shaw J, Spooner E, Ma J, Clark A, Dumontet C et al. The Novel Aurora Kinase Inhibitor AS703569 Shows Potent Anti-Tumor Activity in Acute Myeloid Leukemia (AML). Blood. 2007;110:915-.

28. Glaser KB, Li J, Marcotte PA, Magoc TJ, Guo J, Reuter DR et al. Preclinical characterization of ABT-348, a kinase inhibitor targeting the aurora, vascular endothelial growth factor receptor/platelet-derived growth factor receptor, and Src kinase families. J Pharmacol Exp Ther. 2012;343:617-27.
29. Basso AD, Liu M, Gray K, Tevar S, Lee S, Liang L et al. SCH 1473759, a novel Aurora inhibitor, demonstrates enhanced anti-tumor activity in combination with taxanes and KSP inhibitors. Cancer Chemother Pharmacol. 2011;68:923-33.
30. CASI Pharmaceuticals online information. Available from: http://www.casipharmaceuticals.com/. Accessed: 10 September 2018
