# Supplementary material for: Epigenome-wide DNA methylation analysis of small cell lung cancer cell lines suggests potential chemotherapy targets
Source: Clin Epigenetics. 2020 Jun 25;12:93. doi: 10.1186/s13148-020-00876-8 (PMC7318526; doi:10.1186/s13148-020-00876-8)
Supplement: Supplementary file 3 — Additional file 3: Supplementary Table 3. List of methylation probes with the strongest correlations with log(IC50) measure of drug response, which satisfied the Spearman correlation pO < 9.42 × 10^-8^ in the epigenome-wide analysis of all antitumor agents. [file 13148_2020_876_MOESM3_ESM.pdf]

Supplementary Table 3. List of methylation probes with the strongest correlations with log(IC50) measure of drug response, which satisfied the Spearman correlation $p_O < 9.42 \times 10^{-8}$ in the epigenome-wide analysis of all antitumor agents

NSC	Agent	Probe	Gene	Gene region	Chromosome	Cytoband	Genome position	Strand	Spearman ρ	*p*-value
763930	AS-703569	cg10821127			11	q14.3	91987297	R	0.6227	2.37×10^{-8}
763930	AS-703569	cg00113096	*NCKAP5*	Gene body	2	q21.2	134119805	F	0.6033	8.28×10^{-8}
763930	AS-703569	cg25038473	*IPCEF1*	Gene body	6	q25.2	154568414	R	0.6190	3.02×10^{-8}
758489	ZIP-301	cg01597882	*TRIM2*	5'UTR	4	q31.3	154077609	R	0.6406	6.93×10^{-9}
764043	(+)-JQ1	cg12947665			1	p34.1	45264237	R	-0.6086	5.91×10^{-8}
330507	17-AAG	cg20329958	*CRH*	5'UTR	8	q13.1	67090250	F	-0.6028	8.50×10^{-8}
759905	4SC-202	cg10986043	*TCAP*	TSS1500	17	q12	37820495	R	0.6101	5.40×10^{-8}
759905	4SC-202	cg13178916	*SNED1*	Gene body	2	q37.3	241976626	F	0.6927	1.16×10^{-10}
759659	ABT-263	cg25718735	*TCERG1L*	Gene body	10	q26.3	132959070	F	-0.6022	8.83×10^{-8}
765889	ABT-348	cg26295618	*CEP350*	3'UTR	1	q25.2	180080477	F	0.6096	5.54×10^{-8}
765889	ABT-348	cg19513950	*DISC1, TSNAX-DISC1*	Gene body, Gene body	1	q42.2	231799423	R	0.6260	1.90×10^{-8}
758873	ABT-737	cg23135199			4	p16.1	10171344	R	-0.6140	4.19×10^{-8}
758873	ABT-737	cg19058916	*LSM14A*	Gene body	19	q13.11	34668237	F	-0.6223	2.43×10^{-8}
758873	ABT-737	cg01404317	*SPG20*	5'UTR	13	q13.3	36919819	F	-0.6097	5.54×10^{-8}
758873	ABT-737	cg04652257			3	p14.3	55125730	R	-0.6047	7.55×10^{-8}
758873	ABT-737	cg12452800	*TCERG1L*	3'UTR	10	q26.3	132890896	F	-0.6283	2.11×10^{-8}
758873	ABT-737	cg10589396	*TCERG1L*	3'UTR	10	q26.3	132891341	F	-0.6310	1.35×10^{-8}
758873	ABT-737	cg20253807	*TCERG1L*	Gene body	10	q26.3	133000130	F	-0.6283	1.62×10^{-8}
758873	ABT-737	cg16996176	*TCERG1L*	Gene body	10	q26.3	133055651	F	-0.6058	7.07×10^{-8}
767746	ADW-742	cg17023289	*NPLOC4, NPLOC4, TSPAN10*	1st Exon, 5'UTR, TSS200	17	q25.3	79604034	F	0.6259	1.91×10^{-8}
750691	Afatinib	cg22208022	*ATF7IP*	5'UTR	12	p13.1	14553107	F	-0.6043	7.76×10^{-8}
767745	AG-2037	cg10784030	*INPP5B*	5'UTR, 1st Exon	1	p34.3	38412688	F	0.6013	9.32×10^{-8}
755774	AM-2282	cg10551296	*PSMB7*	Gene body	9	q33.3	127153520	F	-0.6034	8.20×10^{-8}
755774	AM-2282	cg19947214	*TRAPPC9*	Gene body	8	q24.3	141164218	R	-0.6069	6.58×10^{-8}
755774	AM-2282	cg08705382	*GALNT2*	Gene body	1	q42.13	230322024	F	-0.6157	3.75×10^{-8}
755774	AM-2282	cg01054140			2	q37.3	240736286	R	-0.6310	1.35×10^{-8}
761069	AMG-900	cg05333279			2	p24.1	20067114	R	0.6113	4.98×10^{-8}
761069	AMG-900	cg11314854			6	p21.1	41223577	F	0.6324	1.23×10^{-8}

761069	AMG-900	cg08937075	*EPAS1*	Gene body	2	p21	46541625	F	0.6278	1.68×10^{-8}
761069	AMG-900	cg13740187	*TPM3*	TSS200	1	q21.3	154164699	R	0.6225	2.40×10^{-8}
758242	ARQ-197	cg01597882	*TRIM2*	5'UTR	4	q31.3	154077609	R	0.6498	4.73×10^{-9}
754361	Astex FGF inhibitor	cg10789749	*ZCCHC3*	TSS1500	20	p13	276888	F	0.6261	1.88×10^{-8}
754361	Astex FGF inhibitor	cg01597882	*TRIM2*	5'UTR	4	q31.3	154077609	R	0.6580	1.94×10^{-9}
755762	AUY-922	cg18968627	*TBC1D9*	TSS1500	4	q31.21	141678207	R	-0.6127	4.57×10^{-8}
102816	Azacitidine	cg02841875			1	p36.31	6056063	R	0.6275	1.72×10^{-8}
102816	Azacitidine	cg05977840	*LIMK2*	5'UTR, 1st Exon, Gene body	22	q12.2	31644452	R	0.6164	3.58×10^{-8}
757444	AZD-1152	cg17631451	*TREX1*	5'UTR, 1st Exon	3	p21.31	48507354	R	-0.6126	4.58×10^{-8}
757444	AZD-1152	cg27340749	*TREX1*	5'UTR, 1st Exon	3	p21.31	48507385	R	-0.6093	5.68×10^{-8}
757444	AZD-1152	cg21750428	*MLPH*	3'UTR	2	q37.3	238462793	F	0.6148	3.97×10^{-8}
774901	AZD-2858	cg02509943	*COLEC12*	Gene body	18	p11.32	395186	F	-0.6026	8.65×10^{-8}
758871	AZD-8055	cg00808511	*TOX3;TOX3*	Gene body	16	q12.1	52495237	R	0.6291	1.54×10^{-8}
758871	AZD-8055	cg01873977	*MTSS1*	Gene body	8	q24.13	125699897	F	-0.6253	1.99×10^{-8}
764608	BAL-101553	cg02326883	*STARD3, TCAP*	3'UTR, TSS1500	17	q12	37820254	F	0.6283	1.63×10^{-8}
764608	BAL-101553	cg10986043	*TCAP*	TSS1500	17	q12	37820495	R	0.6080	6.16×10^{-8}
764608	BAL-101553	cg01597882	*TRIM2*	5'UTR	4	q31.3	154077609	R	0.6219	2.50×10^{-8}
764608	BAL-101553	cg13178916	*SNED1*	Gene body	2	q37.3	241976626	F	0.6092	5.69×10^{-8}
751249	BEZ-235	cg00808511	*TOX3*	Gene body	16	q12.1	52495237	R	0.6128	4.54×10^{-8}
755983	BI-2536	cg21750428	*MLPH*	3'UTR	2	q37.3	238462793	F	0.6172	3.41×10^{-8}
760842	BIM-46187	cg26332695			5	p15.31	6504885	F	-0.6046	7.63×10^{-8}
760842	BIM-46187	cg15816503	*C8orf74*	TSS200	8	p23.1	10530053	R	-0.6359	9.61×10^{-9}
760842	BIM-46187	cg14182145	*C8orf74*	TSS200	8	p23.1	10530083	F	-0.6514	3.16×10^{-9}
760842	BIM-46187	cg03790427	*C8orf74*	TSS200	8	p23.1	10530123	F	-0.6245	2.09×10^{-8}
760842	BIM-46187	cg00870242	*C8orf74*	5'UTR, 1st Exon	8	p23.1	10530148	F	-0.6721	6.47×10^{-10}
760842	BIM-46187	cg24413088			12	p13.1	12956269	F	-0.6064	6.82×10^{-8}
760842	BIM-46187	cg15562176	*ABCC8*	ExonBnd, Gene body	11	p15.1	17435030	R	-0.6139	4.22×10^{-8}
760842	BIM-46187	cg21164050			13	q12.13	27757411	F	-0.6645	1.17×10^{-9}
760842	BIM-46187	cg01568816	*SAPS2*	Gene body	22	q13.33	50876186	R	0.6190	3.03×10^{-8}
760842	BIM-46187	cg05019203	*BCAS1*	Gene body	20	q13.2	52612962	R	-0.6206	2.72×10^{-8}
760842	BIM-46187	cg08465708	*DAB1*	5'UTR	1	p32.2	57836927	R	-0.6057	7.10×10^{-8}
760842	BIM-46187	cg14877718			5	q13.2	71228754	R	-0.6072	6.47×10^{-8}

760842	BIM-46187	cg01777663	*KCNMA1*	Gene body	10	q22.3	79152870	F	-0.6066	6.71×10^{-8}
760842	BIM-46187	cg23669287	*NR2F2*	TSS1500, Gene body	15	q26.2	96873184	R	-0.6116	4.89×10^{-8}
760842	BIM-46187	cg04422798			14	q32.2	99624172	F	-0.6017	9.13×10^{-8}
760842	BIM-46187	cg19117938			6	q21	108449188	R	-0.6364	9.33×10^{-9}
760842	BIM-46187	cg20483936	*NDRG1*	TSS1500	8	q24.22	134310304	R	-0.6048	7.52×10^{-8}
760842	BIM-46187	cg16090801	*SLC19A2*	TSS1500	1	q24.2	169455894	F	-0.6059	7.00×10^{-8}
760842	BIM-46187	cg06062494	*SLC19A2*	TSS1500	1	q24.2	169455899	F	-0.6125	4.62×10^{-8}
760842	BIM-46187	cg21805731			1	q32.1	204560985	R	-0.6309	1.36×10^{-8}
754353	BKM-120	cg02375832	*C11orf48*	5'UTR	11	q12.3	62437615	R	0.6094	5.64×10^{-8}
125066	Bleomycin	cg04079260	*SETBP1*	Gene body	18	q12.3	42297128	R	0.6110	5.09×10^{-8}
764090	BMS-777607	cg17473712	*ROR1*	Gene body	1	p31.3	64287452	F	0.6026	8.61×10^{-8}
681239	Bortezamide	cg13731588	*MEI1*	Gene body	22	q13.2	42147558	F	-0.6143	4.11×10^{-8}
681239	Bortezamide	cg14802481	*MYO6*	Gene body	6	q14.1	76562746	R	-0.6115	4.91×10^{-8}
758485	BX-912	cg23518532			11	q24.3	128325015	R	0.6025	8.69×10^{-8}
761068	Cabozantinib	cg00558749			1	p36.33	1713950	R	-0.6344	1.07×10^{-8}
761068	Cabozantinib	cg10207218			1	p36.33	1714140	F	-0.6340	1.10×10^{-8}
761068	Cabozantinib	cg12492273	*MAD1L1*	Gene body	7	p22.3	2119499	F	0.6214	2.59×10^{-8}
761068	Cabozantinib	cg13225215			9	p23	13751009	F	0.6045	7.67×10^{-8}
761068	Cabozantinib	cg14264182			21	q22.12	36958638	F	0.6339	1.10×10^{-8}
761068	Cabozantinib	cg21467050	*MX1*	5'UTR, TSS1500	21	q22.3	42802842	F	0.6221	2.47×10^{-8}
761068	Cabozantinib	cg11798489			3	p21.31	46531037	R	0.6161	3.66×10^{-8}
761068	Cabozantinib	cg03140301			18	q21.1	47193110	R	0.6028	8.52×10^{-8}
761068	Cabozantinib	cg10901323	*SHROOM3*	Gene body	4	q21.1	77519000	F	0.6117	4.86×10^{-8}
761068	Cabozantinib	cg17775903			2	p11.2	85972061	R	0.6336	1.13×10^{-8}
761068	Cabozantinib	cg14999778	*CADPS2*	Gene body	7	q31.32	122024130	F	0.6214	2.57×10^{-8}
761068	Cabozantinib	cg22139166	*ZNF608*	Gene body	5	q23.2	124071107	F	0.6287	1.58×10^{-8}
761068	Cabozantinib	cg09361819	*ZNF608*	Gene body	5	q23.2	124071822	R	0.6102	5.36×10^{-8}
761068	Cabozantinib	cg01793651	*ZNF608*	Gene body	5	q23.2	124071885	F	0.6314	1.31×10^{-8}
761068	Cabozantinib	cg11614630	*FAM120B*	TSS1500, Gene body	6	q27	170602911	F	0.6207	2.70×10^{-8}
754350	CB-64D	cg21239432	*PVRL4*	Gene body	1	q23.3	161053211	F	-0.6113	4.97×10^{-8}
766824	Cephalon Early Alk Inhibitor	cg09278322	*GCA*	TSS1500	2	q24.2	163199399	R	0.6047	7.55×10^{-8}
606869	Clofarabine	cg22705835	*REEP3*	Gene body	10	q21.3	65332833	R	-0.6116	4.89×10^{-8}

762419	CT-32228	cg07992385	*STK39*	Gene body	2	q24.3	169058521	F	0.6620	1.43×10^{-9}
771751	CUDC-907	cg27295742	*RNMTL1*	Gene body	17	p13.3	693324	R	0.6102	5.33×10^{-8}
771751	CUDC-907	cg20663203	*VWF*	Gene body	12	p13.31	6202393	F	-0.6016	9.19×10^{-8}
771751	CUDC-907	cg23162571			7	q11.21	65196762	R	0.6335	1.14×10^{-8}
758488	CYT-997	cg05011532			2	p22.2	38049921	F	0.6244	2.11×10^{-8}
82151	Daunorubicin	cg22963554	*KIAA1024*	TSS200	15	q25.1	79724739	F	0.6190	3.02×10^{-8}
754143	Depsipeptide	cg20640611	*VWF*	Gene body	12	p13.31	6106486	F	-0.6535	2.70×10^{-9}
754143	Depsipeptide	cg00301124	*VWF*	Gene body	12	p13.31	6142916	F	-0.6331	1.17×10^{-8}
754143	Depsipeptide	cg11867485	*VWF*	Gene body	12	p13.31	6142941	F	-0.6384	8.10×10^{-9}
754143	Depsipeptide	cg03208825	*VWF*	Gene body	12	p13.31	6178792	F	-0.6163	3.60×10^{-8}
754143	Depsipeptide	cg02498227	*VWF*	Gene body	12	p13.31	6193950	F	-0.6229	2.34×10^{-8}
754143	Depsipeptide	cg15931460	*VWF*	Gene body	12	p13.31	6206659	F	-0.6499	3.54×10^{-9}
754143	Depsipeptide	cg13457204			3	p24.1	30569452	R	-0.6020	8.98×10^{-8}
754143	Depsipeptide	cg03594803	*IDO2*	Gene body	8	p11.21	39809404	F	-0.6248	2.07×10^{-8}
754143	Depsipeptide	cg19355078	*MAST4*	Gene body	5	q12.3	65957229	F	-0.6339	1.10×10^{-8}
754143	Depsipeptide	cg24768430			12	q21.1	71999138	R	-0.6036	8.11×10^{-8}
754143	Depsipeptide	cg22771548	*RGNEF*	Gene body	5	q13.2	73044623	F	-0.6119	4.79×10^{-8}
754143	Depsipeptide	cg13002526			16	q23.2	79746407	R	-0.6036	8.08×10^{-8}
754143	Depsipeptide	cg19131143	*DYNLRB2, LOC102724084*	TSS1500, Gene body	16	q23.2	80573443	F	-0.6038	7.99×10^{-8}
754143	Depsipeptide	cg02052531			5	q14.2	81692895	R	-0.6605	1.59×10^{-9}
754143	Depsipeptide	cg24064300			8	q22.3	102405422	F	-0.6218	2.52×10^{-8}
754143	Depsipeptide	cg00093956	*MTERFD3*	Gene body	12	q23.3	107372431	F	-0.6468	4.43×10^{-9}
754143	Depsipeptide	cg08114265	*FYN*	5'UTR	6	q21	112052356	R	-0.6490	3.78×10^{-9}
754143	Depsipeptide	cg05270106	*EEFSEC*	Gene body	3	q21.3	127997992	F	-0.6201	2.81×10^{-8}
754143	Depsipeptide	cg06959135	*AHSG*	TSS200	3	q27.3	186330804	R	-0.6374	8.66×10^{-9}
754143	Depsipeptide	cg24583966	*KISS1*	TSS1500	1	q32.1	204166965	R	-0.6296	1.48×10^{-8}
95100	Digoxin	cg08758345	*KLHL26*	Gene body	19	p13.11	18770246	F	0.6606	1.58×10^{-9}
95100	Digoxin	cg27449660			18	q21.1	45938918	F	0.6027	8.60×10^{-8}
95100	Digoxin	cg24867453	*SLC16A6, ARSG*	TSS1500, 5'UTR	17	q24.2	66288531	F	0.6389	7.83×10^{-9}
95100	Digoxin	cg17488628	*MARK1*	Gene body	1	q41	220717700	F	0.6285	1.60×10^{-8}
95100	Digoxin	cg18637746	*CUL3*	TSS1500;Gene body	2	q36.2	225435124	R	0.6092	5.69×10^{-8}
95100	Digoxin	cg01474011	*CUL3*	Gene body	2	q36.2	225435131	F	0.6093	5.66×10^{-8}

123127	Doxorubicin	cg22595778			10	p13	15484150	R	-0.6357	9.79×10^{-9}
123127	Doxorubicin	cg21582973	*GPX6*	Gene body	6	p22.1	28478268	R	-0.6023	8.78×10^{-8}
123127	Doxorubicin	cg05067701	*NAALADL2-AS2;NAALADL2*	Gene body, Gene body	3	q26.31	174976757	F	-0.6058	7.05×10^{-8}
758245	ENMD-2076	cg10789749	*ZCCHC3*	TSS1500	20	p13	276888	F	0.6341	1.09×10^{-8}
758245	ENMD-2076	cg12045634	*NRP1*	Gene body	10	p11.22	33491867	R	0.6566	2.14×10^{-9}
758245	ENMD-2076	cg11314854			6	p21.1	41223577	F	0.6397	7.36×10^{-9}
758245	ENMD-2076	cg26430059	*FOXN3*	Gene body	14	q31.3	89770384	R	-0.6500	3.52×10^{-9}
758245	ENMD-2076	cg12144100	*PTPRN2*	Gene body	7	q36.3	157890277	R	0.6092	5.70×10^{-8}
649890	Flavopiridol	cg14597426			22	q11.23	24113481	R	0.6036	8.11×10^{-8}
649890	Flavopiridol	cg09900893	*RPS6KA1*	Gene body	1	p36.11	26865376	F	0.6152	3.88×10^{-8}
649890	Flavopiridol	cg06854842	*KCNK3*	Gene body	2	p23.3	26947187	R	0.6145	4.07×10^{-8}
649890	Flavopiridol	cg00965154	*SMTN*	Gene body	22	q12.2	31485117	R	0.6294	1.51×10^{-8}
649890	Flavopiridol	cg17498965	*MAPRE2*	TSS1500, 5'UTR, Gene body	18	q12.1	32619901	R	0.6209	2.68×10^{-8}
649890	Flavopiridol	cg11188119	*LSM12*	TSS1500	17	q21.31	42145270	R	0.6124	4.64×10^{-8}
649890	Flavopiridol	cg11818720	*CACNA2D2*	Gene body	3	p21.31	50464174	F	0.6098	5.47×10^{-8}
649890	Flavopiridol	cg12009872	*CYP19A1;CYP19A1*	Gene body	15	q21.2	51520739	R	0.6360	9.54×10^{-9}
649890	Flavopiridol	cg03823125	*SUV420H1*	5'UTR	11	q13.2	67968226	F	0.6027	8.59×10^{-8}
649890	Flavopiridol	cg19757771	*NCALD*	5'UTR	8	q22.3	102768682	R	0.6188	3.07×10^{-8}
649890	Flavopiridol	cg13912824	*PTPRN2*	Gene body	7	q36.3	157346224	R	0.6120	4.76×10^{-8}
649890	Flavopiridol	cg16328225	*ATP13A3*	Gene body	3	q29	194171286	R	-0.6434	5.68×10^{-9}
649890	Flavopiridol	cg18240528			1	q32.1	205341305	R	0.6174	3.36×10^{-8}
764091	GDC-0980	cg05764519			1	p22.3	88151396	F	0.6045	7.66×10^{-8}
764091	GDC-0980	cg27501748	*MAP3K5*	Gene body	6	q23.3	136929561	R	0.6221	2.47×10^{-8}
768112	GENZ-644282	cg06972794	*LHX4*	Gene body	1	q25.2	180205268	F	0.6022	8.85×10^{-8}
764092	GSK-2126458	cg03359704			6	p25.1	6694733	R	0.6086	5.91×10^{-8}
764092	GSK-2126458	cg11223753	*TBC1D22A*	Gene body	22	q13.31	47525730	R	0.6112	5.03×10^{-8}
764092	GSK-2126458	cg09693811			5	q13.3	73553751	F	0.6161	3.65×10^{-8}
754354	GSK-461364	cg12045634	*NRP1*	Gene body	10	p11.22	33491867	R	0.6367	9.09×10^{-9}
764658	INK-128	cg23964820	*NRG1*	Gene body	8	p12	32076032	F	0.6089	5.81×10^{-8}
764658	INK-128	cg20737582			6	q21	112942289	R	0.6014	9.29×10^{-8}
764658	INK-128	cg27501748	*MAP3K5*	Gene body	6	q23.3	136929561	R	0.6018	9.06×10^{-8}
772595	KPT-185	cg25731731	*LINC00598*	Gene body	13	q14.11	41019891	R	-0.6186	3.10×10^{-8}

773260	KPT-251	cg25731731	*LINC00598*	Gene body	13	q14.11	41019891	R	-0.6067	6.66×10^{-8}
756643	KX2-391	cg01597882	*TRIM2*	5'UTR	4	q31.3	154077609	R	0.6082	6.07×10^{-8}
26980	Mitomycin C	cg19254152	*C7orf50*	Gene body	7	p22.3	1083473	F	0.6167	3.52×10^{-8}
279836	Mitoxantrone	cg05260411			11	q13.2	67331241	F	-0.6088	5.84×10^{-8}
279836	Mitoxantrone	cg12438215	*ALDH3B2*	5'UTR	11	q13.2	67441397	R	-0.6168	3.50×10^{-8}
767470	MLN-0905	cg17298776	*ROR1*	Gene body	1	p31.3	64242960	F	0.6032	8.29×10^{-8}
767470	MLN-0905	cg26195586	*SIL1*	5'UTR	5	q31.2	138532335	R	0.6093	5.68×10^{-8}
759677	MLN-8237	cg10789749	*ZCCHC3*	TSS1500	20	p13	276888	F	0.6052	7.32×10^{-8}
759677	MLN-8237	cg14680496	*MAD1L1*	Gene body	7	p22.3	2119663	F	0.6348	1.04×10^{-8}
758254	MLN-9708	cg13713718	*NOC2L, KLHL17*	TSS1500, TSS200	1	p36.33	895864	F	-0.6055	7.21×10^{-8}
758254	MLN-9708	cg14935926			12	q24.11	111423358	F	-0.6115	4.94×10^{-8}
36405	MP-0922	cg16239536	*HMHA1*	Gene body	19	p13.3	1079617	F	0.6128	4.53×10^{-8}
36405	MP-0922	cg02324006	*HMHA1*	Gene body	19	p13.3	1080034	R	0.6039	7.97×10^{-8}
754349	MP-470	cg06399596			6	p25.3	1595676	R	0.6109	5.12×10^{-8}
765395	MPC-3100	cg11545923	*GABRG3*	Gene body	15	q12	27272330	R	0.6078	6.22×10^{-8}
765395	MPC-3100	cg07477792			3	q13.32	117716071	F	-0.6089	5.82×10^{-8}
753686	Olaparib	cg14989164	*LHX4*	Gene body	1	q25.2	180205157	F	0.6309	1.36×10^{-8}
753686	Olaparib	cg06972794	*LHX4*	Gene body	1	q25.2	180205268	F	0.6559	2.26×10^{-9}
754344	ON-01910Na	cg04486382	*MSI2*	Gene body	17	q22	55483894	R	0.6041	7.84×10^{-8}
764764	ONX-0801	cg01249670			3	p13	71707453	R	-0.6142	4.13×10^{-8}
766254	ONX-0912	cg19326844			3	p25.3	10754083	R	-0.6076	6.31×10^{-8}
766254	ONX-0912	cg21253620	*s*	Gene body	3	p25.3	10890374	F	-0.6180	3.24×10^{-8}
766254	ONX-0912	cg26408837	*RBFOX3*	5'UTR	17	q25.3	77334776	R	-0.6235	2.24×10^{-8}
766254	ONX-0912	cg25242907	*RBFOX3*	5'UTR	17	q25.3	77336729	F	-0.6058	7.06×10^{-8}
766254	ONX-0912	cg10920336	*RBFOX3*	5'UTR	17	q25.3	77469302	F	-0.6030	8.43×10^{-8}
766254	ONX-0912	cg09001689	*RBFOX3*	5'UTR	17	q25.3	77470008	R	-0.6158	3.73×10^{-8}
766254	ONX-0912	cg14587335	*HRNBP3*	TSS200	17	q25.3	77478600	R	-0.6368	9.06×10^{-9}
766254	ONX-0912	cg20214871	*HRNBP3*	TSS200	17	q25.3	77478637	F	-0.6121	4.73×10^{-8}
766254	ONX-0912	cg02164741			17	q25.3	77537018	R	-0.6141	4.17×10^{-8}
766254	ONX-0912	cg15847600	*CYP11B2*	Gene body	8	q24.3	143995000	R	-0.6078	6.21×10^{-8}
766254	ONX-0912	cg11033617	*RASGEF1C*	Gene body	5	q35.3	179562118	R	-0.6101	5.39×10^{-8}
761190	Panobinostat	cg06134905			1	p22.3	85779844	R	-0.6094	5.63×10^{-8}

764136	PCI-24781	cg20663203	*VWF*	Gene body	12	p13.31	6202393	F	-0.6271	1.76×10^{-8}
764136	PCI-24781	cg02444978	*ISPD*	Gene body	7	p21.2	16438128	R	-0.6103	5.30×10^{-8}
698037	Pemetrexed	cg09186051	*DISC1, TSNAX-DISC1*	Gene body, Gene body	1	q42.2	231981906	F	0.6142	4.15×10^{-8}
760536	PF-04691502	cg01873977	*MTSS1*	Gene body	8	q24.13	125699897	F	-0.6043	7.78×10^{-8}
768509	PF-04929113	cg02380531			6	q16.1	99279097	F	-0.6216	2.55×10^{-8}
768509	PF-04929113	cg17303756			6	q16.1	99279108	F	-0.6193	2.97×10^{-8}
768509	PF-04929113	cg24449777			6	q16.1	99279483	R	-0.6432	5.74×10^{-9}
758256	PF-05212384	cg06010020	*LOC389458*	Gene body	7	p22.1	5112067	F	0.6101	5.39×10^{-8}
754356	PF-562271	cg11314854			6	p21.1	41223577	F	0.6307	1.38×10^{-8}
754356	PF-562271	cg19546158	*CORO1C*	Gene body	12	q24.11	109084984	R	-0.6025	8.69×10^{-8}
766271	PHA-665752	cg26806588	*LINC01359*	Gene body	1	p31.3	65446986	R	-0.6105	5.24×10^{-8}
766271	PHA-665752	cg24891434	*ANO1*	Gene body	11	q13.3	69934031	R	-0.6111	5.04×10^{-8}
170984	Pimozide	cg05861705			6	q14.1	82417175	F	0.6017	9.12×10^{-8}
170984	Pimozide	cg11235712			6	q27	169273049	F	0.6253	1.99×10^{-8}
24559	Plicamycin	cg21208806	*ARPC5, RGL1*	TSS1500, 5'UTR	1	q25.3	183605960	F	-0.6237	2.22×10^{-8}
758487	Ponatinib	cg24146218			3	p24.1	30398133	F	0.6529	2.82×10^{-9}
758487	Ponatinib	cg05505221			4	q34.3	182088029	F	0.6039	7.95×10^{-8}
758487	Ponatinib	cg13577629			4	q34.3	182102403	F	0.6029	8.46×10^{-8}
754230	Pralatrexate	cg27455540			2	p24.2	18866125	R	0.6111	5.04×10^{-8}
757296	Pyrvinium pamoate	cg04263702	*FBXL18*	Gene body	7	p22.1	5528266	F	0.6612	1.51×10^{-9}
760444	R-115777	cg18341491	*POSTN*	TSS1500	13	q13.3	38174258	F	0.6228	2.36×10^{-8}
765262	R-428	cg20353571	*LIMCH1*	Gene body	4	p13	41694962	F	0.6150	3.93×10^{-8}
773094	R-547	cg11755201	*CAMTA1*	Gene body	1	p36.23	7551851	F	-0.6267	1.81×10^{-8}
773094	R-547	cg13792566	*PAN3*	Gene body	13	q12.2	28735807	F	0.6075	6.34×10^{-8}
773094	R-547	cg12045634	*NRP1*	Gene body	10	p11.22	33491867	R	0.6139	4.22×10^{-8}
773094	R-547	cg14360917	*SP2*	Gene body	17	q21.32	45992122	F	0.6407	6.88×10^{-9}
773094	R-547	cg17631451	*TREX1*	5'UTR, 1st Exon	3	p21.31	48507354	R	-0.6135	4.34×10^{-8}
773094	R-547	cg27340749	*TREX1*	5'UTR, 1st Exon	3	p21.31	48507385	R	-0.6674	9.39×10^{-10}
773094	R-547	cg26029997	*TREX1*	5'UTR, 1st Exon	3	p21.31	48507610	F	-0.6428	5.91×10^{-9}
773094	R-547	cg19091784	*TREX1*	5'UTR, 1st Exon	3	p21.31	48507618	F	-0.6312	1.33×10^{-8}
773094	R-547	cg15358372	*HK1*	Gene body	10	q22.1	71108752	F	-0.6025	8.69×10^{-8}
773094	R-547	cg03856178	*ARID5A*	TSS200	2	q11.2	97202314	R	0.6128	4.52×10^{-8}

773094	R-547	cg23518532			11	q24.3	128325015	R	0.6099	5.45×10^{-8}
773094	R-547	cg09805466	*MOGAT1*	Gene body	2	q36.1	223566483	F	0.6201	2.81×10^{-8}
773094	R-547	cg27210286	*GIGYF2*	TSS1500	2	q37.1	233561361	F	0.6424	6.09×10^{-9}
771532	RG-7603	cg05538359			17	p12	10762683	R	-0.6031	8.34×10^{-8}
762673	SB-1317	cg17435483			7	q33	135709877	F	0.6025	8.68×10^{-8}
754362	SB-590885	cg20930706	*LOH12CR1;LOH12CR1*	Gene body	12	p13.2	12595583	F	-0.6364	9.31×10^{-9}
763525	SC-1	cg22264616	*WT1*	3'UTR	11	p13	32410090	F	-0.6372	8.77×10^{-9}
763525	SC-1	cg09883602	*WT1*	Gene body	11	p13	32415339	F	-0.6377	8.50×10^{-9}
763525	SC-1	cg19211915	*WT1*	Gene body	11	p13	32452513	F	-0.6159	3.71×10^{-8}
763525	SC-1	cg09612493			11	p13	32495732	F	-0.6393	7.59×10^{-9}
763525	SC-1	cg06773584	*UNC45B*	Gene body	17	q12	33511632	R	-0.6082	6.06×10^{-8}
763525	SC-1	cg20079642			22	q13.31	45041269	R	-0.6196	2.90×10^{-8}
763525	SC-1	cg02785222			3	q23	138892919	R	-0.6158	3.72×10^{-8}
761691	SCH-1473759	cg20018344	*ITPA*	3'UTR	20	p13	3204138	R	-0.6110	5.09×10^{-8}
761691	SCH-1473759	cg13202325			17	p11.2	17320004	F	-0.6041	7.86×10^{-8}
761691	SCH-1473759	cg27340749	*TREX1*	5'UTR, 1st Exon	3	p21.31	48507385	R	-0.6114	4.95×10^{-8}
761691	SCH-1473759	cg02562900			18	q23	74061002	R	-0.6367	9.14×10^{-9}
761691	SCH-1473759	cg23677426	*TBC1D8*	Gene body	2	q11.2	101727028	F	-0.6128	4.54×10^{-8}
761691	SCH-1473759	cg00813377			3	q21.3	127261421	R	-0.6102	5.35×10^{-8}
761691	SCH-1473759	cg19947214	*TRAPPC9*	Gene body	8	q24.3	141164218	R	-0.6018	9.09×10^{-8}
761691	SCH-1473759	cg08705382	*GALNT2*	Gene body	1	q42.13	230322024	F	-0.6244	2.12×10^{-8}
633782	Simvastatin	cg06228260	*PTPRN2*	Gene body	7	q36.3	157854729	R	0.6063	6.87×10^{-8}
758250	SNS-314	cg13168274			18	p11.31	5288604	F	0.6399	7.26×10^{-9}
758250	SNS-314	cg12045634	*NRP1*	Gene body	10	p11.22	33491867	R	0.6046	7.62×10^{-8}
758250	SNS-314	cg04606096	*ERG*	5'UTR, Gene body	21	q22.2	39848437	F	0.6102	5.36×10^{-8}
758250	SNS-314	cg01870865	*TREX1*	TSS200	3	p21.31	48507087	R	-0.6276	1.71×10^{-8}
758250	SNS-314	cg08093733	*MBP;MBP*	Gene body	18	q23	74754635	R	0.6277	1.70×10^{-8}
758250	SNS-314	cg23518532			11	q24.3	128325015	R	0.6334	1.15×10^{-8}
758250	SNS-314	cg13740187	*TPM3*	TSS200	1	q21.3	154164699	R	0.6507	3.34×10^{-9}
758250	SNS-314	cg26295618	*CEP350*	3'UTR	1	q25.2	180080477	F	0.6280	1.66×10^{-8}
762151	TAK-901	cg13202325			17	p11.2	17320004	F	-0.6102	5.34×10^{-8}
762151	TAK-901	cg12045634	*NRP1*	Gene body	10	p11.22	33491867	R	0.6050	7.44×10^{-8}

762151	TAK-901	cg01870865	*TREX1*	TSS200	3	p21.31	48507087	R	-0.6195	2.93×10^{-8}
762151	TAK-901	cg17631451	*TREX1*	5'UTR, 1st Exon	3	p21.31	48507354	R	-0.6346	1.05×10^{-8}
762151	TAK-901	cg27340749	*TREX1*	5'UTR, 1st Exon	3	p21.31	48507385	R	-0.6457	4.81×10^{-9}
762151	TAK-901	cg26029997	*TREX1*	5'UTR, 1st Exon	3	p21.31	48507610	F	-0.6077	6.27×10^{-8}
762151	TAK-901	cg19091784	*TREX1*	5'UTR, 1st Exon	3	p21.31	48507618	F	-0.6094	5.63×10^{-8}
762151	TAK-901	cg02562900			18	q23	74061002	R	-0.6386	7.98×10^{-9}
762151	TAK-901	cg23518532			11	q24.3	128325015	R	0.6028	8.51×10^{-8}
762151	TAK-901	cg08705382	*GALNT2*	Gene body	1	q42.13	230322024	F	-0.6163	3.61×10^{-8}
768072	TAK-960	cg16371803	*SNX29*	Gene body	16	p13.13	12359260	R	0.6280	1.66×10^{-8}
768072	TAK-960	cg24947024	*RNF8*	Gene body	6	p21.2	37324995	F	0.6073	6.43×10^{-8}
768072	TAK-960	cg06741989	*PDGFB*	TSS1500	22	q13.1	39641872	F	0.6098	5.50×10^{-8}
768072	TAK-960	cg22433726	*TRIM47*	3'UTR	17	q25.1	73870495	R	0.6025	8.68×10^{-8}
768072	TAK-960	cg02902415			1	p13.3	110088541	F	0.6171	3.44×10^{-8}
768072	TAK-960	cg19701266	*ARHGEF12*	Gene body	11	q23.3	120217032	R	0.6262	1.87×10^{-8}
761759	TAK-960 analog	cg03396939			17	p11.2	19899956	R	0.6017	9.14×10^{-8}
761759	TAK-960 analog	cg19251326	*LY6G6C*	Gene body	6	p21.33	31688080	F	0.6041	7.86×10^{-8}
761759	TAK-960 analog	cg27588348			11	q12.1	58690510	R	0.6047	7.57×10^{-8}
761759	TAK-960 analog	cg22977072	*RAC3*	Gene body	17	q25.3	79991680	F	0.6041	7.87×10^{-8}
761759	TAK-960 analog	cg19701266	*ARHGEF12*	Gene body	11	q23.3	120217032	R	0.6140	4.18×10^{-8}
761759	TAK-960 analog	cg06711837	*FGF13*	Gene body	X	q26.3	137792814	R	0.6396	7.42×10^{-9}
180973	Tamoxifen	cg06661671	*CABIN1*	5'UTR	22	q11.23	24407852	R	0.6510	3.26×10^{-9}
9706	Triethylenemelamine	cg06972794	*LHX4*	Gene body	1	q25.2	180205268	F	0.6013	9.37×10^{-8}
761693	Vertex ATR inhibitor Cpd 45	cg27340749	*TREX1*	5'UTR, 1st Exon	3	p21.31	48507385	R	-0.6135	4.33×10^{-8}
761693	Vertex ATR inhibitor Cpd 45	cg26029997	*TREX1*	5'UTR, 1st Exon	3	p21.31	48507610	F	-0.6273	1.74×10^{-8}
761693	Vertex ATR inhibitor Cpd 45	cg19091784	*TREX1*	5'UTR, 1st Exon	3	p21.31	48507618	F	-0.6124	4.64×10^{-8}
49842	Vinblastine	cg02326883	*STARD3, TCAP*	3'UTR, TSS1500	17	q12	37820254	F	0.6345	1.06×10^{-8}
49842	Vinblastine	cg10986043	*TCAP*	TSS1500	17	q12	37820495	R	0.6209	2.67×10^{-8}
49842	Vinblastine	cg13178916	*SNED1*	Gene body	2	q37.3	241976626	F	0.6043	7.77×10^{-8}
608210	Vinorelbine	cg26029997	*TREX1*	5'UTR, 1st Exon	3	p21.31	48507610	F	-0.6081	6.11×10^{-8}
757437	VS-507	cg15998609	*ESRRG*	Gene body	1	q41	216774956	F	-0.6025	8.66×10^{-8}
755981	WP-1034	cg04603419	*PYY*	5'UTR	17	q21.31	42061560	R	-0.6198	2.87×10^{-8}

755927	WZ-4002	cg16808946	*USH2A*	Gene body	1	q41	215884009	R	-0.6015	9.24×10^{-8}
755775	XL-880	cg05333279			2	p24.1	20067114	R	0.6206	2.72×10^{-8}
772351	XL-888	cg16032803	*ETV6, RNU6-19P*	Gene body, Gene body	12	p13.2	12009167	R	-0.6474	4.25×10^{-9}
772351	XL-888	cg02380531			6	q16.1	99279097	F	-0.6041	7.88×10^{-8}
772351	XL-888	cg24449777			6	q16.1	99279483	R	-0.6245	2.10×10^{-8}
754366	XR-5944	cg04022401	*TTI1*	Gene body	20	q11.23	36621383	F	0.6269	1.78×10^{-8}
754366	XR-5944	cg23831735	*CCNA1*	TSS1500	13	q13.3	37004787	F	0.6051	7.38×10^{-8}
754366	XR-5944	cg11028313	*PLCD3, ACBD4*	Gene body, TSS1500	17	q21.31	43209292	R	0.6067	6.68×10^{-8}
754366	XR-5944	cg11926063	*RAB7A*	TSS1500	3	q21.3	128444746	R	0.6126	4.60×10^{-8}
765436	YK-4-279	cg15685549	*FLJ22447*	Gene body	14	q23.1	62069891	F	0.6094	5.64×10^{-8}
765436	YK-4-279	cg01597882	*TRIM2*	5'UTR	4	q31.3	154077609	R	0.6426	6.00×10^{-9}

Listed are methylation probes that passed the quality control and SNP filtering and were associated with drug response ($p < 9.42 \times 10^{-8}$). Gene and gene region annotation is provided according to Illumina EPIC array annotation according to the UCSC genome browser annotation. If a probe was annotated to be in multiple regions of a gene due to multiple splicing, all annotated gene regions are listed. If a probe was annotated as belonging to more than one gene, the gene regions for each gene are listed in the same order as the different genes for that probe.

TSS1500, 200–1500 bases upstream of the transcriptional start site (TSS); **TSS200**, 0–200 bases upstream of TSS; **5’UTR**, within the 5' untranslated region, between the TSS and the ATG start site; **1st Exon**, first exon; **ExonBnd**, Exon boundary; **Gene body**, Body of the gene (between the ATG start site and the stop codon); **3’UTR**, within the 3’ untranslated region, between the stop codon and poly A signal.

R, reverse genome strand; **F,** forward genome strand
