# Supplementary material for: Epigenome-wide DNA methylation analysis of small cell lung cancer cell lines suggests potential chemotherapy targets
Source: Clin Epigenetics. 2020 Jun 25;12:93. doi: 10.1186/s13148-020-00876-8 (PMC7318526; doi:10.1186/s13148-020-00876-8)
Supplement: Supplementary file 10 — Additional file 10: Supplementary Data 2. A. Associations of methylation beta-values of individual probes in the SNED1 gene with log(IC50) of the HDAC inhibitor 4SC-202 (NSC 759905). B. Associations of methylation beta-values of individual probes in SNED1 with log(IC50) of the HDAC inhibitor vorinostat (NSC 701852). C. Associations of SNED1 transcript expression with response to HDAC inhibitors. D. Additional information about associations of probes in selected genes. [file 13148_2020_876_MOESM10_ESM.pdf]

Supplementary Data 2

A. Associations of methylation beta-values of individual probes in the *SNED1* gene with log(IC50) of the HDAC inhibitor 4SC-202 (NSC 759905)

Probe	Spearman ρ	p_O	Gene annotation according to the UCSC Genome Browser
**cg13178916**	**0.6927**	$\mathbf{1.16 \times 10^{-10}}$	***SNED1***
cg06127335	0.5390	3.03×10^{-6}	*SNED1*
cg07644939	0.5091	1.27×10^{-5}	*SNED1*
cg03087247	0.5073	1.38×10^{-5}	*SNED1*
cg10717312	0.5007	1.86×10^{-5}	*SNED1*
cg17053285	0.4961	2.28×10^{-5}	*SNED1*
cg26718213	0.4938	2.52×10^{-5}	*SNED1*
cg19075225	0.4530	0.0001	*SNED1*
cg22635676	0.4424	0.0002	*SNED1*
cg26707709	0.4287	0.0003	*SNED1*
cg01121603	0.4049	0.0007	*SNED1*
cg02743576	0.4028	0.0008	*SNED1*
cg18624016	0.4008	0.0009	*SNED1*
cg09991306	0.3963	0.0010	*SNED1*
cg11207793	0.3939	0.0011	*SNED1*
cg05854427	0.3932	0.0011	*SNED1*
cg24401641	0.3927	0.0011	*SNED1*
cg04481534	0.3909	0.0012	*SNED1*
cg04398322	0.3873	0.0013	*SNED1*
cg08400319	0.3796	0.0017	*SNED1*
cg15120952	0.3784	0.0017	*SNED1*
cg09409209	0.3784	0.0017	*SNED1*
cg25850181	0.3748	0.0019	*SNED1*
cg14767619	0.3697	0.0022	*SNED1*
cg14485214	0.3601	0.0030	*SNED1*
cg06741256	0.3564	0.0033	*SNED1*
cg17685017	0.3527	0.0037	*SNED1*
cg23492351	0.3525	0.0037	*SNED1*
cg04372536	0.3503	0.0039	*SNED1*
cg09028445	0.3470	0.0043	*SNED1*
cg03915707	0.3465	0.0044	*SNED1;SNED1*
cg26464998	0.3437	0.0047	*SNED1*
cg13206794	0.3433	0.0048	*SNED1*
cg01661844	0.3433	0.0048	*SNED1*
cg12654776	0.3416	0.0050	*SNED1*
cg26799999	0.3415	0.0050	*SNED1*
cg02225728	0.3415	0.0050	*SNED1*
cg19390934	0.3402	0.0052	*MTERFD2;SNED1;MTERFD2;MTERFD2*
cg13850887	0.3397	0.0053	*SNED1*

cg24912802	0.3394	0.0053	*SNED1*
cg07098762	0.3302	0.0068	*SNED1*
cg14877755	0.3281	0.0072	*SNED1*
cg08565567	0.3208	0.0086	*SNED1*
cg07834788	0.3197	0.0089	*SNED1*
cg08557757	0.2978	0.0152	*SNED1*
cg02098967	0.2886	0.0188	*SNED1*
cg05812899	0.2861	0.0199	*SNED1*
cg12047959	0.2848	0.0204	*SNED1*
cg15396252	0.2838	0.0209	*SNED1*
cg26058474	0.2828	0.0214	*SNED1*
cg06044455	0.2715	0.0274	*SNED1;SNED1*
cg03432919	0.2697	0.0285	*SNED1*
cg18467041	0.2583	0.0363	*SNED1*
cg18663232	0.2541	0.0395	*MTERFD2;MTERFD2;SNED1;MTERFD2*
cg01305291	0.2530	0.0404	*SNED1*
cg15361291	0.2478	0.0448	*SNED1*
cg19892115	0.2455	0.0469	*MTERFD2;MTERFD2;SNED1;MTERFD2*
cg14359606	0.2424	0.0499	*SNED1*
cg12964210	0.2363	0.0561	*SNED1*
cg19322479	0.2316	0.0614	*SNED1*
cg11642880	0.2178	0.0789	*SNED1*
cg21239079	0.2151	0.0829	*SNED1;SNED1*
cg25241559	0.2120	0.0875	*SNED1*
cg10314439	0.2105	0.0899	*SNED1*
cg14574514	0.2075	0.0946	*SNED1*
cg07612300	-0.2045	0.0995	*MTERFD2;MTERFD2;SNED1;MTERFD2*
cg21773665	0.1936	0.1194	*MTERFD2;SNED1;MTERFD2;MTERFD2*
cg21304158	0.1824	0.1427	*SNED1*
cg05078238	-0.1807	0.1466	*SNED1*
cg25440278	0.1730	0.1648	*SNED1*
cg23419785	0.1723	0.1665	*SNED1*
cg11356990	0.1418	0.2562	*SNED1*
cg16937168	0.1288	0.3026	*SNED1*
cg16674475	0.1246	0.3189	*SNED1*
cg11769456	-0.1087	0.3851	*SNED1*
cg03785076	0.1069	0.3931	*SNED1*
cg21304813	0.0999	0.4247	*SNED1*
cg15853169	0.0982	0.4329	*SNED1*
cg05647431	0.0949	0.4485	*SNED1*
cg15550398	-0.0746	0.5516	*SNED1*
cg24407092	0.0735	0.5575	*MTERFD2;MTERFD2;SNED1;MTERFD2*
cg24269863	0.0631	0.6150	*MTERFD2;SNED1;MTERFD2;MTERFD2*

cg15133301	0.0588	0.6389	*SNED1*
cg20363891	-0.0568	0.6508	*SNED1*
cg17257554	0.0456	0.7162	*SNED1*
cg23752696	0.0413	0.7423	*SNED1*
cg05465348	0.0321	0.7981	*SNED1*
cg27472905	-0.0313	0.8027	*SNED1*
cg08233654	-0.0292	0.8160	*SNED1*
cg14454796	-0.0106	0.9325	*MTERFD2;MTERFD2;SNED1;MTERFD2*
cg04596954	0.0033	0.9792	*SNED1*
cg08377550	-0.0019	0.9881	*MTERFD2;MTERFD2;SNED1;MTERFD2*

B. Associations of methylation beta-values of individual probes in *SNED1* with log(IC50) of the HDAC inhibitor vorinostat (NSC 701852)

Probe	Spearman ρ	p_O	Gene annotation according to the UCSC Genome Browser
cg25440278	0.4268	0.0004	*SNED1*
cg01661844	0.3850	0.0014	*SNED1*
cg06127335	0.3536	0.0036	*SNED1*
cg09991306	0.3361	0.0058	*SNED1*
cg26799999	0.3213	0.0085	*SNED1*
cg10717312	0.3132	0.0105	*SNED1*
cg26707709	0.3117	0.0109	*SNED1*
cg17053285	0.2928	0.0170	*SNED1*
cg19075225	0.2809	0.0223	*SNED1*
cg26058474	0.2797	0.0229	*SNED1*
cg08557757	0.2781	0.0238	*SNED1*
cg22635676	0.2751	0.0254	*SNED1*
cg19390934	0.2644	0.0319	*MTERFD2;SNED1;MTERFD2;MTERFD2*
cg26718213	0.2635	0.0325	*SNED1*
**cg13178916**	**0.2507**	**0.0424**	***SNED1***
cg07098762	0.2372	0.0552	*SNED1*
cg12047959	0.2364	0.0560	*SNED1*
cg15133301	0.2309	0.0622	*SNED1*
cg04596954	0.2126	0.0865	*SNED1*
cg05812899	0.2125	0.0866	*SNED1*
cg25241559	0.2117	0.0880	*SNED1*
cg24912802	0.2099	0.0907	*SNED1*
cg16674475	0.2089	0.0923	*SNED1*
cg07612300	-0.2077	0.0942	*MTERFD2;MTERFD2;SNED1;MTERFD2*
cg15853169	-0.2007	0.1062	*SNED1*
cg04398322	0.1958	0.1152	*SNED1*
cg26464998	0.1933	0.1199	*SNED1*
cg15550398	-0.1930	0.1205	*SNED1*
cg03087247	0.1914	0.1237	*SNED1*
cg11207793	0.1885	0.1295	*SNED1*
cg24401641	0.1879	0.1309	*SNED1*
cg05854427	0.1855	0.1358	*SNED1*
cg02743576	0.1841	0.1389	*SNED1*
cg25850181	0.1828	0.1419	*SNED1*
cg14485214	0.1824	0.1427	*SNED1*
cg07644939	0.1773	0.1544	*SNED1*
cg11642880	-0.1737	0.1630	*SNED1*
cg04481534	0.1686	0.1761	*SNED1*
cg14574514	0.1658	0.1834	*SNED1*

cg23419785	0.1535	0.2186	*SNED1*
cg14767619	0.1533	0.2191	*SNED1*
cg21304813	-0.1506	0.2275	*SNED1*
cg01121603	0.1503	0.2284	*SNED1*
cg21773665	0.1492	0.2317	*MTERFD2;SNED1;MTERFD2;MTERFD2*
cg18663232	-0.1492	0.2319	*MTERFD2;MTERFD2;SNED1;MTERFD2*
cg14877755	0.1421	0.2551	*SNED1*
cg23752696	0.1381	0.2688	*SNED1*
cg04372536	0.1284	0.3042	*SNED1*
cg24269863	-0.1250	0.3172	*MTERFD2;SNED1;MTERFD2;MTERFD2*
cg03785076	-0.1239	0.3216	*SNED1*
cg14454796	0.1226	0.3267	*MTERFD2;MTERFD2;SNED1;MTERFD2*
cg08565567	0.1204	0.3358	*SNED1*
cg19892115	-0.1190	0.3414	*MTERFD2;MTERFD2;SNED1;MTERFD2*
cg02098967	0.1153	0.3564	*SNED1*
cg12964210	0.1088	0.3843	*SNED1*
cg03915707	0.1088	0.3844	*SNED1;SNED1*
cg15361291	0.1054	0.3996	*SNED1*
cg21304158	0.1041	0.4056	*SNED1*
cg15120952	0.1014	0.4181	*SNED1*
cg13850887	0.0989	0.4293	*SNED1*
cg08377550	-0.0925	0.4600	*MTERFD2;MTERFD2;SNED1;MTERFD2*
cg06741256	0.0922	0.4616	*SNED1*
cg17257554	-0.0849	0.4979	*SNED1*
cg24407092	-0.0847	0.4992	*MTERFD2;MTERFD2;SNED1;MTERFD2*
cg27472905	0.0842	0.5014	*SNED1*
cg09409209	0.0836	0.5045	*SNED1*
cg20363891	-0.0832	0.5066	*SNED1*
cg15396252	0.0801	0.5226	*SNED1*
cg05078238	-0.0766	0.5412	*SNED1*
cg03432919	0.0748	0.5506	*SNED1*
cg01305291	0.0699	0.5768	*SNED1*
cg06044455	-0.0672	0.5917	*SNED1;SNED1*
cg18467041	0.0657	0.6002	*SNED1*
cg11356990	0.0652	0.6029	*SNED1*
cg09028445	0.0646	0.6064	*SNED1*
cg18624016	0.0640	0.6098	*SNED1*
cg16937168	0.0635	0.6126	*SNED1*
cg07834788	0.0631	0.6146	*SNED1*
cg21239079	0.0590	0.6381	*SNED1;SNED1*
cg17685017	0.0554	0.6589	*SNED1*
cg11769456	-0.0489	0.6966	*SNED1*
cg19322479	0.0469	0.7085	*SNED1*

cg08233654	-0.0440	0.7260	*SNED1*
cg23492351	0.0423	0.7357	*SNED1*
cg13206794	0.0406	0.7462	*SNED1*
cg14359606	0.0390	0.7557	*SNED1*
cg05647431	-0.0190	0.8796	*SNED1*
cg08400319	0.0189	0.8803	*SNED1*
cg10314439	-0.0167	0.8941	*SNED1*
cg12654776	0.0086	0.9456	*SNED1*
cg05465348	-0.0079	0.9498	*SNED1*
cg02225728	0.0030	0.9811	*SNED1*

C. Associations of *SNED1* transcript expression with response to HDAC inhibitors

Shown below are Pearson correlations of the *SNED1* transcript NM_001080437 (Affymetrix cluster ID 2536071) with log(IC50) of HDAC inhibitors satisfying $p < 0.05$ (shown by an asterisk), and also associations with response to vorinostat and 4SC-202, which did not reach statistical significance. With the exception of simvastatin, all associations shown below indicate a weak trend for resistance to HDAC inhibitors for the cell lines with increased *SNED1* expression.

**NSC**	**Agent**	**Pearson *r***	***p***
758774	Belinostat	0.2971	0.0146*
758249	SB-939	0.2693	0.0275*
759657	JNJ-26481585	0.2612	0.0327*
761190	Panobinostat	0.2598	0.0337*
751549	Simvastatin	-0.2448	0.0459*
701852	Vorinostat	0.2129	0.0837
759905	4SC-202	0.1492	0.2281

r, Pearson correlation coefficient; *p, p*-value for the correlation between *SNED1* transcript expression and log(IC50) of HDAC inhibitors
* indicates $p < 0.05$

D. Additional information about associations of probes in selected genes

The chromosomal region 2q37.3 containing *SNED1* is recurrently deleted in cancer [47]. The number of cell lines with *SNED1* copy number loss in our data was small, and no significant conclusions could be drawn about whether *SNED1* copy number loss was associated with response to the agents 4SC-202, BAL-101553, CP-868596, CYT-997, GSK-461364, SNS-314, or vinblastine, which were the most strongly correlated with probe methylation. Similarly, no significant conclusions could be drawn about whether the *SNED1* copy number loss in our dataset was associated with methylation of the *SNED1* probes cg13178916, cg22635676, cg17053285, or cg03087247. These four probes were the most strongly correlated with response to these agents among the *SNED1* probes ($p_O < 5 \times 10^{-7}$; data not shown).

At the epigenome-wide level, the probes cg00870242, cg03790427, cg14182145, and cg15816503 in *C8orf74* were associated with response to BIM-46187 with $p_O < 9.42 \times 10^{-8}$ (**Supplementary Table 3**).

At the epigenome level, a strong correlation of methylation of the probe cg13042130 in the body of *BCL2* with resistance to the CDK inhibitor R-547 did not reach the stringent significance threshold ($\rho = 0.5901$, $p_O = 1.85 \times 10^{-7}$; data not shown). In our analysis of candidate genes and preselected agents, five probes in the gene body of *BCL2* were relatively strongly associated with Bcl inhibitors GX15-070 and ABT-737 and the Aurora A kinase/tyrosine kinase inhibitor ENMD-2076 under a relaxed threshold of $p_{FDR} < 0.15$ ($0.4481 \leq |\rho| \leq 0.5036$, $1.63 \times 10^{-5} \leq p_O \leq 3.22 \times 10^{-5}$, $0.1134 \leq p_{FDR} \leq 0.1336$; **Supplementary Tables 6 and 7**). The direction of these correlations was probe-specific, suggesting variable methylation patterns within *BCL2* (**Supplementary Table 7)**.

In the candidate gene analysis, 13 *EPAS1* probes had modest or strong correlations with resistance to AMG-900 ($\rho > 0.4$ for cg00555811, cg00793901, cg05943554, cg06087988, cg08937075, cg11108228, cg11369576, cg13681847, cg14358543, cg15723976, cg17035547, cg21494367, and cg22939839; data not shown).

The TSS1500 and the TSS200 regions of *EZH2* had very low methylation (beta-values ≤ 0.1835 and 0.0515, respectively). The 5'UTR of *EZH2* was more heavily methylated (SCLC cell line beta-values between 0.2918 and 0.4209) and that region and several individual probes located within it were associated with drug response in the candidate gene analysis. Among the probes in the 5'UTR, cg08558971 was associated with AMG-900, CYC-116, and SNS-314 ($0.4927 \leq \rho \leq 0.5464$, $2.08 \times 10^{-6} \leq p_O \leq 2.64 \times 10^{-5}$, $0.0515 \leq p_{FDR} \leq 0.1285$; **Table 3, Supplementary Tables 6 and 7**). That probe and the probes cg24190072, and cg04376478 in the 5'UTR of *EZH2* were also correlated with resistance to ABT-348, AZD-1152, MLN-8237, BEZ-235, BGJ-398, CYC-116, and sapanisertib, however those correlations were not significant after the adjustment for multiple testing ($0.4262 \leq \rho \leq 0.4988$, $5.57 \times 10^{-5} \leq p_O \leq 0.0004$, $p_{FDR} > 0.15$; data not shown).
