# Supplementary material for: Epigenome-wide DNA methylation analysis of small cell lung cancer cell lines suggests potential chemotherapy targets
Source: Clin Epigenetics. 2020 Jun 25;12:93. doi: 10.1186/s13148-020-00876-8 (PMC7318526; doi:10.1186/s13148-020-00876-8)
Supplement: Supplementary file 11 [file 13148_2020_876_MOESM13_ESM.docx]

A **Methylation of 3' UTR of *CEP350* vs log(IC50) of ABT-348**

$\rho = 0.610, p_O = 5.54 \times 10^{-8}$

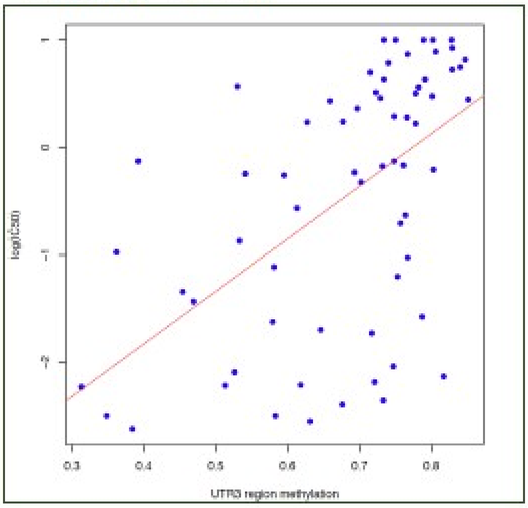

B **Methylation of 3' UTR of *CEP350* vs log(IC50) of TAK-960**

$\rho = 0.595, p_O = 1.35 \times 10^{-7}$

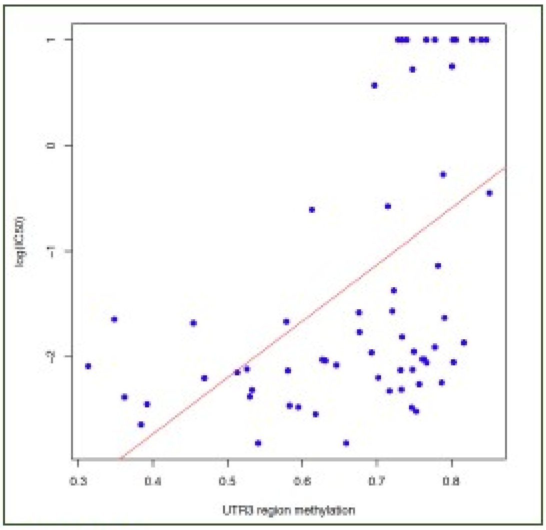

Supplementary Fig. 1
