# Supplementary material for: Epigenome-wide DNA methylation analysis of small cell lung cancer cell lines suggests potential chemotherapy targets
Source: Clin Epigenetics. 2020 Jun 25;12:93. doi: 10.1186/s13148-020-00876-8 (PMC7318526; doi:10.1186/s13148-020-00876-8)
Supplement: Supplementary file 12 [file 13148_2020_876_MOESM14_ESM.docx]

A **Methylation of the *EPAS1* probe cg08937075 vs log(IC50) of AMG-900**

$\rho = 0.628, p_O = 1.68 \times 10^{-8}$

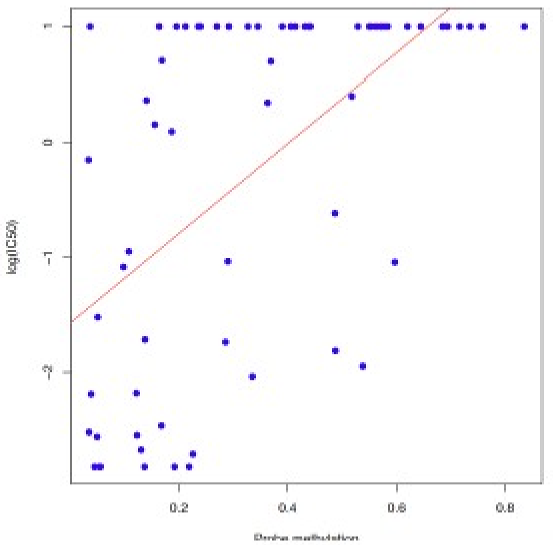

B **Methylation of the *EPAS1* probe cg08937075 vs *EPAS1* transcript 2480383 expression**

$\rho = 0.680, p_O = 3.39 \times 10^{-10}$

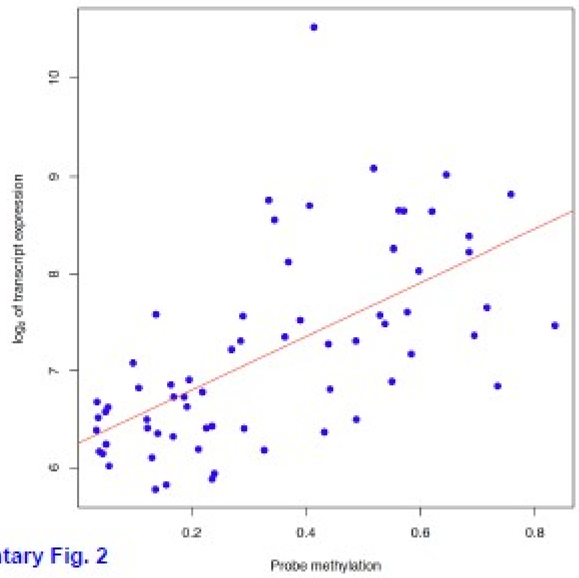

Supplementary Fig. 2
