# Supplementary material for: Epigenome-wide DNA methylation analysis of small cell lung cancer cell lines suggests potential chemotherapy targets
Source: Clin Epigenetics. 2020 Jun 25;12:93. doi: 10.1186/s13148-020-00876-8 (PMC7318526; doi:10.1186/s13148-020-00876-8)
Supplement: Supplementary file 14 [file 13148_2020_876_MOESM16_ESM.docx]

A **Methylation of the 5'UTR of *EZH2* vs log(IC50) of ABT-348**

$\rho = 0.436, p_O = 0.0003$

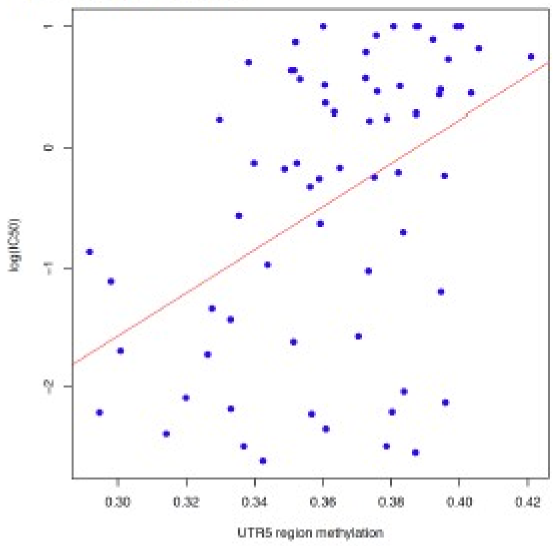

B **Methylation of 5'UTR of *EZH2* vs log(IC50) of CT-32228**

$\rho = 0.581, p_O = 3.08\times10^{-7}$

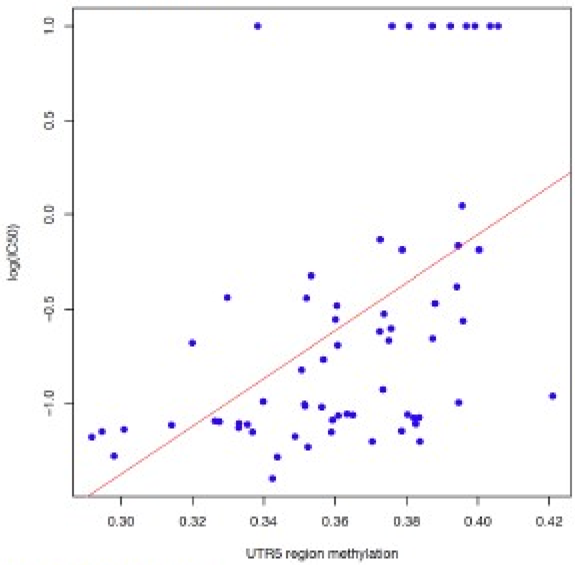

Supplementary Fig. 4

C

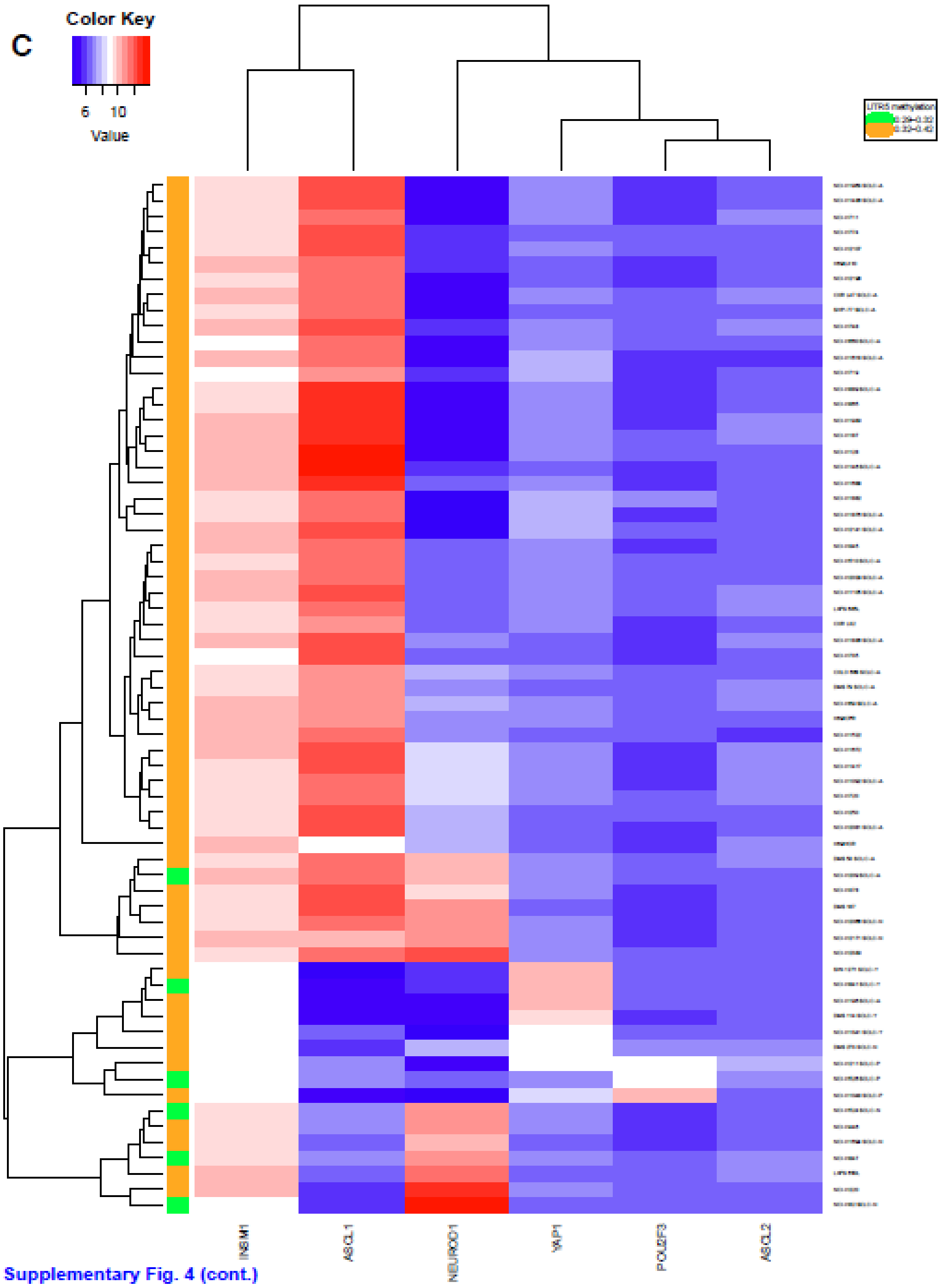

Supplementary Fig. 4 (cont.)

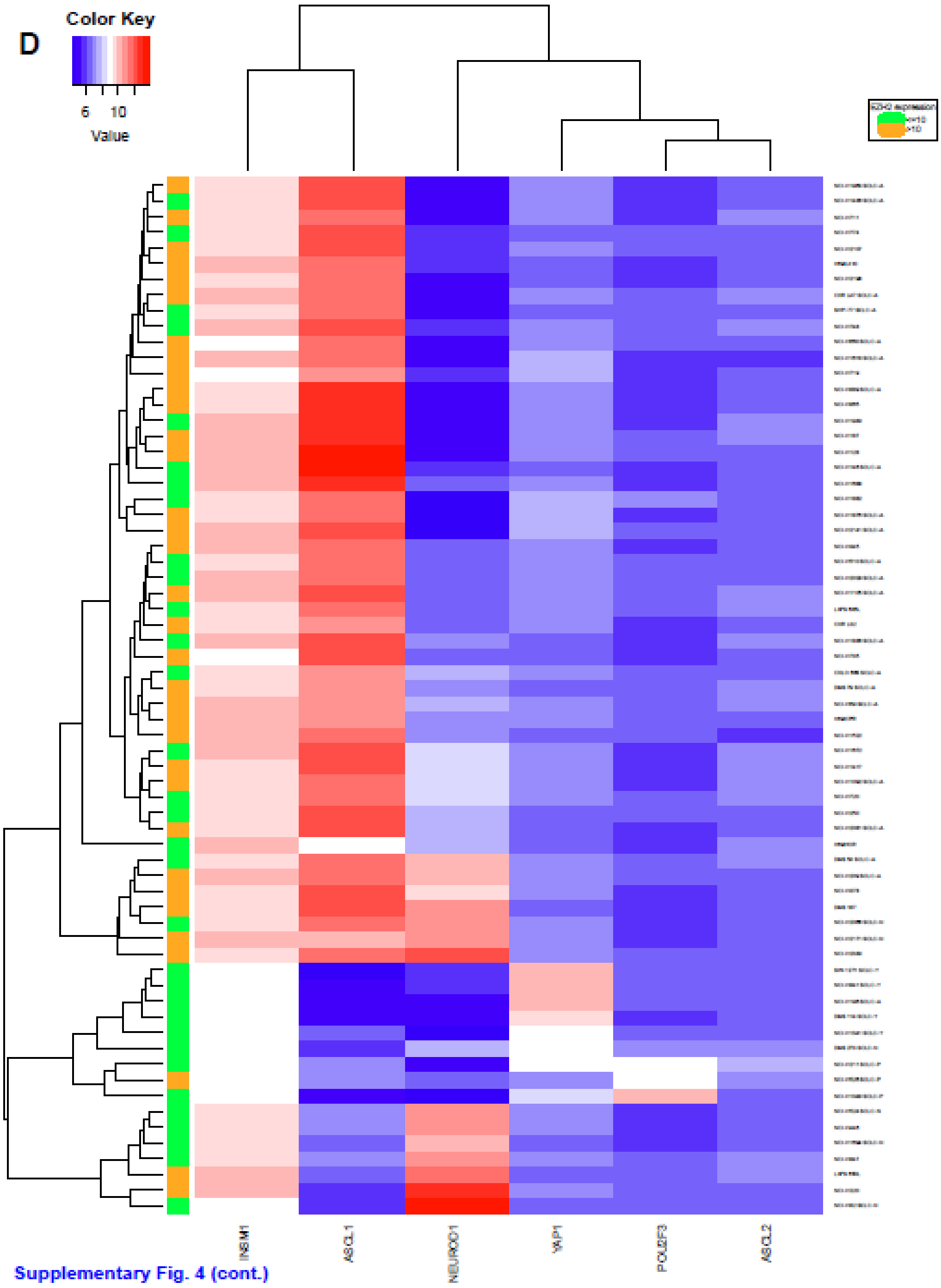

Supplementary Fig. 4 (cont.)
